# Changes and Trend Disparities in Life Expectancy and Health-Adjusted Life Expectancy Attributed to Disability and Mortality From 1990 to 2019 in China

**DOI:** 10.3389/fpubh.2022.925114

**Published:** 2022-07-18

**Authors:** Lijun Chen, Lu Wang, Yun Qian, Hai Chen

**Affiliations:** Department of Chronic Disease Control, Wuxi Center for Disease Control and Prevention, Wuxi, China

**Keywords:** life expectancy, health-adjusted life expectancy, disability effect, mortality effect, decomposition

## Abstract

**Objective:**

This study aims to investigate sex, age, and cause-specific contributions to changes and trend disparities in life expectancy (LE) and health-adjusted life expectancy (HALE) attributed to disability and mortality from 1990 to 2019 in China, which provides insight into policy-making, health systems planning, and resource allocation.

**Methods:**

Contributions of disability and mortality to changes and trend disparities in LE and HALE were estimated with standard abridged life table, Sullivan's method, and decomposition method, using retrospective demographic analysis based on mortality and years lived with disability (YLD) rates extracted from Global Burden of Disease Study 2019 (GBD 2019).

**Results:**

From 1990 to 2019, LE and HALE increased by 10.49 and 8.71 years for both sexes, mainly due to noncommunicable diseases (NCDs) (5.83 years, 55.58% for LE and 6.28 years, 72.10% for HALE). However, HIV/AIDS and sexually transmitted infections had negative effects on changes in LE (−0.03 years, −0.29%) and HALE (−0.05 years, −0.57%). Lung cancer and ischemic heart disease caused the biggest reduction in LE (−0.14 years, −1.33%) and HALE (−0.42 years, −4.82%). Also, cardiovascular diseases (−0.08 years, −0.92%), neurological disorders (−0.08 years, −0.92%), diabetes and kidney diseases (−0.06 years, −0.69%), and transport injuries (−0.06 years, −0.69%) had main negative disability effects in HALE. Moreover, life expectancy lived with disability (LED) increased by 1.78 years, mainly attributed to respiratory infections and tuberculosis (1.04 years, 58.43%) and maternal and neonatal disorders (0.78 years, 43.82%).

**Conclusion:**

The LE and HALE in China have grown rapidly over the past few decades, mainly attributed to NCDs. It is necessary to further reduce the negative mortality effect of HIV/AIDS, lung cancer, colon and rectum cancer, pancreatic cancer, and ischemic heart disease and the negative disability effect of stroke, diabetes mellitus, and road injuries. In addition, the signs of disparities in mortality and disability of different sexes and ages call for targeted and precise interventions for key groups such as males and the elderly. According to the decomposition results, we may better determine the key objects of health policies that take into account substantial cause-specific variations to facilitate the realization of “healthy China 2030” plan.

## Introduction

Life expectancy (LE) is one of the most commonly used population health summary indicators, which is based on a set of observed age-specific mortalities (1). Unlike LE, health-adjusted life expectancy (HALE) is more comprehensive and captures both the length and the quality of life (2, 3). LE and HALE are important indicators of socioeconomic and medical development, which had increased steadily in the past several years in China due to improvements in living conditions, education, and healthcare practices (4, 5). However, HALE has grown at a slower rate than LE, resulting in longer LE for patients with disabilities (LED) (6, 7).

Due to population aging and urbanization, the leading causes of death in China have undergone an epidemiological transition from communicable, maternal, neonatal, and nutritional diseases (CMNNs) to noncommunicable diseases (NCDs) (8, 9). China is rapidly catching up with this epidemic, like most developed countries (10). Despite China's progress in improving health, NCDs were still responsible for 1.65 million (88%) of China's total deaths in 2019, including leading contributors to mortality and disability such as cardiovascular diseases (0.88 million, 46%) and cancer (0.45 million, 24%) (11). In addition, far less is known about cause-specific trends in mortality and disability, and detailed information on sex, age, and cause-specific LE obtained in LE and HALE is not yet well characterized. The question of whether some population groups or causes remain disadvantaged and left behind as LE and HALE grow should be focused on.

While cause-eliminated life tables are often used to evaluate the impact of different causes on LE and HALE (12, 13), this approach only focuses on the cross-sectional study and is unable to access the impact on changes. Thus, we used a decomposition method, which was more comparative and allowed assigned contributions of mortality and disability variation to specific age groups or causes of death (14). Additionally, the advantages of using data from the Global Burden of Disease (GBD) study, which involved comprehensive data sources and methods, were obvious.

The Chinese government has attached great importance to population health. The Healthy China 2030 plan was implemented as a national health policy in 2016 (15). Two of the goals are to improve LE and HALE. At present, a comprehensive and comparable assessment of cause-specific effects on LE and HALE and how they change over time is not available. Understanding changes and trend disparities in LE and HALE at the national level is crucial to tracking progress toward the goals and promoting population wellbeing. Thus, this study aimed to investigate sex, age, and cause-specific contributions to changes and trend disparities in LE and HALE attributed to disability and mortality in China from 1990 to 2019, thereby providing insights into policy-making, health systems planning, and resource allocation.

## Materials and Methods

### Data Sources

All the data for the period 1990–2019 in China, as tabulated in different causes, sexes, and age groups (<1 year, 1–4 years, 5–9 years, ..., 95+ years), were retrieved from the Global Burden of Disease Study 2019 (GBD 2019) (10), including mortality and years lived with disability (YLD) rates. The main data sources are the China Disease Surveillance Points (DSP) system, Maternal and Child Surveillance System, Cause of Death Reporting System of Chinese Center for Disease Control and Prevention (CDC), and Cancer Registry data. The methods for data collecting, processing, and analyzing are briefly described below, and the details can be found in the GBD 2019 (16, 17). A crucial step in data processing is correcting for known bias by redistributing deaths from unspecified codes to more specific disease categories, and by adjusting data with alternative case definitions or measurement methods to the reference method. Another important aspect is that cause-specific estimates are modeled using standardized tools such as the Cause of Death Ensemble model (CODEm), spatiotemporal Gaussian process regression (ST-GPR), and DisMod-MR to generate estimates of each quantity of interest by age, sex, location, and year.

Causes of death are organized in a hierarchical list containing 3 levels. At the highest level (Level 1), all causes are divided into three mutually exclusive and collectively exhaustive categories, namely, CMNNs, NCDs, and injuries. Level 2 contains 22 cause groups, such as cardiovascular diseases, neoplasms, and transport injuries. Level 3 disaggregates these causes further, including 168 more specific causes. The corresponding International Classification of Diseases (ICD9 and ICD10) codes are detailed in Supplementary Table S1.

### Statistical Analysis

The LE at birth was computed with a standard abridged life table (18), using mortality in the infant age group (<1 year) and 19 non-infant age groups starting with age 1–4 years, then proceeding in 5-year age groups (5–9 years, 10–14 years, …) until the terminal age group of 95+ years. Next, the probability of dying, the number of individuals alive, and the number of person-years lived in each age group were computed. HALE refers to the number of years that a person can expect to live in good health, taking into account both mortality and disability, which were computed with Sullivan's method based on the standard abridged life table (19). YLD rates were used to represent the disability prevalence.


$$\begin{array}{l} \begin{array}{lll} LE(x) & = & \frac{\sum_{i=x}^{n}\times L_{x}}{l_{x}}\\ HALE(x) & = & \frac{\sum_{i=x}^{n}(1-YLD_{x})\times L_{x}}{l_{x}} \end{array} \end{array}$$

where *n* is the last age group in the life table; *l*_*x*_ is the number of individuals alive at age *x*; *L*_*x*_ is the number of person-years lived in age group; *YLD*_*x*_ is all-cause disability prevalence in age group.

Contributions to the changes in LE were estimated with Arriaga's decomposition method (20, 21), while contributions to the changes in HALE were estimated with an extended method based on Arriaga (22). LE and HALE changes in the period of 1990–2019 and 10-year intervals (1990–1999, 2000–2009, and 2010–2019) were both decomposed. All the analyses were carried out with R version 3.4.1 (R Foundation for Statistical Computing, Vienna, Austria). Detailed procedures and formulas are described below.

Step 1: To decompose LE by age group. The total contribution of an age group to the LE gap (in years) is the sum of two mathematical terms. The first term represents the direct effect of age group, which consists of the years an age group contributes to LE changes. The second term represents indirect and interaction effect. For example, higher mortality in the 30–34 years age group may leave fewer survivors at the age of 35 years.


$$\begin{array}{l} \text{Δ}LE_{x}=TE_{x}=\left[\frac{l_{\;x}^{\;A}}{l_{0}}\times \left(\frac{L_{x}^{B}}{l_{x}^{\;B}}-\frac{L_{x}^{A}}{l_{x}^{\;A}}\right)\right]\;\\ \;+\left[\frac{T{\;}_{x+n}^{\;B}}{l_{0}}\times \left(\frac{l{\;}_{x}^{A}}{l_{x}^{\;B}}-\frac{l_{\;x+n}^{\;A}}{l_{x+n}^{\;B}}\right)\right] \end{array}$$

Step 2: To decompose HALE by age group. The total contribution of an age group to the HALE gap (in years) is also the sum of two mathematical terms. The first term represents the mortality effect of age group (ME), which is the changes in HALE caused by the changes in person-years lived in age group. The second term represents the disability effect (DE), which is the changes in HALE caused by the changes in YLD rates in age group.


$$\begin{array}{l} \text{Δ}HALE_{x}=TE_{x}=\left[\frac{YLD_{x}^{\;A}+YLD_{x}^{\;B}}{2\times l_{0}}\times \left(L_{x}^{B}-L_{x}^{A}\right)\right]\;\\ \;+\left[\frac{L_{x}^{A}+L_{x}^{B}}{2\times l_{0}}\times \left(YLD_{x}^{\;B}-YLD_{x}^{\;A}\right)\right] \end{array}$$

Step 3: To decompose LE by cause-specific death within an age group. The total contribution of a given age group to the LE gap (in years) is further partitioned into the years contributed by each cause. The total contribution of each cause to the LE gap is obtained by summing cause-specific contributions across all age groups.


$$\begin{array}{l} {\text{Δ}LE_{x}^{\;i}=TE}_{x}^{i}=TE_{x}\times \left[\frac{R_{x}^{\;i,\;B}-R_{x}^{\;i,\;A}}{S_{x}^{\;B}-S_{x}^{\;A}}\right] \end{array}$$

Step 4: To decompose HALE by cause-specific death within an age group. The mortality and disability effect of a given age group to the HALE gap (in years) is further partitioned into the years contributed by each cause based on the specific percentage of gap of mortality or YLD rates in the total change. The total contribution of each cause to the HALE gap is also obtained by summing cause-specific mortality and disability effect across all age groups.


$$\begin{array}{l} \text{Δ}HALE_{x}^{\;i}=TE_{x}^{\;i}=ME_{x}^{\;i}+DE_{x}^{\;i}\;\\ \;ME_{x}^{\;i}=\left[\frac{YLD_{x}^{\;A}+YLD_{x}^{\;B}}{2\times l_{0}}\times \left(L_{x}^{B}-L_{x}^{A}\right)\right]\times \left[\frac{R_{x}^{\;i,\;B}-R_{x}^{\;i,\;A}}{S_{x}^{\;B}-S_{x}^{\;A}}\right]\\ \;DE_{x}^{\;i}\;=\left[\frac{L_{x}^{A}+L_{x}^{B}}{2\times l_{0}}\times \left(YLD_{x}^{\;B}-YLD_{x}^{\;A}\right)\right]\;\\ \;\times \;\left[\frac{YLD_{x}^{\;i,\;B}-YLD_{x}^{\;i,\;A}}{V_{x}^{\;B}-V_{x}^{\;A}}\right] \end{array}$$

where *TE*_*x*_ is the total contribution between ages *x* and *x*+*n, l*_*x*_ is the number of individuals alive at age *x, l*_0_is the cohort size at the start, *L*_*x*_ is the number of person-years lived between ages *x* and *x*+*n*, and *T*_*x*+*n*_ is the total number of person-years lived above age x+n. *l*_*x*+*n*_ is the number of individuals alive at age *x*+*n*. $YLD_{x}^{\;}\;$is the disability prevalence at age *x*. $TE_{x}^{\;i}$ is the total contribution between ages *x* and *x*+*n* due to cause *i*, $R_{x}^{\;i}$ is the specific mortality rate between ages *x* and *x*+*n* due to cause *i*. $S_{x}^{\;}$ is the all-cause mortality rate between ages *x* and *x*+*n*. $YLD_{x}^{\;i}$ is the specific disability prevalence between ages *x* and *x*+*n* due to cause *i*. $V_{x}^{\;}$ is the all-cause YLD rate between ages *x* and *x*+*n*. “A” and “B” represent different years.

## Results

### Changes in LE and HALE

**Figure 1:**
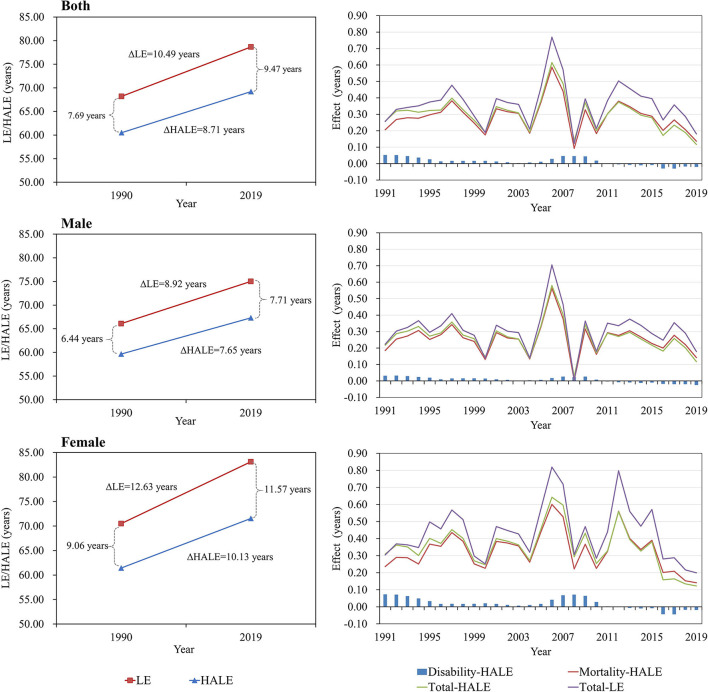
Trends and effects of mortality and disability on changes in life expectancy (LE) and health-adjusted life expectancy (HALE) across year to year.

From 1990 to 2019, LE increased by 10.49 years for both sexes, while HALE increased by 8.71 years as a result of reduced mortality (8.30 years, 95.29%) and disability (0.41 years, 4.71%). LED was the difference between LE and HALE, with an average increase of 1.78 years for both sexes (Table 1). The total effect on the change in LE and HALE was greater for females than for males with higher LEDs. LE and HALE showed an increasing trend every year, varying significantly from year to year, with the overall effect being positive (Figure 1). For changes in HALE, the disability effects of all causes were at a low level, with both males and females showing a negative effect after 2011. In addition, from 1990 to 1999, LE and HALE increased by 3.20 and 2.85 years for males and females. Starting in 2000, LE and HALE further increased by 3.65 and 3.16 years for both sexes. However, the growth rate has decreased since 2010, resulting in 3.24 and 2.30 years for both sexes, respectively, until 2019, especially in terms of the total effect of HALE changes. From 2010 to 2019, a negative effect of disability was observed. Thus, LED increased from 0.35 to 0.94 years for both sexes in the past three decades, and females also had a higher total effect of changes in LE and HALE than males with a higher LED in each decade (Table 2).

**Table 1:** Effects of mortality and disability on changes in life expectancy (LE) and health-adjusted life expectancy (HALE) (years) from 1990 to 2019.

**Population**	**ΔLE**	**ΔHALE**			**ΔLED**
	**TE**	**ME**	**DE**	**TE**	
Both sexes	10.49	8.30	0.41	8.71	1.78
Males	8.92	7.42	0.23	7.65	1.27
Females	12.63	9.53	0.60	10.13	2.51

*TE, total effect; ME, mortality effect; DE, disability effect*.

**Table 2:** Effects of mortality and disability on changes in life expectancy (LE) and health-adjusted life expectancy (HALE) (years) for 10-year interval.

**Intervals**	**Population**	**ΔLE**	**ΔHALE**			**ΔLED**
		**TE**	**ME**	**DE**	**TE**	
1990–1999	Both sexes	3.20	2.57	0.28	2.85	0.35
	Males	2.84	2.39	0.20	2.59	0.25
	Females	3.72	2.85	0.37	3.22	0.50
2000–2009	Both sexes	3.65	2.96	0.20	3.16	0.49
	Males	3.00	2.53	0.12	2.65	0.35
	Females	4.56	3.54	0.30	3.84	0.72
2010–2019	Both sexes	3.24	2.43	−0.13	2.30	0.94
	Males	2.76	2.21	−0.12	2.09	0.67
	Females	3.83	2.71	−0.14	2.57	1.26

*TE, total effect; ME, mortality effect; DE, disability effect*.

### Mortality and Disability Effect on Changes in LE and HALE

From 1990 to 2019, better control of NCDs contributed most to changes in LE (5.83 years, 55.58%) and HALE (6.28 years, 72.10%) for both sexes, followed by control of CMNNs (3.46 years, 32.98% for LE and 1.32 years, 15.15% for HALE) and injuries (1.20 years, 11.44% for LE and 1.11 years, 12.74% for HALE) (Table 3). For level 2 causes of CMNNs, respiratory infections and tuberculosis contributed most to the changes in LE (1.86 years, 17.73%) and HALE (0.82 years, 9.41%). Only HIV/AIDS and sexually transmitted infections had negative total effects both on changes in LE (−0.03 years, −0.29%) and HALE (−0.05 years, −0.57%) because of the negative effect of mortality. HALE also decreased by 0.09 years (1.03%) owing to a reduction in the disability of maternal and neonatal disorders. For level 2 causes of NCDs, chronic respiratory diseases contributed most to the changes in LE (2.02 years, 19.26%) and HALE (2.83 years, 32.49%), followed by cardiovascular diseases (1.77 years, 16.87% for LE and 1.82 years, 20.90% for HALE). Moreover, 5 causes had negative disability effects on changes in HALE such as cardiovascular diseases (−0.08 years, −0.92%), neurological disorders (−0.08 years, −0.92%), and diabetes and kidney diseases (−0.06 years, −0.69%). For level 2 causes of injuries, unintentional injuries contributed the most to changes in LE (0.57 years, 5.43%), and self-harm injuries and interpersonal violence contributed most to the changes in HALE (0.59 years, 6.77%). Among them, transport injuries (−0.06 years, −0.69%) and unintentional injuries (−0.01 years, −0.11%) had negative disability effects on changes in HALE.

**Table 3:** Cause-specific effects on changes in life expectancy (LE) and health-adjusted life expectancy (HALE) from 1990 to 2019 (years, levels 1–2).

**Causes**	**Level**	**ΔHALE**			**ΔLE**	**ΔLED**
		**ME**	**TE**	**TE**	**TE**	
**Communicable, maternal, neonatal, and nutritional diseases**	A	1.09	0.23	1.32	3.46	2.14
HIV/AIDS and sexually transmitted infections	A.1	−0.05	<0.01	−0.05	−0.03	0.02
Respiratory infections and tuberculosis	A.2	0.78	0.04	0.82	1.86	1.04
Enteric infections	A.3	0.07	<0.01	0.07	0.29	0.21
Neglected tropical diseases and malaria	A.4	0.01	0.10	0.11	0.01	−0.10
Other infectious diseases	A.5	0.17	0.02	0.19	0.45	0.26
Maternal and neonatal disorders	A.6	0.07	−0.09	−0.02	0.76	0.78
Nutritional deficiencies	A.7	0.03	0.16	0.20	0.11	−0.08
**Non-communicable diseases**	B	6.05	0.23	6.28	5.83	−0.45
Neoplasms	B.1	0.71	−0.04	0.66	0.83	0.17
Cardiovascular diseases	B.2	1.84	−0.08	1.77	1.82	0.06
Chronic respiratory diseases	B.3	2.72	0.11	2.83	2.02	−0.81
Digestive diseases	B.4	0.50	0.05	0.54	0.49	−0.06
Neurological disorders	B.5	0.05	−0.08	−0.03	0.05	0.08
Mental disorders	B.6	<0.01	0.04	0.04	<0.01	−0.04
Substance use disorders	B.7	0.06	0.01	0.07	0.04	−0.02
Diabetes and kidney diseases	B.8	0.08	−0.06	0.02	0.09	0.07
Skin and subcutaneous diseases	B.9	0.01	−0.01	<0.01	0.01	0.01
Sense organ diseases	B.10	<0.01	0.05	0.05	<0.01	−0.05
Musculoskeletal disorders	B.11	<0.01	0.11	0.10	<0.01	−0.11
Other non-communicable diseases	B.12	0.08	0.14	0.22	0.48	0.26
**Injuries**	C	1.16	−0.05	1.11	1.20	0.09
Transport injuries	C.1	0.21	−0.06	0.15	0.21	0.05
Unintentional injuries	C.2	0.37	−0.01	0.36	0.57	0.21
Self-harm and interpersonal violence	C.3	0.58	0.01	0.59	0.42	−0.17

*TE, total effect; ME, mortality effect; DE, disability effect*.

**Figure 2:**
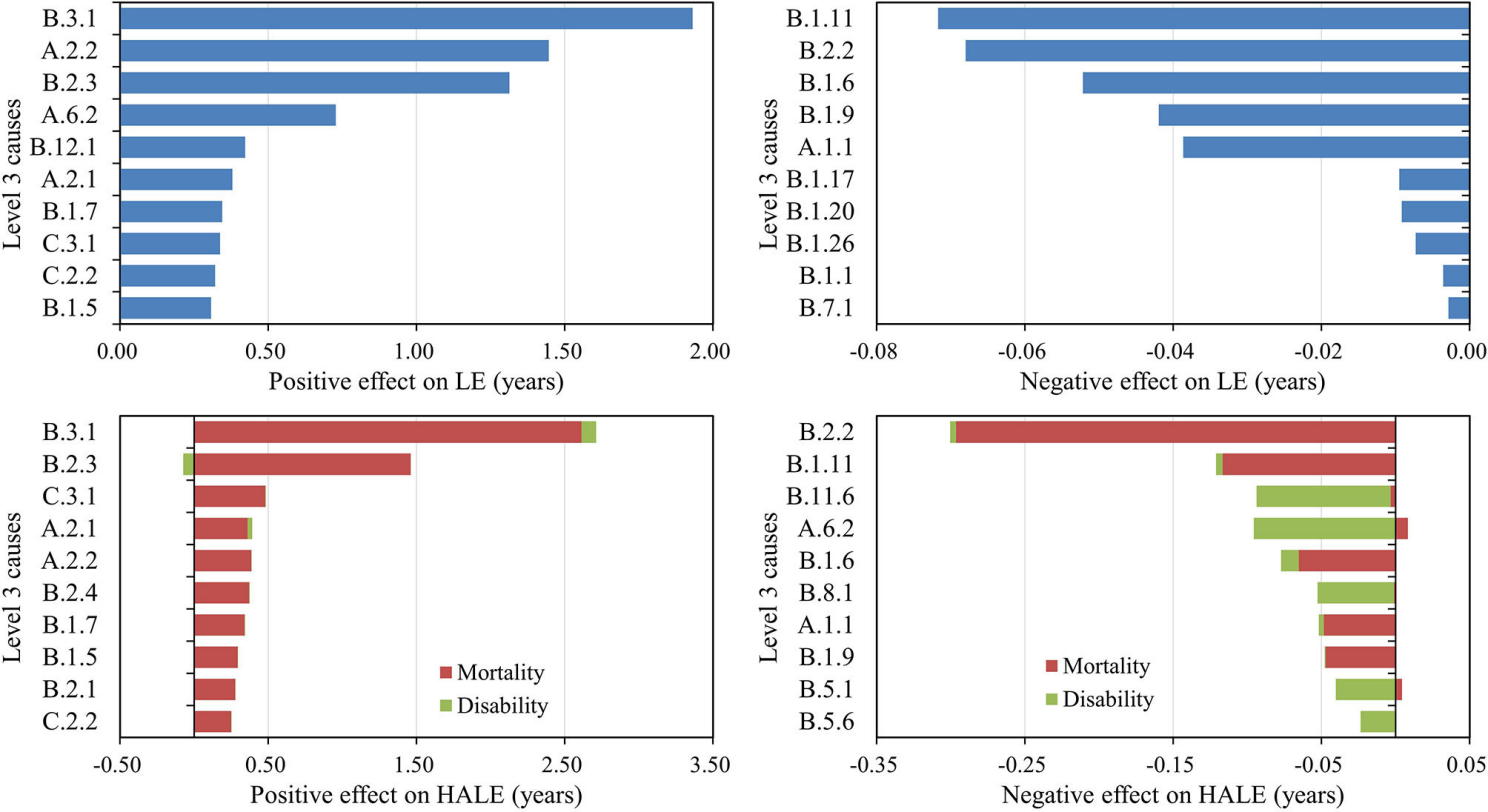
Leading causes with positive/negative effects on changes in life expectancy (LE) and health-adjusted life expectancy (HALE) from 1990 to 2019 (level 3).

Figure 2 shows the top 10 level 3 causes that positively/negatively influenced the changes in LE and HALE. Chronic obstructive pulmonary disease contributed the most to changes in LE (1.93 years, 18.40%) and HALE (2.71 years, 31.11%). More than 70% of improvement in both LE and HALE was attributed to better control of these top 10 level 3 causes, and 7 of them were shown simultaneously in both lists, including chronic obstructive pulmonary disease, stroke, lower respiratory infections, tuberculosis, stomach cancer, liver cancer, and drowning. But it is worth noting that stroke (−0.07 years, −0.80%) had a negative disability effect on changes in HALE. Lung cancer and ischemic heart disease caused the biggest reduction in LE (−0.14 years, −1.33%) and HALE (−0.42 years, −4.82%). More than 10% of the decline in HALE was attributed to poorer control of these top 10 level 3 causes with a negative effect. More details are summarized in Supplementary Table S2.

In the past three decades, the most prominent causes of the increases in LE between 1990 and 1999 were NCDs (1.59 years, 49.66%) and CMNNs (1.29 years, 40.32%), including cardiovascular diseases (0.63 years, 19.86%), chronic respiratory diseases (0.55 years, 17.27%), and respiratory infections and tuberculosis (0.76 years, 23.92%). The most prominent cause of the increases in HALE was also NCDs (1.58 years, 55.35%), followed by CMNNs (0.89 years, 31.24%). Subsequently, the positive total effects of NCDs on changes in LE increased, reaching 2.25 years between 2010 and 2019, mainly attributed to a reduction in mortality of neoplasms (0.37 years, 11.47%) and cardiovascular diseases (0.95 years, 29.27%). However, the positive total effects of NCDs on changes in HALE decreased since 2010 to 1.93 years, mainly attributed to an increase in disability rate of neoplasms (0.26 years, 11.20%), chronic respiratory diseases (0.63 years, 27.48%), and digestive diseases (0.10 years, 4.51%). For CMNNs, the positive total effects both on changes in LE and HALE decreased to a low level (0.49 years, 15.02%) from 2010 to 2019, which was mainly due to HIV/AIDS and sexually transmitted infections (−0.02 years, −0.62%) and maternal and neonatal disorders (−0.03 years, −0.93%) with negative effects. For injuries (0.50 years, 15.50%), which mainly attributed to transport injuries (−0.04 years, −1.24%) and unintentional injuries (−0.11 years, −3.40%) with negative disability effects. More details are summarized in Supplementary Tables S3–S5.

### Sex and Age-Specific Effect on Changes in LE and HALE

**Figure 3:**
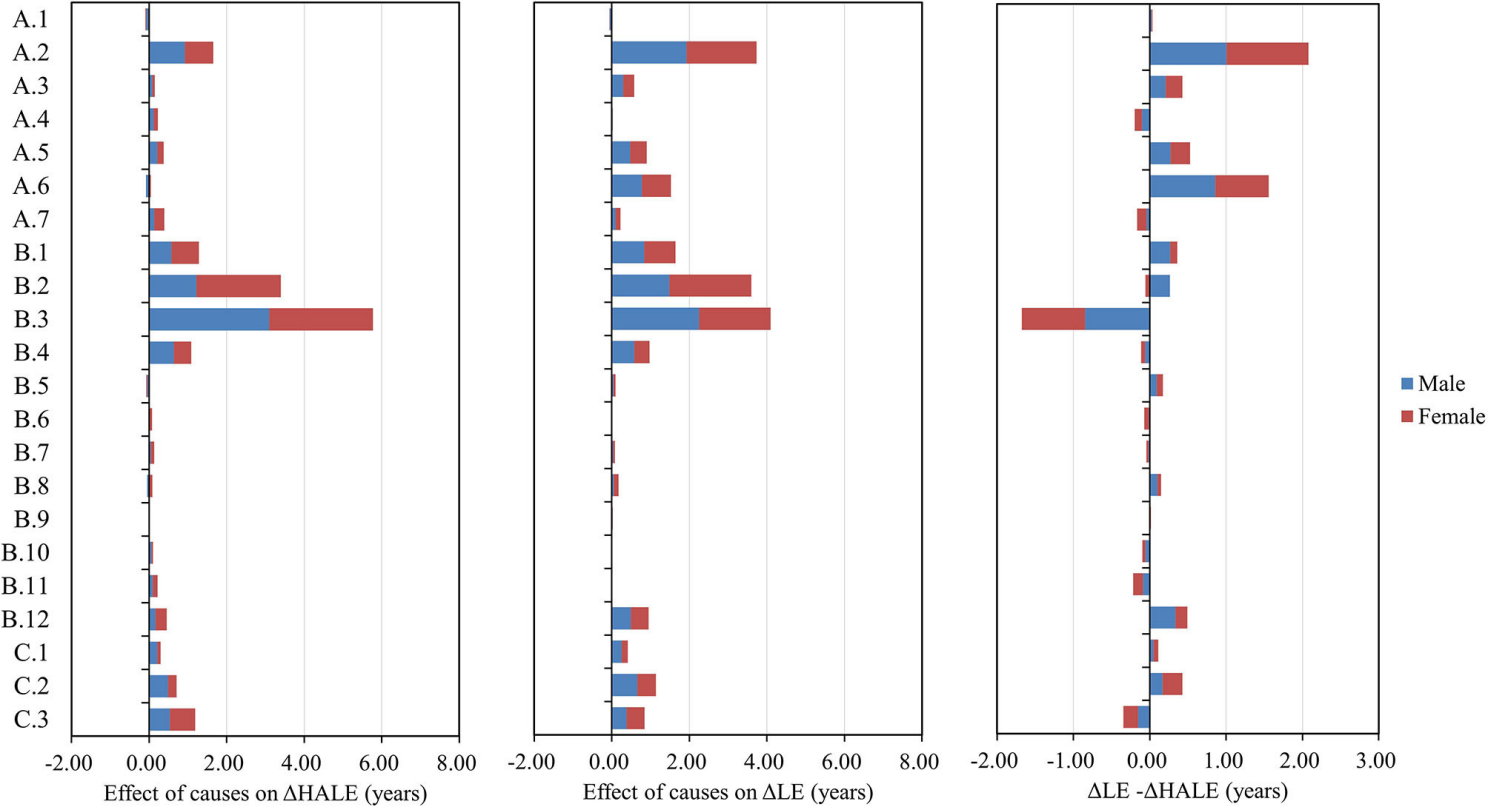
Cause-specific effects on changes in life expectancy (LE) and health-adjusted life expectancy (HALE) (levels 1–2) for males and females from 1990 to 2019.

From 1990 to 2019, better control of NCDs contributed most to the changes in LE (4.50 years, 50.45% for males; 7.68 years, 60.81% for females) and HALE (5.24 years, 68.50% for males; 7.65 years, 75.52% for females). Figure 3 shows level 2 causes with total effect on changes in LE and HALE. For males, more than 60% (5.89 years) of improvement in LE was attributed to better control of the following 5 diseases: respiratory infections and tuberculosis (1.71 years), chronic respiratory diseases (1.49 years), cardiovascular diseases (1.19 years), neoplasms (0.76 years), and maternal and neonatal disorders (0.74 years). More than 70% (5.79 years) of improvement in HALE was attributed to better control of the following 5 diseases: chronic respiratory diseases (2.53 years), cardiovascular diseases (1.21 years), respiratory infections and tuberculosis (0.83 years), neoplasms (0.62 years), and digestive diseases (0.60 years). Similarly, for females, more than 70% (9.24 years) was due to cardiovascular diseases (2.77 years), chronic respiratory diseases (2.68 years), respiratory infections and tuberculosis (2.04 years), neoplasms (0.96 years), and maternal and neonatal disorders (0.79 years). More than 75% (7.97 years) of improvement in HALE was attributed to better control of the following 5 diseases: chronic respiratory diseases (3.31 years), cardiovascular diseases (2.41 years), respiratory infections and tuberculosis (0.85 years), neoplasms (0.74 years), and self-harm and interpersonal violence (0.66 years). More details are summarized in Supplementary Table S6.

**Figure 4:**
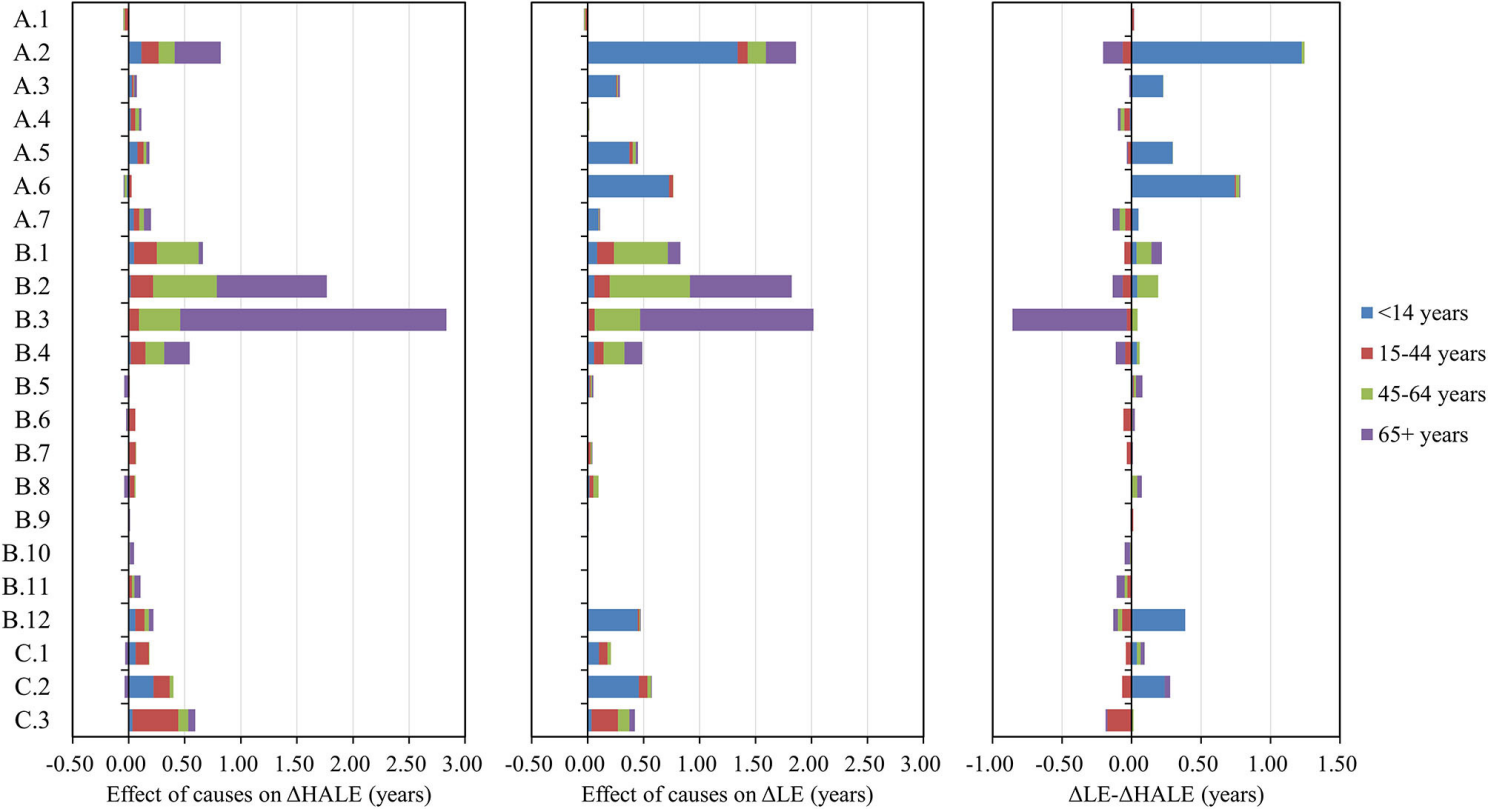
Cause-specific effects on changes in life expectancy (LE) and health-adjusted life expectancy (HALE) (levels 1–2) for different age groups from 1990 to 2019.

From 1990 to 2019, better control of NCDs in elderly aged more than 65 years old contributed most to the changes in HALE (3.68 years, 42.25%) (Figure 4). Better control of CMNNs in children aged under 14 years old had the greatest impact on LE (2.80 years, 26.69%). For injuries, more attention should be paid to older adults over 65 years of age, which had a smaller effect on changes in LE and a negative effect on changes in HALE. For children under 14 years old, LE improvements of more than 20% (2.53 years) were attributed to better control of the following 3 diseases: respiratory infections and tuberculosis (1.34 years), maternal and neonatal disorders (0.73 years), and unintentional injuries (0.46 years). But the total effect on improvement in HALE was very small (0.74 years, 8.50%). For youth aged 15–44 years, 10.10% (1.06 years) of improvement in LE and 21.47% (1.87 years) of improvement in HALE were attributed to all causes. For adults aged 45–64 years, the top level 2 cause with a positive effect on changes in LE and HALE was cardiovascular diseases (0.72 years, 6.86% and 0.57 years, 6.54%). Among older adults aged 65 years or older, the top 3 level 2 causes with a positive effect on changes in LE and HALE were chronic respiratory diseases (1.55 years, 14.78% and 2.37 years, 27.21%), cardiovascular diseases (0.91 years, 8.67% and 0.98 years, 11.25%), and respiratory infections and tuberculosis (0.27 years, 2.57% and 0.41 years, 4.71%). More details are summarized in Supplementary Table S7.

### Changes in LED

The LED, which refers to the difference between LE and HALE, increased by 1.78 years, mainly attributed to respiratory infections and tuberculosis (1.04 years, 58.43%) and maternal and neonatal disorders (0.78 years, 43.82%). The slow reduction in disability resulted in a longer LED. Additionally, LED increased by 1.27 years for males, through respiratory infections and tuberculosis (0.88 years, 69.29%) and maternal and neonatal disorders (0.82 years, 64.57%). LED increased by 2.51 years for females due to respiratory infections and tuberculosis (1.19 years, 47.41%) and maternal and neonatal disorders (0.76 years, 30.28%). Compared with the other level 2 causes and age groups, respiratory infections and tuberculosis (1.23 years, 69.10%) and maternal and neonatal disorders (0.74 years, 41.57%) in children aged <14 years contributed the most to increased LED, while chronic respiratory diseases in elderly aged more than 65 years old showed a negative effect (−0.82 years, −46.07%) on LED. For level 3 causes and both sexes, lower respiratory infections (1.06 years, 59.55%) and neonatal disorders (0.82 years, 46.07%,) contributed the most to increased LED. More details are summarized in Supplementary Tables S2, S6, S7.

## Discussion

This study was based on the largest epidemiological dataset to date in the 30-year history of GBD 2019 and continued to provide representative, comprehensive, and updated national estimates of the burden of diseases and injuries by integrating all available data. LE is a population health indicator of overall population health, and HALE is a more comprehensive indicator that incorporates mortality and disability into a single statistic (23). Arriaga's decomposition method was used to investigate sex-, age-, and cause-specific contributions in changes in LE and HALE across time in China, which is ideal for determining whether seemingly positive disparities are associated with large differences in cause-specific mortality or disability.

With improvement in the national economy and healthcare system, LE and HALE in China increased by 10.49 years and 8.71 years from 1990 to 2019, largely due to better control of NCDs (5.83 years, 55.58% for LE and 6.28 years, 72.10% for HALE) and, to a lesser extent, CMNNs (3.46 years, 32.98% for LE and 1.32 years, 15.15% for HALE) and injuries (1.20 years, 11.44% for LE and 1.11 years, 12.74% for HALE). According to the global research (24), NCDs were the main positive factor for HALE changes in high-income countries, while low-income countries experienced the most improvement from CMNNs. The results in China were more similar to those for high-income countries. Moreover, for level 3 causes, the top 10 positive factors leading to changes in LE were chronic obstructive pulmonary disease, lower respiratory infections, stroke, neonatal disorders, congenital birth defects, tuberculosis, liver cancer, self-harm, drowning, and stomach cancer; the top 10 positive factors leading to changes in HALE were chronic obstructive pulmonary disease, stroke, self-harm, tuberculosis, lower respiratory infections, hypertensive heart disease, liver cancer, stomach cancer, rheumatic heart disease, and drowning. According to previous studies (25–28), mortality from the above causes has decreased globally in the last decades, as it has in China, although they may have different effects on LE and HALE.

However, negative mortality and disability effects persist, especially in the period of 2010 to 2019. Both HIV/AIDS and sexually transmitted infections had negative mortality effects on changes in LE and HALE. According to GBD 2019, HIV/AIDS has become the leading cause of death in infectious diseases in China since 2009, with increasing mortality (29), as in other western countries (17). Theoretically, with the advent of antiretroviral treatment, the mortality rate would decrease significantly. However, those patients who did not meet the treatment criteria or had poor compliance may have higher mortality rates (30). For NCDs, level 3 causes of lung cancer, colon and rectum cancer, pancreatic cancer, and ischemic heart disease caused the main reduction in LE and HALE due to increased mortality, as has been proved in previous studies (31–35). For lung cancer, more emphasis should be placed on early screening, and tobacco control, and improving air pollution by reducing industrial energy consumption and dust (36, 37). For colon and rectum cancer, screening also has been shown to reduce their incidence and mortality, and in the longer term, primary prevention should be promoted (38). For pancreatic cancer, a timely and accurate diagnosis can improve the current poor prognosis of this disease (39). Improved dietary quality is associated with a reduced risk of pancreatic cancer (40). For ischemic heart disease, interventions should be expanded to control risk factors such as hypertension, hyperlipidemia, diabetes, and smoking, and eating more fresh fruit helps reduce the incidence of ischemic heart disease (41, 42). In addition, cardiovascular diseases (stroke), neonatal and neurological disorders, diabetes mellitus, and transport injuries (road injuries) had negative disability effects on changes in HALE as a result of increased disability rates. Consistent with previous studies (43–46), main causes such as stroke, diabetes mellitus, and road injuries are associated with an increased risk of disability. How well these diseases are rehabilitated is very important and more effective measures should be developed to decline the mortality of specific diseases, along with disability rates.

In addition, LEDs have increased in both sexes over the past three decades, which was mainly attributed to CMNNs, including lower respiratory infections and neonatal disorders. This is because the rate of mortality reduction has been much faster than the rate of disability reduction. The slow reduction in disability resulted in a longer LED. For most diseases and injuries, the mortality effect was dominant in influencing changes in HALE. It is noteworthy that the decline in mortality contributes more than 95% to the change of HALE in China, while disability reduction contributed <5%. HALE could not catch up with the growth rate of LE, which increased the additional personal, family, and social costs of medical services and long-term care for the elderly.

For different populations, more than 38% of improvement in LE was attributable to children under 14 years due to decreased mortality (47, 48). As the population ages, those over 65 years of age are coming to dominate the changes in LE and HALE. China is encountering formidable healthcare challenges brought about by the problem of aging (49, 50). Major diseases that need more attention in the elderly include cancer, cardiovascular diseases, diabetes, and injuries such as falls (13, 51). The increases in LE and HALE for females were higher than for males. More than 50% of improvement in LE and HALE for males and females was attributed to better control of the following 4 diseases: cardiovascular diseases, chronic respiratory diseases, respiratory infections and tuberculosis, and neoplasms. Similar to other studies worldwide (24), Chinese women had higher LED than men, along with higher incremental LED.

The rapid rise in NCDs driven by urbanization, income growth, and aging, as well as the transition to chronic disability, has brought major challenges to China's health system. Changes in cause-specific mortality and disability will require an integrated government response to improve primary healthcare and take necessary action to address key risks, especially for those diseases that are increasing with negative effects such as lung cancer, colon and rectum cancer, pancreatic cancer, stroke, ischemic heart disease, diabetes mellitus, and road injury. Analyses of changes and trend disparities in LE and HALE attributed to disability and mortality provide a useful framework to guide evidence-based policy responses to the changing disease spectrum in China, including timely adjustments to health programs and interventions. In the future, more studies should be implemented to investigate the drivers of heterogeneity observed in the changes and trend disparities in China, which may help guide more cost-effective and precise policies in China and other developing countries.

Nevertheless, there are several limitations in this study. First, the quality of mortality and disability data varied across years, which may affect the accuracy of the estimated changes in LE and HALE. We may overlook the cause-specific contributions between smaller time intervals. In the future, cause-specific contributions between each year will be explored. Second, data comparability over time should be considered. Different surveillance scope and completeness, diagnostic technology, and healthcare accessibility of diseases may affect the data quality. Third, this study shared the same limitations as the GBD 2019 study in the process of collecting and analyzing data on mortality and disability in China. Finally, this study only included levels 1–3 causes. Different diseases may have an opposite effect even in the same cause. More comprehensive studies will be required.

## Conclusion

The LE and HALE in China have grown rapidly over the past few decades, mainly attributed to NCDs. It is necessary to further reduce the negative mortality effect of HIV/AIDS, lung cancer, colon and rectum cancer, pancreatic cancer, and ischemic heart disease and the negative disability effect of stroke, diabetes mellitus, and road injuries. In addition, the signs of disparities in mortality and disability of different sexes and ages call for targeted and precise interventions for key groups such as males and the elderly. According to the decomposition results, we may better determine the key objects of health policies that take into account substantial cause-specific variations to facilitate the realization of “healthy China 2030” plan.

As a next step, to further improve LE and HALE, the construction of national demonstration areas for comprehensive prevention and control of NCDs should be expanded, including the national healthy lifestyle action and the project for early diagnosis and treatment of cancer. At the same time, we should not neglect to strengthen the prevention and control of HIV/AIDS and anti-virus treatment, as well as the rehabilitation after various diseases and injuries.

## Data Availability Statement

Publicly available datasets were analyzed in this study. This data can be found here: http://ghdx.healthdata.org/gbd-results-tool.

## Author Contributions

HC: conceptualization, visualization, and writing review and editing. LC: literature research, statistical analysis, and writing original draft preparation. YQ: methodology, data interpretation, and curation. LW: supervision and validation. All authors contributed to the article and approved the submitted version.

## Funding

This study was supported by Medical Key Discipline Program of Wuxi Health Commission (ZDXK010), Wuxi Tai Lake Talent Program, High-end Talent, Wuxi Eminent Medical Talents (ZDRC004), Wuxi Youth Medical Talents (QNRC050), Jiangsu Province Six-one Project for High-level Health Talents Research Fund (LGY2018014), Wuxi Precision Medicine Project (J202006), Top Talent Support Program for young and middle-aged people of Wuxi Health Committee (BJ2020096), and Youth Foundation of Wuxi Health Commission (Q202164).

## Conflict of Interest

The authors declare that the research was conducted in the absence of any commercial or financial relationships that could be construed as a potential conflict of interest.

## Publisher's Note

All claims expressed in this article are solely those of the authors and do not necessarily represent those of their affiliated organizations, or those of the publisher, the editors and the reviewers. Any product that may be evaluated in this article, or claim that may be made by its manufacturer, is not guaranteed or endorsed by the publisher.
