# Supplementary material for: Changes and Trend Disparities in Life Expectancy and Health-Adjusted Life Expectancy Attributed to Disability and Mortality From 1990 to 2019 in China
Source: Front Public Health. 2022 Jul 18;10:925114. doi: 10.3389/fpubh.2022.925114 (PMC9339800; doi:10.3389/fpubh.2022.925114)
Supplement: Supplementary file 1 [file Data_Sheet_1.pdf]

Table S1 | List of International Classification of Diseases (ICD) codes for causes from Global Burden of Disease 2019 (Level 1-3).

Level		Cause	ICD10	ICD9
2	A.1	HIV/AIDS and sexually transmitted infections	A50-A60.9, A63-A64.0, B20-B23.8, B24-B24.0, B63, B97.81, C46-C46.52, C46.7-C46.9, F02.4, I98.0, K67.0-K67.2, M73.0-M73.8, N70-N71.9, N73-N74, N74.2-N74.8, O98.7-O98.73, Z11.3-Z11.4, Z20.2, Z20.6, Z21, Z22.4, Z83.0	042-044.9, 054.1, 054.11-054.19, 090-099.9, 131-131.9, 176-176.9, 613-615.9, V01.6, V02.7-V02.9, V08, V73.8, V73.88, V73.9-V73.98, V74.5-V74.6
3	A.1.1	HIV/AIDS	B20-B23.8, B24-B24.0, B97.81, C46-C46.52, C46.7-C46.9, F02.4, O98.7-O98.73, Z11.4, Z20.6, Z21, Z83.0	042-044.9, 176-176.9, V08
3	A.1.2	Sexually transmitted infections excluding HIV	A50-A60.9, A63-A64.0, B63, I98.0, K67.0-K67.2, M73.0-M73.8, N70-N71.9, N73-N74, N74.2-N74.8, Z11.3, Z20.2, Z22.4	054.1, 054.11-054.19, 090-099.9, 131-131.9, 613-615.9, V01.6, V02.7-V02.9, V73.8, V73.88, V73.9-V73.98, V74.5-V74.6
2	A.2	Respiratory infections and tuberculosis	A10-A14, A15-A18.89, A19-A19.9, A48.1, A70, B90-B90.9, B96.0-B96.1, B97.21, B97.4-B97.6, H65-H70.93, J00-J06.9, J09-J18.2, J18.8-J18.9, J19.6-J22.9, J36-J36.0, J85.1, J91.0, K67.3, K93.0, M49.0, N74.0-N74.1, P23-P23.9, P37.0, U04 -U04.9, U84.3, Z03.0, Z11.1, Z20.1, Z23.2, Z25.1	010-019.9, 079.82, 137-137.9, 320.4, 381-383.9, 460-469, 470.0, 475-475.9, 480-484, 484.1-490.9, 510-511.9, 513.0-513.9, 730.4-730.6, 770.0, V01.1, V01.82, V03.2, V04.7, V04.81, V12.01, V12.61, V74.1
3	A.2.1	Tuberculosis	A10-A14, A15-A18.89, A19-A19.9, B90-B90.9, K67.3, K93.0, M49.0, N74.0-N74.1, P37.0, U84.3, Z03.0, Z11.1, Z20.1, Z23.2	010-019.9, 137-137.9, 320.4, 730.4-730.6, V01.1, V03.2, V12.01, V74.1
3	A.2.2	Lower respiratory infections	A48.1, A70, B96.0-B96.1, B97.21, B97.4-B97.6, J09-J18.2, J18.8-J18.9, J19.6-J22.9, J85.1, J91.0, P23-P23.9, U04 -U04.9, Z25.1	079.82, 466-469, 470.0, 480-484, 484.1-490.9, 510-511.9, 513.0-513.9, 770.0, V01.82, V04.7, V04.81, V12.61
3	A.2.3	Upper respiratory infections	J00-J06.9, J36-J36.0	460-465.9, 475-475.9
3	A.2.4	Otitis media	H65-H70.93	381-383.9
2	A.3	Enteric infections	A00-A08.8, A09, A80-A80.9, B91, K52.1, Z11.0, Z20.0-Z20.09, Z22.1, Z23.0	001-009.9, 045-045.93, 138, V01.0, V01.83, V02.0, V02.2-V02.3, V03.0, V74.0
3	A.3.1	Diarrheal diseases	A00-A00.9, A02-A02.0, A02.8-A07, A07.2-A07.4, A08-A08.8, A09, K52.1, Z22.1, Z23.0	001-001.9, 003.8-009.9, V01.0, V01.83, V02.0, V02.2-V02.3, V03.0, V74.0
3	A.3.2	Typhoid and paratyphoid	A01-A01.4, Z11.0, Z20.0-Z20.09	002-002.9
3	A.3.3	Invasive Non-typhoidal Salmonella (iNTS)	A02.1-A02.29	003-003.7
3	A.3.4	Other intestinal infectious diseases	A07.0-A07.1, A07.8-A07.9, A80-A80.9, B91	045-045.93, 138
2	A.4	Neglected tropical diseases and malaria	A30-A30.9, A68-A68.9, A69.2-A69.29, A69.8-A69.9, A71-A71.9, A74.0, A75-A75.9, A77-A79.9, A82-A82.9, A90-A91.0, A92-A96.9, A98-A99.0, B33.0-B33.1, B50-B50.0,	030-030.9, 060-061.8, 065-066.9, 071-071.9, 076-076.9, 080-088.9, 120-129.0, 425.6, V01.5,

Level		Cause	ICD10	ICD9
			B50.8-B52.0, B52.8-B53.1, B53.8-B57.5, B60-B60.8, B64-B83.9, B89, B92, B94.0, K93.1, P37.1, P37.3-P37.4, U06-U06.9, Z11.6, Z20.3, Z24.2-Z24.3, Z26.0	V04.4-V04.5, V05.2, V12.03, V73.4-V73.6, V74.2, V75.1-V75.3, V75.5-V75.8
3	A.4.1	Malaria	B50-B50.0, B50.8-B52.0, B52.8-B53.1, B53.8-B54.0, P37.3-P37.4	084-084.9, V12.03, V75.1
3	A.4.2	Chagas disease	B57-B57.5, K93.1	086-086.2, 425.6
3	A.4.3	Leishmaniasis	B55-B55.9, Z26.0	085-085.9, V05.2, V75.2
3	A.4.4	African trypanosomiasis	B56-B56.9	086.3-086.9, V75.3
3	A.4.5	Schistosomiasis	B65-B65.9	120-120.9, V75.5
3	A.4.6	Cysticercosis	B69-B69.9	123.1
3	A.4.7	Cystic echinococcosis	B67-B67.4, B67.8-B67.99	122-122.4, 122.8-122.9
3	A.4.8	Lymphatic filariasis	B74-B74.2	125.0-125.2
3	A.4.9	Onchocerciasis	B73-B73.1	125.3
3	A.4.10	Trachoma	A71-A71.9, A74.0, B94.0	076-076.9, V73.6
3	A.4.11	Dengue	A90-A91.0	061-061.8
3	A.4.12	Yellow fever	A95-A95.9, Z24.3	060-060.9, V04.4, V73.4
3	A.4.13	Rabies	A82-A82.9, Z20.3, Z24.2	071-071.9, V01.5, V04.5
3	A.4.14	Intestinal nematode infections	B76-B77.9, B79, Z11.6	126-126.9, 127.0, 127.3, V75.7
3	A.4.15	Food-borne trematodiases	B66-B66.9, B72.0	121-121.9, V75.6
3	A.4.16	Leprosy	A30-A30.9, B92	030-030.9, V74.2
3	A.4.17	Ebola	A98.4	
3	A.4.18	Zika virus	U06-U06.9	066.3
3	A.4.19	Guinea worm disease	B72	
3	A.4.20	Other neglected tropical diseases	A68-A68.9, A69.2-A69.29, A69.8-A69.9, A75-A75.9, A77-A79.9, A92-A94.0, A96-A96.9, A98-A98.3, A98.5-A99.0, B33.0-B33.1, B60-B60.8, B64, B67.5-B67.7, B68-B68.9, B70-B71.9, B74.3-B75, B78-B78.9, B80-B83.9, B89, P37.1	065-066.2, 066.4-066.9, 080-083.9, 087-088.9, 122.5-122.7, 123-123.0, 123.2-125, 125.4-125.9, 127, 127.1-127.2, 127.4-129.0, V73.5, V75.8
2	A.5	Other infectious diseases	A20-A28.9, A31-A39.9, A42-A44.9, A48-A48.0, A48.2-A49.9, A65-A65.0, A69-A69.1, A74, A74.8-A74.9, A81-A81.9, A83-A85.2, A85.8-A86.0, A87-A89.0, B00-B06.9, B10-B10.89, B15-B19.9, B25-B27.99, B33, B33.3-B34.9, B37-B37.2, B37.5-B47.1, B47.9-B49, B58-B59, B94, B94.1-B95.0, B95.2-B96, B96.2-B97.2, B97.29-B97.39, B97.7-B97.8,	020-029, 031-034.9, 036-039.4, 039.8-040, 040.1-040.9, 046-054.0, 054.10, 054.2-059.9, 062-064.9, 070-070.9, 072-074.1, 074.20, 074.3-075.9, 078, 078.2-079.81, 079.83-079.99, 100-101.6, 104-104.9, 112-112.0, 112.3-118.9, 130-130.9, 136-136.0, 136.2-136.9, 138.0-139.9,

Level		Cause	ICD10	ICD9
			B97.89, B99-B99.9, D70.3, D86.81, D89.3, F02.1, F07.1, G00-G09.9, G14-G14.6, I00, I02, I02.9, I96-I96.9, I98.1, J85-J85.0, J85.2-J85.3, J86-J86.9, K75.0, K75.3, K76.3, M49.1, M89.6-M89.69, P35-P35.9, P37, P37.2, P37.5-P37.9, R02-R02.9, U82-U84, U85-U89, Z11, Z11.2, Z11.5-Z11.59, Z11.8-Z11.9, Z16-Z16.39, Z20, Z20.4-Z20.5, Z20.8-Z20.9, Z22-Z22.0, Z22.2-Z22.39, Z22.5-Z22.9, Z23.3-Z23.7, Z24.0-Z24.1, Z24.4-Z25.0, Z83.1	310.89, 320-320.3, 320.5-326.9, 390-390.9, 392, 392.9, 484.0, 572.0-572.1, 771.0-771.3, V01, V01.2-V01.4, V01.7-V01.81, V01.84-V02, V02.1, V02.4-V02.5, V02.52-V02.69, V03.1, V03.3-V04.3, V04.6, V04.8, V04.89-V05.1, V05.3-V06, V07-V07.0, V07.2-V07.3, V09-V09.91, V12.00, V12.02, V12.04-V12.09, V18.8, V58.62, V73.0-V73.3, V73.81, V73.89, V73.99, V74.3, V74.8-V74.9, V75.0, V75.4, V75.9
3	A.5.1	Meningitis	A39-A39.9, A87-A87.9, D86.81, G00-G03.9, G06-G09.9, Z20.811, Z22.31	036-036.9, 047-049.9, 054.72, 320-320.3, 320.5-322.9, 324-326.9, V01.84
3	A.5.2	Encephalitis	A83-A85.2, A85.8-A86.0, B94.1, F07.1, G04-G05.8, Z24.1	062-064.9, 310.89, 323-323.9, V05.0-V05.1
3	A.5.3	Diphtheria	A36-A36.9, Z22.2, Z23.6	032-032.9, V02.4, V03.5, V74.3
3	A.5.4	Whooping cough	A37-A37.91, Z23.7	033-033.9, V03.6
3	A.5.5	Tetanus	A33-A35.0, Z23.5	037-037.9, 771.3, V03.7
3	A.5.6	Measles	B05-B05.9, Z24.4	055-055.9, 484.0, V04.2, V73.2
3	A.5.7	Varicella and herpes zoster	B01-B02.9, Z20.820	052-053.9, V01.71-V01.79, V05.4
3	A.5.8	Acute hepatitis	B15-B19.9, B94.2, P35.3, Z20.5, Z22.5-Z22.59, Z24.6	070-070.9, V02.6-V02.69, V05.3
3	A.5.9	Other unspecified infectious diseases	A20-A28.9, A31-A32.9, A38-A38.9, A42-A44.9, A48-A48.0, A48.2-A49.9, A65-A65.0, A69-A69.1, A74, A74.8-A74.9, A81-A81.9, A88-A89.0, B00-B00.9, B03-B04, B06-B06.9, B10-B10.89, B25-B27.99, B33, B33.3-B34.9, B37-B37.2, B37.5-B47.1, B47.9-B49, B58-B59, B94, B94.8-B95.0, B95.2-B96, B96.2-B97.2, B97.29-B97.39, B97.7-B97.8, B97.89, B99-B99.9, D70.3, D89.3, F02.1, G14-G14.6, I00, I02, I02.9, I96-I96.9, I98.1, J85-J85.0, J85.2-J85.3, J86-J86.9, K75.0, K75.3, K76.3, M49.1, M89.6-M89.69, P35-P35.2, P35.8-P35.9, P37, P37.2, P37.5-P37.9, R02-R02.9, U82-U84, U85-U89, Z11, Z11.2, Z11.5-Z11.59, Z11.8-Z11.9, Z16-Z16.39, Z20, Z20.4, Z20.8-Z20.810, Z20.818-Z20.82, Z20.828-Z20.9, Z22-Z22.0, Z22.3, Z22.32-Z22.39, Z22.6-Z22.9, Z23.3-Z23.4, Z24.0, Z24.5, Z25.0, Z83.1	020-029, 031-031.9, 034-034.9, 039-039.4, 039.8-040, 040.1-040.9, 046-046.9, 050-051.9, 054-054.0, 054.10, 054.2-054.71, 054.73-054.9, 056-059.9, 072-074.1, 074.20, 074.3-075.9, 078, 078.2-079.81, 079.83-079.99, 100-101.6, 104-104.9, 112-112.0, 112.3-118.9, 130-130.9, 136-136.0, 136.2-136.9, 138.0-139.9, 390-390.9, 392, 392.9, 572.0-572.1, 771.0-771.2, V01, V01.2-V01.4, V01.7, V01.8-V01.81, V01.89-V02, V02.1, V02.5, V02.52-V02.59, V03.1, V03.3-V03.4, V03.8-V04.1, V04.3, V04.6, V04.8, V04.89-V05, V05.8-V06, V07-V07.0, V07.2-V07.3, V09-V09.91, V12.00, V12.02, V12.04-V12.09, V18.8, V58.62, V73.0-V73.1, V73.3, V73.81, V73.89, V73.99, V74.8-V74.9, V75.0, V75.4, V75.9

Level		Cause	ICD10	ICD9
2	A.6	Maternal and neonatal disorders	A40.1, B95.1, F53-F54, N82-N82.9, N96, O00-O16.9, O18.0, O20-O26.93, O28-O36.93, O38.4, O40-O48.1, O60-O77.9, O80-O92.79, O94-O95, O96-O98.63, O98.8-P05.9, P07-P15.9, P19-P22.9, P24-P29.9, P36-P36.9, P38-P39.9, P50-P61.9, P70-P72.9, P74-P74.9, P75.0-P78.9, P80-P81.9, P83-P84, P90-P92.9, P94-P94.9, P96, P96.3-P96.4, P96.8-P96.9, P99.9, Z03.7-Z03.79, Z32-Z37.9, Z39-Z3A49, Z64.0-Z64.3, Z87.5-Z87.6	041.02, 619-619.9, 630-679.14, 760-768, 768.2-770, 770.2-771, 771.4-775.1, 775.4-779.34, 779.7-779.9, V02.51, V13.1, V13.21, V13.7, V15.21-V15.22, V15.87, V22-V24.2, V27-V28.9, V72.4-V72.42, V82.4, V91-V91.99
3	A.6.1	Maternal disorders	F53-F54, N82-N82.9, N96, O00-O16.9, O18.0, O20-O26.93, O28-O36.93, O38.4, O40-O48.1, O60-O77.9, O80-O92.79, O94-O95, O96-O98.63, O98.8-O9A513, Z03.7-Z03.79, Z32-Z37.9, Z39-Z3A49, Z64.0-Z64.3, Z87.5-Z87.6	619-619.9, 630-655.23, 655.7-679.14, V13.1, V13.21, V15.21-V15.22, V22-V24.2, V27-V28.9, V72.4-V72.42, V82.4, V91-V91.99
3	A.6.2	Neonatal disorders	A40.1, B95.1, P00-P05.9, P07-P15.9, P19-P22.9, P24-P29.9, P36-P36.9, P38-P39.9, P50-P61.9, P70-P72.9, P74-P74.9, P75.0-P78.9, P80-P81.9, P83-P84, P90-P92.9, P94-P94.9, P96, P96.3-P96.4, P96.8-P96.9, P99.9	041.02, 655.3-655.63, 760-768, 768.2-770, 770.2-771, 771.4-775.1, 775.4-779.34, 779.7-779.9, V02.51, V13.7, V15.87
2	A.7	Nutritional deficiencies	D50-D53.9, E00-E02, E40-E46.9, E50-E61.9, E63-E64.9, Z13.2-Z13.3	244.2, 260-269.9, 280-281.2, V12.1, V18.2-V18.3, V77.2, V78.0-V78.1
3	A.7.1	Protein-energy malnutrition	E40-E46.9, E64.0	260-263.9
3	A.7.2	Iodine deficiency	E00-E02	244.2
3	A.7.3	Vitamin A deficiency	E50-E50.9, E64.1	264-264.9
3	A.7.4	Dietary iron deficiency	D50-D50.9	280-280.9
3	A.7.5	Other nutritional deficiencies	D51-D53.9, E51-E61.9, E63-E64, E64.2-E64.9	265-269.9, 281-281.2
2	B.1	Neoplasms	C00-C07, C08-C19.0, C20, C21-C21.8, C22-C22.4, C22.7-C23, C24-C26.1, C26.8-C26.9, C30-C30.1, C31-C33, C34-C34.92, C37-C37.0, C38-C39.9, C40-C41.4, C41.8-C41.9, C43-C45.2, C45.7, C45.9, C47-C4A, C50-C50.629, C50.8-C52, C53-C54.3, C54.8-C56.2, C56.9-C58.0, C60-C64.2, C64.9-C69.92, C70-C70.1, C70.9-C73, C74-C75.5, C75.8-C79.9, C80-C81.49, C81.7-C81.79, C81.9-C85.29, C85.7-C86.6, C88-C90.32, C91-C93.7, C93.9-C95.2, C95.7-C97.9, D00-D24.9, D26.0-D39.9, D4-D49.9, E34.0, K51.4-K51.419, K62.0-K62.3, K63.5, N60-N60.99, N84.0-N84.1, N87-N87.9, Z03.1, Z08-Z09.9, Z12-Z12.9, Z80-Z80.9, Z85-Z85.9, Z86.0-Z86.03	140-175.9, 177-217.8, 219-237.6, 237.70-237.72, 237.9-239.9, 569.0, 610-610.9, 622.1-622.2, 622.7, V07.39, V10-V11, V13.22-V13.24, V16-V16.9, V42.4, V42.81-V42.82, V59.2-V59.3, V72.32, V76-V76.9

Level		Cause	ICD10	ICD9
3	B.1.1	Lip and oral cavity cancer	C00-C07, C08-C08.9, Z85.81-Z85.810	140-145.9, V76.42
3	B.1.2	Nasopharynx cancer	C11-C11.9	147-147.9
3	B.1.3	Other pharynx cancer	C09-C10.9, C12-C13.9	146-146.9, 148-148.9
3	B.1.4	Esophageal cancer	C15-C15.9, Z85.01	150-150.9
3	B.1.5	Stomach cancer	C16-C16.9, Z12.0, Z85.02-Z85.028	151-151.9, 209.23, V10.04
3	B.1.6	Colon and rectum cancer	C18-C19.0, C20, C21-C21.8, Z12.1-Z12.13, Z85.03-Z85.048, Z86.010	153-154.9, 209.1-209.17, V10.05-V10.06, V76.41, V76.5-V76.52
3	B.1.7	Liver cancer	C22-C22.4, C22.7-C22.9, Z85.05	155-155.9, V10.07
3	B.1.8	Gallbladder and biliary tract cancer	C23, C24-C24.9	156-156.9
3	B.1.9	Pancreatic cancer	C25-C25.9, Z85.07	157-157.9
3	B.1.10	Larynx cancer	C32-C32.9, Z85.21	161-161.9, V10.21
3	B.1.11	Tracheal, bronchus, and lung cancer	C33, C34-C34.92, Z12.2, Z80.1-Z80.2, Z85.1-Z85.20	162-162.9, 209.21, V10.1-V10.20, V16.1-V16.2, V16.4-V16.40
3	B.1.12	Malignant skin melanoma	C43-C43.9, Z85.82-Z85.828	172-172.9
3	B.1.13	Non-melanoma skin cancer	C44.01-C44.99	173-173.99
3	B.1.14	Breast cancer	C50-C50.629, C50.8-C50.929, Z12.3-Z12.39, Z80.3, Z85.3, Z86.000	174-175.9, V10.3, V16.3
3	B.1.15	Cervical cancer	C53-C53.9, Z12.4, Z85.41	180-180.9, V10.41, V72.32
3	B.1.16	Uterine cancer	C54-C54.3, C54.8-C54.9, Z85.42, Z86.001	182-182.9
3	B.1.17	Ovarian cancer	C56-C56.2, C56.9, Z80.41, Z85.43	183-183.0, 183.8-183.9, V10.43, V16.41
3	B.1.18	Prostate cancer	C61-C61.9, Z12.5, Z80.42, Z85.46	185-185.9, V10.46, V16.42, V76.44
3	B.1.19	Testicular cancer	C62-C62.92, Z80.43, Z85.47-Z85.48	186-186.9, V10.47-V10.48, V16.43
3	B.1.20	Kidney cancer	C64-C64.2, C64.9-C65.9, Z80.51, Z85.52-Z85.54	189-189.1, 189.5-189.6, 209.24
3	B.1.21	Bladder cancer	C67-C67.9, Z12.6-Z12.79, Z80.52, Z85.51	188-188.9, V10.51, V16.52, V76.3
3	B.1.22	Brain and central nervous system cancer	C70-C70.1, C70.9-C72.9, Z85.841-Z85.848, Z86.011	191-191.9
3	B.1.23	Thyroid cancer	C73, Z85.850	193-193.9
3	B.1.24	Mesothelioma	C45-C45.2, C45.7, C45.9	
3	B.1.25	Hodgkin lymphoma	C81-C81.49, C81.7-C81.79, C81.9-C81.99, Z85.71-Z85.72	201-201.98, V10.72
3	B.1.26	Non-Hodgkin lymphoma	C82-C85.29, C85.7-C86.6, C96-C96.9	200-200.9, 202-202.98
3	B.1.27	Multiple myeloma	C88-C90.32	203-203.9

Level		Cause	ICD10	ICD9
3	B.1.28	Leukemia	C91-C93.7, C93.9-C95.2, C95.7-C95.92, Z80.6, Z85.6	204-208.92, V10.59-V10.69, V16.6
3	B.1.30	Other neoplasms	C75.90-C75.92, D00-D24.9, D26.0-D39.9, D4-D49.9, E34.0, K51.4-K51.419, K62.0-K62.3, K63.5, N60-N60.99, N84.0-N84.1, N87-N87.9	209.4-209.57, 209.61, 209.63-209.67, 210.0-217.8, 219-237.6, 237.70-237.72, 237.9-239.9, 569.0, 610-610.9, 622.1-622.2, 622.7
2	B.2	Cardiovascular diseases	B33.2-B33.24, D86.85, G45-G46.8, I01-I01.9, I02.0, I05-I09.9, I11-I11.2, I11.9, I20-I21.6, I21.9-I27.0, I27.2-I28.9, I30-I38.0, I39-I41.8, I42-I43.8, I44-I44.8, I45-I52.8, I60-I64, I64.1, I65-I83.93, I86-I89.0, I89.9, I95.0-I95.1, I98, I98.8-I99.9, K75.1, R00-R01.2, Z01.3-Z01.31, Z03.4-Z03.5, Z13.6, Z52.7, Z82.3-Z82.49, Z86.7-Z86.79, Z94.1-Z94.3, Z95-Z95.9	074.2, 074.21-074.23, 391-391.9, 392.0, 393-398.99, 402-402.91, 410-416.0, 416.8-417.9, 420-425.5, 425.7-440.29, 440.4-445.89, 447-454.9, 456, 456.3-457, 457.1, 457.8-458.1, 459-459.9, 785-785.3, V12.5-V12.59, V15.1, V17.1, V17.3-V17.49, V42.1-V42.2, V43.2-V43.5, V45.0-V45.09, V45.81-V45.82, V47.2, V58.61, V58.63, V58.66, V58.73, V81-V81.2
3	B.2.1	Rheumatic heart disease	I01-I01.9, I02.0, I05-I09.9	391-391.9, 392.0, 393-398.99
3	B.2.2	Ischemic heart disease	I20-I21.6, I21.9-I25.9, Z82.4-Z82.49	410-414.9, V17.3
3	B.2.3	Stroke	G45-G46.8, I60-I62, I62.9-I64, I64.1, I65-I69.998, Z82.3	430-439.6, V12.54, V17.1
3	B.2.4	Hypertensive heart disease	I11-I11.2, I11.9	402-402.91
3	B.2.5	Non-rheumatic valvular heart disease	I34-I37.9	424.0-424.3
3	B.2.6	Cardiomyopathy and myocarditis	B33.2-B33.20, B33.22-B33.24, D86.85, I40-I41.8, I42-I43.8, I51.4-I51.6	074.2, 074.23, 422-422.99, 425-425.5, 425.7-425.9, 429.0-429.1
3	B.2.8	Atrial fibrillation and flutter	I48-I48.92	427.3-427.32
3	B.2.10	Peripheral artery disease	I70.2-I70.92, I73-I73.9	440.2-440.29, 440.4-440.9, 443-443.2, 443.8-443.9
3	B.2.11	Endocarditis	B33.21, I33-I33.9, I38-I38.0, I39-I39.9	074.22, 421-421.9, 424, 424.4-424.99
2	B.3	Chronic respiratory diseases	D86-D86.2, D86.9, G47.3-G47.39, J30-J35.9, J37-J39.9, J41-J42.4, J43-J46.0, J47-J47.9, J60-J68.9, J70.8-J70.9, J80-J80.9, J82, J84-J84.9, J90-J90.0, J91, J91.8-J93.12, J93.8-J94.9, J96-J96.92, J98-J99.8, R05.0-R06.9, R09-R09.89, R84-R84.9, R91-R91.8, Z82.5	135-135.9, 278.03, 327.2-327.29, 470, 470.9-474.9, 476-479, 491-508.9, 512-513, 514-518.53, 518.8-519, 519.11-519.9, 786-786.9, 793.1-793.2, 799.0-799.1, V07.1, V12.6-V12.60, V12.69, V13.81, V14-V15.09, V15.84, V17.5-V17.6, V19.6, V42.6, V43.81, V45.76, V58.74, V81.3-V81.4
3	B.3.1	Chronic obstructive pulmonary disease	J41-J42.4, J43-J44.9	491-492.9, 496-499
3	B.3.2	Pneumoconiosis	J60-J65.0, J92.0	500-505.9
3	B.3.3	Asthma	J45-J46.0, Z82.5	493-493.92, V17.5

Level		Cause	ICD10	ICD9
3	B.3.4	Interstitial lung disease and pulmonary sarcoidosis	D86-D86.2, D86.9, J84-J84.9	135-135.9, 515, 515.9-516.9
3	B.3.5	Other chronic respiratory diseases	J30-J35.9, J37-J39.9, J47-J47.9, J66-J68.9, J70.8-J70.9, J82, J90-J90.0, J91, J91.8-J92, J92.9-J93.12, J93.8-J94.9, J96.1-J96.8, J98-J99.8	470, 470.9-474.9, 476-479, 494-495.9, 506-508.9, 512-513, 514-514.9, 515.0, 517-518.4, 518.8-518.81, 518.83-519, 519.11-519.9, V07.1, V12.6-V12.60, V12.69, V13.81, V14-V15.09, V15.84, V17.6, V19.6, V42.6, V43.81, V45.76, V58.74, V81.3-V81.4
2	B.4	Digestive diseases	I84-I85.9, I98.2, K20-K23.8, K25-K31.9, K35-K38.9, K40-K42.9, K44-K46.9, K50-K51.319, K51.5-K52, K52.2-K52.9, K55-K62, K62.4-K62.6, K62.8-K63.4, K63.8-K67, K67.8-K68.1, K68.12-K68.9, K70-K75, K75.2, K75.4-K76.2, K76.4-K77.8, K80-K80.81, K81-K83.9, K85-K87.1, K90-K90.9, K92-K92.9, K93.8, R11-R19.8, R85-R85.9, Z13.81-Z13.818, Z43.1-Z43.4, Z52.6, Z83.7-Z83.79, Z87.1-Z87.19, Z94.4	455-455.9, 456.0-456.21, 530-530.85, 530.89-536.3, 536.8-538, 540-543.9, 550-551.1, 551.3-552.1, 552.3-553.1, 553.3-558.9, 560-560.39, 560.8-562.13, 564-564.1, 564.5-569, 569.1-569.5, 569.81-572, 572.2-579.2, 579.4-579.9, 784-784.99, 787-787.99, 789.9, 792.1-792.4, V12.7-V12.79, V18.5-V18.59, V42.7, V45.72, V45.75, V47.3, V58.75, V59.6
3	B.4.1	Cirrhosis and other chronic liver diseases	I85-I85.9, I98.2, K70-K71, K71.3-K72, K72.1-K75, K75.2, K75.4-K76.2, K76.4-K77.8, R16-R18.9, Z52.6, Z94.4	456.0-456.21, 570-572, 572.2-573.9, V42.7, V59.6
3	B.4.2	Upper digestive system diseases	K21-K21.9, K22.7-K22.719, K25-K30, R12	530.11, 530.7-530.85, 530.89-536.3, 536.8-536.9, 787.1
3	B.4.3	Appendicitis	K35-K37.9	540-542.9
3	B.4.4	Paralytic ileus and intestinal obstruction	K56-K56.9	560-560.39, 560.8-560.9, 569.87
3	B.4.5	Inguinal, femoral, and abdominal hernia	K40-K42.9, K44-K46.9	550-551.1, 551.3-552.1, 552.3-553.1, 553.3-553.9
3	B.4.6	Inflammatory bowel disease	K50-K51.319, K51.5-K52, K52.8-K52.9	555-556.9, 558-558.9, 564.1, 569.5
3	B.4.7	Vascular intestinal disorders	K55-K55.9	557-557.9, 569.84-569.86
3	B.4.8	Gallbladder and biliary diseases	K80-K80.81, K81-K83.9, K87-K87.1	574-576.9
3	B.4.9	Pancreatitis	K85-K86.9	577-577.9
3	B.4.10	Other digestive diseases	I84-I84.9, K20-K20.9, K22-K22.6, K22.8-K23.8, K31-K31.9, K38-K38.9, K52.2-K52.3, K57-K62, K62.4-K62.6, K62.8-K63.4, K63.8-K67, K67.8-K68.1, K68.12-K68.9, K71.0-K71.2, K72.0-K72.01, K90-K90.9, K92-K92.9, K93.8	455-455.9, 530-530.10, 530.12-530.6, 537-538, 543-543.9, 561-562.13, 564-564.09, 564.5-569, 569.1-569.49, 569.81-569.83, 569.89-569.9, 578-579.2, 579.4-579.9
2	B.5	Neurological disorders	F00-F02.0, F02.2-F02.3, F02.8-F03.91, F06.2, G10-G10.0, G11-G13.8, G20-G21, G21.2-G24, G24.1-G25.0, G25.2-	290-290.9, 294.0-294.9, 307.8-307.89, 315-315.9, 330-331.8, 331.82-333.91, 333.93-346.93,

Level		Cause	ICD10	ICD9
			G25.3, G25.5, G25.8-G26.0, G30-G31.1, G31.8-G32.89, G35-G35.0, G36-G37.9, G40-G41.9, G43-G44.89, G50-G54.1, G54.5-G62, G62.2-G65.2, G70-G71.19, G71.3-G72, G72.1-G73.7, G80-G83.9, G89-G93.6, G93.8-G95.29, G95.8-G96, G96.1, G96.12-G96.9, G98-G99.8, M33-M33.99, M60-M60.19, M60.8-M60.9, M79.7, R25-R27.9, R29-R29.91, R41-R42.0, R56-R56.9, R90-R90.89, Z03.3, Z13.85, Z13.858, Z82.0, Z86.6-Z86.69	348-348.9, 350-353.0, 353.5-357.5, 357.7-359.23, 359.29-359.9, 710.3-710.4, 725-725.9, 728-728.85, 728.87-728.9, 775.2, 780.3-780.59, 780.7-780.72, 780.96, 781-781.99, 793.0, 799.3-799.7, V17.2, V58.72
3	B.5.1	Alzheimer's disease and other dementias	F00-F02.0, F02.8-F03.91, F06.2, G30-G31.1, G31.8-G32.89	290-290.9, 294.0-294.9, 331-331.2, 331.6-331.7, 331.82, 331.89-331.9
3	B.5.2	Parkinson's disease	F02.3, G20-G20.9	332-332.0
3	B.5.3	Idiopathic epilepsy	G40-G41.9, Z82.0	345-345.91
3	B.5.4	Multiple sclerosis	G35-G35.0	340-340.9
3	B.5.6	Headache disorders	G43-G44.89	307.81, 339-339.89, 346-346.93
3	B.5.7	Other neurological disorders	F02.2, G10-G10.0, G11-G13.8, G21, G21.2-G24, G24.1-G25.0, G25.2-G25.3, G25.5, G25.8-G26.0, G36-G37.9, G50-G54.1, G54.5-G62, G62.2-G65.2, G70-G71.19, G71.3-G72, G72.1-G73.7, G80-G83.9, G89-G93.6, G93.8-G95.29, G95.8-G96, G96.1, G96.12-G96.9, G98-G99.8, M33-M33.99, M60-M60.19, M60.8-M60.9, M79.7	307.8-307.80, 307.89, 330-330.9, 331.3-331.5, 331.8, 331.83, 332.1-333.91, 333.93-338.4, 341-344.9, 348-348.9, 350-353.0, 353.5-357.5, 357.7-359.23, 359.29-359.9, 710.3-710.4, 725-725.9, 728-728.85, 728.87-728.9, 775.2, 780.96
2	B.6	Mental disorders	F04-F06.1, F06.3-F07.0, F08-F09.9, F20-F34.9, F38-F52.9, F55-F55.8, F56-F99.0, G47-G47.29, G47.4-G47.9, R40-R40.4, R45-R46.89, R55-R55.0, Z03.2, Z04.6-Z04.72, Z13.4, Z64, Z81-Z81.0, Z81.8, Z86.5-Z86.59	293-294, 295-302.9, 306-307.7, 307.9-310.1, 311-314.9, 316-319.9, 327-327.19, 327.3-327.8, 347-347.9, 780-780.2, 780.93, 780.97, 797-797.9, 799.2-799.29, V11.0-V11.2, V11.4-V12.0, V17-V17.0, V18.4, V40-V41.9, V79-V79.9
3	B.6.1	Schizophrenia	F20-F20.9, F25-F25.9	295-295.35, 295.5-295.8
3	B.6.2	Depressive disorders	F32-F33.9, F34.1	296.2-296.36, 300.4, 311-311.9
3	B.6.3	Bipolar disorder	F30-F31.9, F34.0	296-296.16, 296.4-296.81
3	B.6.4	Anxiety disorders	F40-F44.9, F93-F93.2	300-300.3, 308-309.9
3	B.6.5	Eating disorders	F50-F50.9	307.1, 307.5-307.59
3	B.6.7	Attention-deficit/hyperactivity disorder	F90-F90.9	314-314.9
3	B.6.8	Conduct disorder	F91-F92.9	312-312.9

Level		Cause	ICD10	ICD9
3	B.6.9	Idiopathic developmental intellectual disability	F70-F79.9, Z81.0	317-319.9, V18.4
3	B.6.10	Other mental disorders	F04-F06.1, F06.3-F07.0, F08-F09.9, F21-F24, F26-F29.9, F34, F34.8-F34.9, F38-F39, F45-F49, F51-F52.9, F55-F55.8, F56-F69.0, F80-F89.0, F93.3-F99.0, G47-G47.29, G47.4-G47.9, R40-R40.4, R45-R46.89, R55-R55.0, Z03.2, Z04.6-Z04.72, Z13.4, Z64, Z81, Z81.8, Z86.5-Z86.59	293-294, 295.4-295.45, 295.80-295.95, 296.82-298.9, 300.5-302.9, 306-307.0, 307.2-307.49, 307.6-307.7, 307.9, 310-310.1, 313-313.9, 316-316.9, 327-327.19, 327.3-327.8, 347-347.9, 780-780.2, 780.93, 780.97, 797-797.9, 799.2-799.29, V11.0-V11.2, V11.4-V12.0, V17-V17.0, V40-V41.9, V79-V79.9
2	B.7	Substance use disorders	E24.4, F10-F19.99, G31.2, G62.1, P96.1, R78.0-R78.9, X45-X45.9, X65-X65.9, Y15-Y15.9, Z81.1-Z81.4	291-292.9, 303-305.93, 790.3, E850.0-E850.29, E860-E860.19, V11.3, V15.8-V15.83, V15.85-V15.86, nan
3	B.7.1	Alcohol use disorders	E24.4, F10-F10.99, G31.2, G62.1, R78.0, X45-X45.9, X65-X65.9, Y15-Y15.9, Z81.1	291-291.9, 303-303.93, 305-305.03, 790.3, E860-E860.19, V11.3
3	B.7.2	Drug use disorders	F11-F19.99, P96.1, R78.1-R78.9, Z81.2-Z81.4	292-292.9, 304-304.93, 305.1-305.93, E850.0-E850.29, V15.8-V15.83, V15.85-V15.86, nan
2	B.8	Diabetes and kidney diseases	D63.1, E08-E08.9, E10-E14.9, I12-I13.9, N00-N08.8, N15.0, N17-N19, Q60-Q63.2, Q63.8-Q63.9, Q64.2-Q64.9, R73-R73.9, Z13.1, Z49-Z49.32, Z52.4, Z83.3, Z99.2	249-250.99, 285.21, 362.0-362.07, 403-404.93, 580-587.9, 753.0-753.4, 753.6-753.9, 790.2-790.29, V13.03-V13.09, V18-V18.0, V18.6, V18.69, V42.0, V42.83, V45.1-V45.12, V45.73, V45.85, V56-V56.8, V58.67, V59.4, V77.1, V81.5-V81.6
3	B.8.1	Diabetes mellitus	E08-E08.11, E08.3-E08.9, E10-E10.11, E10.3-E11.1, E11.3-E12.1, E12.3-E13.11, E13.3-E14.1, E14.3-E14.9, R73-R73.9, Z13.1, Z83.3	249-249.31, 249.5-250.39, 250.5-250.99, 362.0-362.07, 790.2-790.29, V18-V18.0, V42.83, V45.85, V58.67, V77.1
3	B.8.2	Chronic kidney disease	D63.1, E08.2-E08.29, E10.2-E10.29, E11.2-E11.29, E12.2, E13.2-E13.29, E14.2, I12-I13.9, N02-N08.8, N15.0, N17-N19, Q60-Q63.2, Q63.8-Q63.9, Q64.2-Q64.9, Z49-Z49.32, Z52.4, Z99.2	249.4-249.41, 250.4-250.49, 285.21, 403-404.93, 581-587.9, 753.0-753.4, 753.6-753.9, V13.03-V13.09, V18.6, V18.69, V42.0, V45.1-V45.12, V45.73, V56-V56.8, V59.4, V81.5-V81.6
3	B.8.3	Acute glomerulonephritis	N00-N01.9	580-580.9
2	B.9	Skin and subcutaneous diseases	A46-A46.0, A66-A67.3, A67.9, B07-B09, B35-B36.9, B85-B88.9, D86.3, E80.1-E80.29, I89.1-I89.8, L00-L05.92, L08-L08.9, L10-L14.0, L20-L23.2, L23.4-L27, L27.2-L30.9, L40-L45, L49-L54.0, L56, L56.2-L57.9, L59-L60.9, L62-L64, L64.8-L68.9, L70-L75.9, L80-L92.9, L94-L95.9, L97-L99.8,	035-035.9, 040.0, 078.0-078.19, 102-103.9, 110-111.9, 132-134.9, 136.1, 457.2-457.3, 680-709.3, 709.8-709.9, 728.86, 782-782.9, 785.4, V13.3, V19.4, V42.3, V43.83, V58.77, V59.1, V82.0

Level		Cause	ICD10	ICD9
			M72.5-M72.6, N49.2-N49.3, R20-R24.0, Z20.7, Z41.1, Z84.0, Z87.2, Z94.5	
3	B.9.1	Dermatitis	L20-L23.2, L23.4-L27, L27.2-L27.9, L30-L30.2, L30.5-L30.9	690-692.7, 692.79-692.9
3	B.9.2	Psoriasis	L40-L41.9	696-696.9
3	B.9.3	Bacterial skin diseases	A46-A46.0, A66-A67.3, A67.9, I89.1-I89.8, L00-L05.92, L08-L08.9, L30.3-L30.4, L88, L97-L98.499, M72.5-M72.6, N49.2-N49.3	035-035.9, 040.0, 102-103.9, 457.2-457.3, 680-689, 728.86, 785.4
3	B.9.4	Scabies	B86	133-133.6
3	B.9.5	Fungal skin diseases	B35-B36.9	110-111.9
3	B.9.6	Viral skin diseases	B07-B09	078.0-078.19
3	B.9.7	Acne vulgaris	L70-L70.9	706.0-706.1
3	B.9.8	Alopecia areata	L63-L63.9	704.0-704.09
3	B.9.9	Pruritus	L29-L29.9	698-699
3	B.9.10	Urticaria	L50-L50.9	708-708.9
3	B.9.11	Decubitus ulcer	L89-L89.95	707-707.09, 707.2-707.7
3	B.9.12	Other skin and subcutaneous diseases	B85-B85.4, B87-B88.9, D86.3, E80.1-E80.29, L10-L14.0, L28-L28.2, L42-L45, L49-L49.9, L51-L54.0, L56, L56.2-L57.9, L59-L60.9, L62-L62.8, L64, L64.8-L68.9, L71-L75.9, L80-L87.9, L90-L92.9, L94-L95.9, L98.5-L99.8, Z20.7	132-132.9, 133.8-134.9, 136.1, 692.70-692.77, 693-695.9, 697-697.9, 700-704, 704.1-706, 706.2-706.9, 707.1-707.19, 707.8-707.9, 709-709.3, 709.8-709.9
2	B.10	Sense organ diseases	B30-B30.9, H00-H02.8, H02.82-H02.9, H03.0-H05.329, H05.34-H05.419, H05.8-H06.3, H10-H11.9, H13-H13.8, H15-H22.8, H25-H28.8, H30-H36.8, H40-H40.9, H42-H44.539, H44.8-H55.89, H57-H58.9, H60-H62.8, H71-H75.83, H80-H83.93, H90-H91, H91.1-H94.83, Q16-Q16.9, R43-R44.9, Z01.0-Z01.12, Z13.5, Z41.3, Z52.5, Z82.1-Z82.2, Z83.5-Z83.6, Z94.7, Z97.3-Z97.4	077-077.99, 360-360.44, 360.8-362, 362.1-374.85, 374.87-376.52, 376.8-379.59, 379.8-380.9, 384-389.9, 744.0, V19.0-V19.3, V42.5, V43.0-V43.1, V45.6-V45.69, V45.78, V48.4-V48.5, V50.3, V58.71, V59.5, V74.4, V80-V80.0, V80.09-V80.3
3	B.10.1	Blindness and vision loss	H25-H28.8, H31-H36.8, H40-H40.9, H42-H42.8, H46-H54.9	360.8-362, 362.1-363.9, 365-369.9, 377-378.9
3	B.10.2	Age-related and other hearing loss	H71-H75.83, H80-H80.93, H83-H83.93, H90-H91, H91.1-H91.93, H94-H94.83, Q16-Q16.9	384-385.9, 387-387.9, 388.1-388.2, 389-389.9, 744.0
3	B.10.3	Other sense organ diseases	B30-B30.9, H00-H02.8, H02.82-H02.9, H03.0-H05.329, H05.34-H05.419, H05.8-H06.3, H10-H11.9, H13-H13.8, H15-H22.8, H30-H30.93, H43-H44.539, H44.8-H45.8, H55-H55.89, H57-H58.9, H60-H62.8, H81-H82.9, H92-H93.93, R43-R44.9	077-077.99, 360-360.44, 364-364.9, 370-374.85, 374.87-376.52, 376.8-376.9, 379-379.59, 379.8-380.9, 386-386.9, 388-388.02, 388.3-388.9

Level		Cause	ICD10	ICD9
2	B.11	Musculoskeletal disorders	G54.2-G54.4, I27.1, L93-L93.2, M00-M03.6, M05-M10.19, M10.3-M25.9, M30-M32.9, M34-M36.8, M40-M43.9, M45-M49, M49.2-M51.9, M53-M54.9, M61-M63.89, M65-M68.8, M70-M72.4, M72.8-M73, M75-M77.9, M79-M79.676, M79.8-M87.09, M87.2-M89.59, M89.7-M95.9, M99-M99.9, Z13.82-Z13.83, Z82.6-Z82.69, Z87.3-Z87.39	274-274.9, 353.1-353.4, 416.1, 446-446.9, 710-710.2, 710.5-724.9, 726-727.9, 729-730.39, 730.7-739.9, V13.4-V13.59, V17.7-V17.89, V43.6-V43.8, V58.64-V58.65, V58.78, V77.5, V82.1-V82.2
3	B.11.1	Rheumatoid arthritis	M05-M05.9, M08-M09.8	714-714.9
3	B.11.2	Osteoarthritis	M16-M18.9	715-715.98
3	B.11.3	Low back pain	G54.4, M47.015-M47.019, M47.15-M47.18, M47.25-M47.28, M47.815-M47.818, M47.896-M47.899, M48.05-M48.08, M48.16-M48.19, M48.25-M48.27, M48.35-M48.38, M48.45-M48.48, M48.55-M48.58, M49.85-M49.88, M51.05-M51.07, M51.15-M51.17, M51.25-M51.27, M51.35-M51.37, M51.45-M51.47, M51.85-M51.87, M53.3, M53.85-M53.88, M54.05-M54.09, M54.15-M54.18, M54.3-M54.5, M99.03-M99.04, M99.13-M99.14, M99.23-M99.24, M99.33-M99.34, M99.43-M99.44, M99.53-M99.54, M99.63-M99.64, M99.73-M99.74, M99.83-M99.84	353.1, 353.4, 721.3, 721.42, 722.10, 722.32, 722.52, 722.73, 722.83, 722.93, 724.02-724.03, 724.2-724.3, 724.6-724.79
3	B.11.4	Neck pain	G54.2, M47.011-M47.013, M47.11-M47.13, M47.21-M47.23, M47.811-M47.813, M47.892-M47.894, M48.01-M48.03, M48.12-M48.14, M48.21-M48.23, M48.31-M48.33, M48.41-M48.43, M48.51-M48.53, M49.81-M49.83, M50-M50.93, M53.0-M53.1, M53.81-M53.83, M54.01-M54.03, M54.11-M54.13, M54.2, M54.81, M99.01, M99.11, M99.21, M99.31, M99.41, M99.51, M99.61, M99.71, M99.81	353.2, 721.0-721.1, 722.0, 722.71, 722.81, 722.91, 723-723.9
3	B.11.5	Gout	M10-M10.19, M10.3-M10.9, M1A00X0-M1A9XX1	274-274.9, 712.0-712.09
3	B.11.6	Other musculoskeletal disorders	G54.3, I27.1, L93-L93.2, M00-M03.6, M06-M07.69, M11-M15.9, M19-M19.93, M20-M25.9, M30-M32.9, M34-M36.8, M40-M43.9, M45-M47.01, M47.014, M47.02-M47.10, M47.14, M47.2-M47.20, M47.24, M47.8-M47.81, M47.814, M47.819-M47.891, M47.895, M47.9-M48.00, M48.04, M48.1-M48.11, M48.15, M48.2-M48.20, M48.24, M48.3-M48.30, M48.34, M48.4-M48.40, M48.44, M48.5-M48.50, M48.54, M48.8-M49, M49.2-M49.80, M49.84, M49.89, M51-M51.04, M51.1-M51.14, M51.2-M51.24, M51.3-M51.34, M51.4-M51.44, M51.8-M51.84, M51.9, M53, M53.2, M53.8-M53.80,	353.3, 416.1, 446-446.9, 710-710.2, 710.5-712, 712.1-713.8, 716-721, 721.2, 721.4-721.41, 721.5-722, 722.1, 722.11-722.31, 722.39-722.51, 722.6-722.70, 722.72, 722.8-722.80, 722.82, 722.9-722.90, 722.92, 724-724.01, 724.09-724.1, 724.4-724.5, 724.8-724.9, 726-727.9, 729-730.39, 730.7-739.9

Level		Cause	ICD10	ICD9
			M53.84, M53.9-M54.00, M54.04, M54.1-M54.10, M54.14, M54.6-M54.8, M54.89-M54.9, M61-M63.89, M65-M68.8, M70-M72.4, M72.8-M73, M75-M77.9, M79-M79.676, M79.8-M87.09, M87.2-M89.59, M89.7-M95.9, M99-M99.00, M99.02, M99.05-M99.10, M99.12, M99.15-M99.20, M99.22, M99.25-M99.30, M99.32, M99.35-M99.40, M99.42, M99.45-M99.50, M99.52, M99.55-M99.60, M99.62, M99.65-M99.70, M99.72, M99.75-M99.80, M99.82, M99.85-M99.9	
2	B.12	Other non-communicable diseases	B37.3-B37.49, C7A00-C7B8, D25-D26, D3A00-D3A8, D55-D61.9, D64-D69.49, D69.6-D70.0, D70.4-D77, D80-D84.9, D86.8, D86.82-D86.84, D86.86-D86.89, D89-D89.2, D89.8, D89.82-D89.9, E03-E03.1, E03.3-E06.3, E06.5-E07.9, E15-E16, E16.1-E16.9, E20-E23.0, E23.2-E24.1, E24.3, E24.8-E27.2, E27.4-E32.9, E34, E34.1-E35.8, E65-E66.09, E66.2-E68, E70-E80.09, E80.3-E85.9, E88-E88.9, E90-E998, G71.2, K00-K08.499, K08.8-K14.9, M26-M27.9, N10-N13.9, N15, N15.1-N16.8, N20-N23.0, N25-N29, N29.1-N30.31, N30.8-N37.8, N39-N46.02, N46.022-N46.12, N46.122-N49.1, N49.8-N52.1, N52.8-N53.9, N61-N64.9, N72-N72.0, N75-N77.8, N80-N81.9, N83-N84, N84.2-N86, N88-N95.9, N97-N98.9, P96.0, Q00-Q07.9, Q10-Q15.9, Q17-Q18.9, Q20-Q28.9, Q30-Q45.9, Q50-Q56.4, Q63.3, Q64-Q64.19, Q65-Q87.89, Q89-Q89.8, Q90-Q93.9, Q95-Q99.9, R30-R37, R39-R39.9, R86-R87.9, Z01.2-Z01.21, Z01.4-Z01.7, Z13.7-Z13.79, Z13.84, Z14-Z15.89, Z31-Z31.9, Z43.5-Z43.7, Z82.7-Z82.79, Z83.4-Z83.49, Z84.1-Z84.2, Z86.1-Z86.19, Z87.4-Z87.448, Z87.7-Z87.798, Z96.5	112.1-112.2, 218-218.9, 237.7, 237.73-237.79, 240-244, 244.8-246.9, 251-251.2, 251.4-253.6, 253.8-259.9, 270-273.9, 275-278.02, 278.1-279.49, 279.6-279.9, 282-285.0, 285.8-289.9, 520-525.54, 525.8-526.61, 526.69-529.9, 588-595.81, 595.89-596.8, 596.89-598.1, 598.8-609, 611-611.9, 616-618.9, 620-622.0, 622.3-622.6, 622.8-629.9, 740-744, 744.00-753, 753.5, 754-759.9, 775.3, 788-788.99, 799.81, V07.31, V07.4-V07.59, V12.2-V12.49, V13.0-V13.02, V13.2, V13.29, V13.6-V13.69, V18.1-V18.19, V18.61, V18.7, V18.9, V19.5, V19.7-V19.8, V26-V26.9, V43.82, V45.71, V45.74, V45.83-V45.84, V45.86, V47.4-V47.5, V49.81-V49.82, V58.5, V58.76, V59.7-V59.74, V72.2-V72.31, V77.0, V77.3-V77.4, V77.6-V77.8, V77.91, V78, V78.2-V78.9, V82.3, V85-V85.45, V85.51, V85.53-V85.54
3	B.12.1	Congenital birth defects	G71.2, P96.0, Q00-Q07.9, Q10-Q15.9, Q17-Q18.9, Q20-Q28.9, Q30-Q45.9, Q50-Q56.4, Q63.3, Q64-Q64.19, Q65-Q87.89, Q89-Q89.8, Q90-Q93.9, Q95-Q99.9, Z13.7-Z13.79, Z14-Z15.89, Z82.7-Z82.79, Z87.7-Z87.798	237.7, 237.73-237.79, 740-744, 744.00-753, 753.5, 754-759.9, V13.6-V13.69, V18.61, V18.9, V19.5, V19.7-V19.8, V82.3
3	B.12.2	Urinary diseases and male infertility	N10-N13.9, N15, N15.1-N16.8, N20-N23.0, N25-N29, N29.1-N30.31, N30.8-N37.8, N39-N46.02, N46.022-N46.12, N46.122-N49.1, N49.8-N52.1, N52.8-N53.9, R86-R86.9, Z43.5-Z43.7, Z84.1-Z84.2	588-595.81, 595.89-596.8, 596.89-598.1, 598.8-609, 788.3-788.39, 788.91, V13.0-V13.02, V26.5, V26.52, V45.74, V47.4, V58.76

Level		Cause	ICD10	ICD9
3	B.12.3	Gynecological diseases	B37.3-B37.49, D25-D26, E28.2, N61-N64.9, N72-N72.0, N75-N77.8, N80-N81.9, N83-N84, N84.2-N86, N88-N95.9, N97-N98.9, R30-R37, R39-R39.9, R87-R87.9, Z01.4-Z01.7, Z31-Z31.9, Z86.1-Z86.19, Z87.4-Z87.448	112.1-112.2, 218-218.9, 256.4, 611-611.9, 616-618.9, 620-622.0, 622.3-622.6, 622.8-629.9, 788-788.29, 788.4-788.9, 788.99, 799.81, V07.4-V07.59, V13.2, V13.29, V18.7, V26-V26.49, V26.51, V26.8-V26.9, V43.82, V45.71, V45.83, V47.5, V49.81, V59.7-V59.74, V72.3-V72.31
3	B.12.4	Hemoglobinopathies and hemolytic anemias	D55-D61.9, D64-D64.8	282-285.0, 285.8-285.9, V78, V78.2-V78.9
3	B.12.5	Endocrine, metabolic, blood, and immune disorders	C7A00-C7B8, D3A00-D3A8, D66-D69.49, D69.6-D70.0, D70.4-D77, D80-D84.9, D86.8, D86.82-D86.84, D86.86-D86.89, D89-D89.2, D89.8, D89.82-D89.9, E03-E03.1, E03.3-E06.3, E06.5-E07.9, E15-E16, E16.1-E16.9, E20-E23.0, E23.2-E24.1, E24.3, E24.8-E27.2, E27.4-E28.1, E28.3-E32.9, E34, E34.1-E35.8, E65-E66.09, E66.2-E68, E70-E80.09, E80.3-E85.9, E88-E88.9, E90-E998, Z83.4-Z83.49	240-244, 244.8-246.9, 251-251.2, 251.4-253.6, 253.8-256.39, 256.8-259.9, 270-273.9, 275-278.02, 278.1-279.49, 279.6-279.9, 286-289.9, 775.3, V12.2-V12.49, V18.1-V18.19, V45.86, V77.0, V77.3-V77.4, V77.6-V77.8, V77.91, V85-V85.45, V85.51, V85.53-V85.54
3	B.12.6	Oral disorders	K00-K08.499, K08.8-K14.9, M26-M27.9, Z01.2-Z01.21, Z13.84, Z96.5	520-525.54, 525.8-526.61, 526.69-529.9, V07.31, V45.84, V49.82, V58.5, V72.2
2	C.1	Transport injuries	V00-V86.99, V87.2-V87.3, V88.2-V88.3, V90-V98.8	E800-E800.3, E801-E801.3, E802-E802.3, E803-E803.3, E804-E804.3, E805-E805.3, E806-E806.3, E807-E807.3, E810.0-E810.7, E811.0-E811.7, E812.0-E812.7, E813.0-E813.7, E814.0-E814.7, E815.0-E815.7, E816.0-E816.7, E817.0-E817.7, E818.0-E818.7, E819.0-E819.7, E820.0-E820.7, E821.0-E821.7, E822.0-E822.7, E823.0-E823.7, E824.0-E824.7, E825.0-E825.7, E826.0-E826.4, E827.0-E827.4, E828.0-E828.4, E829.0-E829.4, E830-E838.9, E840-E849.9, E929.1, V03, V07.8-V07.9, V13, V13.8, V13.9, V15.2, V15.3, V15.9, V19, V42, V42.8, V42.9-V43, V47-V47.1
3	C.1.1	Road injuries	V01-V04.99, V06-V80.929, V82-V82.9, V87.2-V87.3	E800.3, E801.3, E802.3, E803.3, E804.3, E805.3, E806.3, E807.3, E810.0-E810.6, E811.0-E811.7, E812.0-E812.7, E813.0-E813.7, E814.0-E814.7, E815.0-E815.7, E816.0-E816.7, E817.0-E817.7, E818.0-E818.7, E819.0-E819.7, E820.0-E820.6, E821.0-E821.6, E822.0-E822.7, E823.0-

Level		Cause	ICD10	ICD9
3	C.1.2	Other transport injuries	V00-V00.898, V05-V05.99, V81-V81.9, V83-V86.99, V88.2-V88.3, V90-V98.8	E823.7, E824.0-E824.7, E825.0-E825.7, E826.0-E826.1, E826.3-E826.4, E827.0, E827.3-E827.4, E828.0, E828.4, E829.0-E829.4, V03, V07.8-V07.9, V13, V13.8, V13.9, V15.2, V15.3, V15.9, V19, V42, V42.8, V42.9-V43, V47-V47.1 E800-E800.2, E801-E801.2, E802-E802.2, E803-E803.2, E804-E804.2, E805-E805.2, E806-E806.2, E807-E807.2, E810.7, E820.7, E821.7, E826.2, E827.2, E828.2, E830-E838.9, E840-E849.9, E929.1
2	C.2	Unintentional injuries	D69.5-D69.59, D70.1-D70.2, D78-D78.89, D89.81-D89.813, E03.2, E06.4, E09-E09.9, E16.0, E23.1, E24.2, E27.3, E36-E36.8, E66.1, E86.02-E87.99, E89-E89.9, G21.0-G21.19, G24.0-G24.09, G25.1, G25.4, G25.6-G25.79, G62.0, G72.0, G93.7, G96.0, G96.11, G97-G97.9, H02.81-H02.819, H05.33-H05.339, H05.42-H05.53, H44.6-H44.799, H59-H59.89, H91.0-H91.09, H95-H95.9, I95.2-I95.81, I97-I97.9, J70-J70.5, J95-J95.9, K08.5-K08.59, K43-K43.9, K52.0, K62.7, K68.11, K91-K91.9, K94-K95.89, L23.3, L27.0-L27.1, L55-L55.9, L56.0-L56.1, L58-L58.9, L64.0, L76-L76.82, M10.2-M10.29, M60.2-M60.28, M87.1-M87.19, M96-M96.9, N14-N14.4, N30.4-N30.41, N46.021, N46.121, N52.2-N52.39, N65-N65.1, N99-N99.9, P93-P93.8, P96.2, P96.5, R50.2-R50.83, W00-W46.2, W49-W62.9, W64-W70.9, W73-W81.9, W83-W94.9, W97.9, W99-X06.9, X08-X44.9, X46-X58.9, Y10-Y14.9, Y16-Y19.9, Y40-Y84.9, Y88-Y88.3, Z21.0, Z42-Z43.0, Z43.8-Z43.9, Z48-Z48.9, Z51-Z51.9, Z88-Z88.9, Z92-Z94.0, Z94.6, Z94.8-Z94.9, Z96-Z96.49, Z96.6-Z97.2, Z97.8-Z99.12, Z99.3-Z99.9	244.0-244.1, 244.3, 251.3, 253.7, 279.5-279.53, 331.81, 333.92, 349-349.9, 357.6, 359.24, 360.5-360.69, 374.86, 376.6, 379.6-379.63, 440.3-440.32, 457.0, 458.2-458.29, 518.6-518.7, 519.0-519.1, 525.6-525.79, 526.62-526.63, 530.86-530.87, 536.4-536.49, 539-539.9, 551.2-551.29, 552.2-552.29, 553.2-553.29, 564.2-564.4, 569.6-569.8, 579.3, 595.82, 596.81-596.83, 598.2, 612-612.1, 709.4, 770.1-770.18, 779.4-779.5, 780.62-780.66, 995.89, E850.3-E858.99, E862-E869.99, E870-E876.9, E878-E879.9, E880-E886.99, E888-E928.89, E929.2-E929.5, E930-E949.9, V44-V45, V45.2-V45.4, V45.7, V45.77, V45.79-V45.8, V45.87-V45.89
3	C.2.1	Falls	W00-W19.9	E880-E886.99, E888-E888.9, E929.3
3	C.2.2	Drowning	W65-W70.9, W73-W74.9	E910-E910.99
3	C.2.3	Fire, heat, and hot substances	X00-X06.9, X08-X19.9	E890-E899.09, E924-E924.99, E929.4
3	C.2.4	Poisonings	E86.02-E86.99, J70.5, X40-X44.9, X46-X49.9, Y10-Y14.9, Y16-Y19.9	E850.3-E858.99, E862-E869.99, E929.2
3	C.2.5	Exposure to mechanical forces	W20-W38.9, W40-W43.9, W45.0-W45.2, W46-W46.2, W49-W52	E916-E922.99, E928.1-E928.7

Level		Cause	ICD10	ICD9
3	C.2.6	Adverse effects of medical treatment	D69.5-D69.59, D70.1-D70.2, D78-D78.89, D89.81-D89.813, E03.2, E06.4, E09-E09.9, E16.0, E23.1, E24.2, E27.3, E36-E36.8, E66.1, E87.0-E87.99, E89-E89.9, G21.0-G21.19, G24.0-G24.09, G25.1, G25.4, G25.6-G25.79, G62.0, G72.0, G93.7, G96.0, G96.11, G97-G97.9, H05.33-H05.339, H05.42-H05.53, H59-H59.89, H91.0-H91.09, H95-H95.9, I95.2-I95.81, I97-I97.9, J70-J70.4, J95-J95.9, K08.5-K08.59, K43-K43.9, K52.0, K62.7, K68.11, K91-K91.9, K94-K95.89, L23.3, L27.0-L27.1, L56.0-L56.1, L64.0, L76-L76.82, M10.2-M10.29, M87.1-M87.19, M96-M96.9, N14-N14.4, N30.4-N30.41, N46.021, N46.121, N52.2-N52.39, N65-N65.1, N99-N99.9, P93-P93.8, P96.2, P96.5, R50.2-R50.83, Y40-Y84.9, Y88-Y88.3, Z21.0, Z42-Z43.0, Z43.8-Z43.9, Z48-Z48.9, Z51-Z51.9, Z88-Z88.9, Z92-Z94.0, Z94.6, Z94.8-Z94.9, Z96-Z96.49, Z96.6-Z97.2, Z97.8-Z99.12, Z99.3-Z99.9	244.0-244.1, 244.3, 251.3, 253.7, 279.5-279.53, 331.81, 333.92, 349-349.9, 357.6, 359.24, 379.6-379.63, 440.3-440.32, 457.0, 458.2-458.29, 518.6-518.7, 519.0-519.1, 525.6-525.79, 526.62-526.63, 530.86-530.87, 536.4-536.49, 539-539.9, 551.2-551.29, 552.2-552.29, 553.2-553.29, 564.2-564.4, 569.6-569.8, 579.3, 595.82, 596.81-596.83, 598.2, 612-612.1, 779.4-779.5, 780.62-780.66, 995.89, E870-E876.9, E878-E879.9, E930-E949.9, V44-V45, V45.2-V45.4, V45.7, V45.77, V45.79-V45.8, V45.87-V45.89
3	C.2.7	Animal contact	W52.0-W62.9, W64-W64.9, X20-X29.9	E905-E906.99
3	C.2.8	Foreign body	H02.81-H02.819, H44.6-H44.799, M60.2-M60.28, W44-W45, W45.3-W45.9, W75-W76.9, W78-W80.9, W83-W84.9	360.5-360.69, 374.86, 376.6, 709.4, 770.1-770.18, E911-E912.09, E913.8-E915.09
3	C.2.9	Environmental heat and cold exposure	L55-L55.9, L58-L58.9, W88-W94.9, W97.9, W99-W99.9, X30-X32.9, X39-X39.9	E900-E902.99, E926-E926.99, E929.5
3	C.2.10	Exposure to forces of nature	X33-X38.9	E907-E909.9
3	C.2.11	Other unintentional injuries	W39-W39.9, W77-W77.9, W81-W81.9, W85-W87.9, X50-X58.9	E903-E904.99, E913.2-E913.39, E923-E923.99, E927-E928.09, E928.8-E928.89
2	C.3	Self-harm and interpersonal violence	T74.2-U03, X60-X64.9, X66-Y08.9, Y35-Y38.9, Y87.0-Y87.2, Y89.0-Y89.1	E950-E979.9, E990-E999.1
3	C.3.1	Self-harm	X60-X64.9, X66-X84.9, Y87.0	E950-E959
3	C.3.2	Interpersonal violence	T74.2-T76.22, X85-Y08.9, Y87.1-Y87.2	E960-E969
3	C.3.3	Conflict and terrorism	U00-U03, Y36-Y38.9, Y89.1	E979-E979.9, E990-E999.1
3	C.3.4	Executions and police conflict	Y35-Y35.93, Y89.0	E970-E978

Note: A, communicable, maternal, neonatal, and nutritional diseases; B, non-communicable diseases; C, injuries. A small number of causes don't use ICD codes.

Table S2 | Cause-specific effects of mortality and disability (years) on changes in life expectancy (LE) and health-adjusted life expectancy (HALE) from 1990 to 2019.

Causes	Level	ΔHALE			ΔLE	ΔLED
		Mortality Effect	Disability Effect	Total Effect	Total Effect	
**Communicable, maternal, neonatal, and nutritional diseases**	A	1.0925	0.2290	1.3216	3.4591	2.1376
HIV/AIDS and sexually transmitted infections	A.1	-0.0462	-0.0029	-0.0491	-0.0319	0.0173
HIV/AIDS	A.1.1	-0.0484	-0.0033	-0.0517	-0.0386	0.0131
Sexually transmitted infections excluding HIV	A.1.2	0.0022	0.0004	0.0026	0.0068	0.0042
Respiratory infections and tuberculosis	A.2	0.7790	0.0430	0.8219	1.8622	1.0403
Tuberculosis	A.2.1	0.3600	0.0328	0.3927	0.3786	-0.0141
Lower respiratory infections	A.2.2	0.3850	0.0033	0.3884	1.4458	1.0575
Upper respiratory infections	A.2.3	0.0336	-0.0004	0.0331	0.0374	0.0042
Otitis media	A.2.4	0.0004	0.0073	0.0077	0.0004	-0.0073
Enteric infections	A.3	0.0719	0.0004	0.0723	0.2872	0.2148
Diarrheal diseases	A.3.1	0.0642	0.0004	0.0645	0.2813	0.2168
Typhoid and paratyphoid	A.3.2	0.0059	0.0001	0.0060	0.0035	-0.0025
Invasive Non-typhoidal Salmonella (iNTS)	A.3.3	0.0013	0.0000	0.0013	0.0012	-0.0001
Other intestinal infectious diseases	A.3.4	0.0004	0.0000	0.0004	0.0011	0.0006
Neglected tropical diseases and malaria	A.4	0.0136	0.0994	0.1130	0.0143	-0.0986
Malaria	A.4.1	0.0046	0.0007	0.0053	0.0039	-0.0014
Chagas disease	A.4.2	0.0000	0.0000	0.0000	0.0000	0.0000
Leishmaniasis	A.4.3	0.0000	0.0000	0.0000	0.0000	0.0000
African trypanosomiasis	A.4.4	0.0000	0.0000	0.0000	0.0000	0.0000
Schistosomiasis	A.4.5	0.0047	0.0043	0.0091	0.0044	-0.0047
Cysticercosis	A.4.6	0.0002	0.0059	0.0061	0.0002	-0.0060
Cystic echinococcosis	A.4.7	0.0001	0.0000	0.0001	0.0001	0.0000
Lymphatic filariasis	A.4.8	0.0000	0.0000	0.0000	0.0000	0.0000
Onchocerciasis	A.4.9	0.0000	0.0000	0.0000	0.0000	0.0000
Trachoma	A.4.10	0.0000	0.0032	0.0032	0.0000	-0.0032
Dengue	A.4.11	0.0001	-0.0022	-0.0021	0.0002	0.0023
Yellow fever	A.4.12	0.0000	0.0000	0.0000	0.0000	0.0000
Rabies	A.4.13	0.0021	0.0000	0.0021	0.0024	0.0003
Intestinal nematode infections	A.4.14	0.0007	0.0409	0.0416	0.0013	-0.0403
Food-borne trematodiases	A.4.15	0.0000	0.0380	0.0380	0.0000	-0.0380
Leprosy	A.4.16	0.0000	0.0000	0.0000	0.0000	0.0000
Ebola	A.4.17	0.0000	0.0000	0.0000	0.0000	0.0000
Zika virus	A.4.18	0.0000	0.0000	0.0000	0.0000	0.0000
Guinea worm disease	A.4.19	0.0000	0.0000	0.0000	0.0000	0.0000
Other neglected tropical diseases	A.4.20	0.0011	0.0084	0.0096	0.0019	-0.0077
Other infectious diseases	A.5	0.1677	0.0183	0.1860	0.4507	0.2647
Meningitis	A.5.1	0.0393	0.0050	0.0443	0.0993	0.0550
Encephalitis	A.5.2	0.0068	0.0031	0.0099	0.0196	0.0097
Diphtheria	A.5.3	0.0003	0.0000	0.0003	0.0006	0.0003
Whooping cough	A.5.4	0.0167	0.0010	0.0177	0.0949	0.0773

Causes	Level	ΔHALE			ΔLE	ΔLED
		Mortality Effect	Disability Effect	Total Effect	Total Effect	
Tetanus	A.5.5	0.0132	0.0001	0.0133	0.0581	0.0448
Measles	A.5.6	0.0276	0.0007	0.0282	0.0912	0.0630
Varicella and herpes zoster	A.5.7	0.0064	0.0000	0.0064	0.0068	0.0004
Acute hepatitis	A.5.8	0.0490	0.0013	0.0503	0.0621	0.0118
Other unspecified infectious diseases	A.5.9	0.0084	0.0072	0.0156	0.0181	0.0025
Maternal and neonatal disorders	A.6	0.0717	-0.0916	-0.0199	0.7633	0.7833
Maternal disorders	A.6.1	0.0634	0.0039	0.0673	0.0355	-0.0318
Neonatal disorders	A.6.2	0.0083	-0.0955	-0.0872	0.7279	0.8151
Nutritional deficiencies	A.7	0.0348	0.1625	0.1973	0.1132	-0.0842
Protein-energy malnutrition	A.7.1	0.0358	-0.0135	0.0223	0.1105	0.0882
Iodine deficiency	A.7.2	0.0000	0.0028	0.0028	0.0000	-0.0028
Vitamin A deficiency	A.7.3	0.0000	0.0035	0.0035	0.0000	-0.0035
Dietary iron deficiency	A.7.4	0.0000	0.1719	0.1719	0.0000	-0.1719
Other nutritional deficiencies	A.7.5	-0.0010	-0.0022	-0.0032	0.0026	0.0058
**Non-communicable diseases**	B	6.0503	0.2284	6.2788	5.8257	-0.4530
Neoplasms	B.1	0.7057	-0.0425	0.6632	0.8299	0.1667
Lip and oral cavity cancer	B.1.1	-0.0036	-0.0005	-0.0041	-0.0036	0.0005
Nasopharynx cancer	B.1.2	0.0340	-0.0018	0.0323	0.0318	-0.0005
Other pharynx cancer	B.1.3	0.0018	-0.0001	0.0017	0.0017	0.0000
Esophageal cancer	B.1.4	0.1554	0.0016	0.1570	0.1679	0.0108
Stomach cancer	B.1.5	0.2938	0.0001	0.2939	0.3065	0.0127
Colon and rectum cancer	B.1.6	-0.0652	-0.0120	-0.0772	-0.0522	0.0250
Liver cancer	B.1.7	0.3395	0.0034	0.3428	0.3445	0.0016
Gallbladder and biliary tract cancer	B.1.8	-0.0031	-0.0002	-0.0033	-0.0023	0.0009
Pancreatic cancer	B.1.9	-0.0472	-0.0007	-0.0479	-0.0419	0.0059
Larynx cancer	B.1.10	0.0074	-0.0005	0.0069	0.0079	0.0010
Tracheal, bronchus, and lung cancer	B.1.11	-0.1166	-0.0045	-0.1211	-0.0717	0.0493
Malignant skin melanoma	B.1.12	0.0012	-0.0003	0.0009	0.0012	0.0003
Non-melanoma skin cancer	B.1.13	-0.0030	-0.0001	-0.0032	-0.0018	0.0014
Breast cancer	B.1.14	0.0024	-0.0071	-0.0047	0.0036	0.0083
Cervical cancer	B.1.15	0.0076	-0.0008	0.0068	0.0065	-0.0003
Uterine cancer	B.1.16	0.0123	-0.0005	0.0118	0.0123	0.0004
Ovarian cancer	B.1.17	-0.0087	-0.0006	-0.0093	-0.0095	-0.0002
Prostate cancer	B.1.18	0.0007	-0.0044	-0.0037	0.0030	0.0067
Testicular cancer	B.1.19	0.0002	-0.0004	-0.0002	0.0002	0.0004
Kidney cancer	B.1.20	-0.0116	-0.0010	-0.0126	-0.0092	0.0034
Bladder cancer	B.1.21	0.0064	-0.0017	0.0047	0.0062	0.0015
Brain and central nervous system cancer	B.1.22	0.0107	-0.0008	0.0099	0.0165	0.0066
Thyroid cancer	B.1.23	0.0010	-0.0005	0.0005	0.0010	0.0006
Mesothelioma	B.1.24	-0.0002	0.0000	-0.0002	-0.0002	0.0000
Hodgkin lymphoma	B.1.25	0.0096	-0.0001	0.0095	0.0084	-0.0011
Non-Hodgkin lymphoma	B.1.26	-0.0090	-0.0029	-0.0119	-0.0073	0.0046
Multiple myeloma	B.1.27	-0.0012	-0.0003	-0.0014	-0.0005	0.0010

Causes	Level	ΔHALE			ΔLE	ΔLED
		Mortality Effect	Disability Effect	Total Effect	Total Effect	
Leukemia	B.1.28	0.0692	-0.0005	0.0688	0.0826	0.0139
Other malignant neoplasms	B.1.29	0.0227	-0.0039	0.0188	0.0289	0.0101
Other neoplasms	B.1.30	-0.0010	-0.0013	-0.0024	-0.0006	0.0018
Cardiovascular diseases	B.2	1.8440	-0.0776	1.7664	1.8217	0.0553
Rheumatic heart disease	B.2.1	0.2780	0.0013	0.2792	0.2372	-0.0420
Ischemic heart disease	B.2.2	-0.2965	-0.0040	-0.3004	-0.0680	0.2324
Stroke	B.2.3	1.4604	-0.0730	1.3875	1.3135	-0.0740
Hypertensive heart disease	B.2.4	0.3703	0.0040	0.3743	0.2936	-0.0806
Non-rheumatic valvular heart disease	B.2.5	0.0050	-0.0010	0.0039	0.0037	-0.0003
Cardiomyopathy and myocarditis	B.2.6	0.0018	0.0000	0.0018	0.0109	0.0091
Atrial fibrillation and flutter	B.2.8	0.0035	-0.0039	-0.0004	0.0035	0.0040
Aortic aneurysm	B.2.9	0.0010	0.0000	0.0010	0.0012	0.0002
Peripheral artery disease	B.2.10	-0.0004	0.0016	0.0012	-0.0002	-0.0014
Endocarditis	B.2.11	0.0042	0.0000	0.0041	0.0066	0.0025
Other cardiovascular and circulatory diseases	B.2.12	0.0168	-0.0025	0.0143	0.0197	0.0054
Chronic respiratory diseases	B.3	2.7212	0.1112	2.8324	2.0179	-0.8145
Chronic obstructive pulmonary disease	B.3.1	2.6142	0.0981	2.7123	1.9314	-0.7809
Pneumoconiosis	B.3.2	0.0165	0.0022	0.0187	0.0154	-0.0032
Asthma	B.3.3	0.0903	0.0137	0.1040	0.0694	-0.0346
Interstitial lung disease and pulmonary sarcoidosis	B.3.4	0.0003	-0.0009	-0.0006	0.0007	0.0013
Other chronic respiratory diseases	B.3.5	0.0000	-0.0019	-0.0019	0.0010	0.0029
Digestive diseases	B.4	0.4969	0.0477	0.5446	0.4885	-0.0561
Cirrhosis and other chronic liver diseases	B.4.1	0.2425	0.0030	0.2455	0.2345	-0.0110
Upper digestive system diseases	B.4.2	0.1254	0.0201	0.1455	0.1116	-0.0339
Appendicitis	B.4.3	0.0098	-0.0002	0.0096	0.0078	-0.0018
Paralytic ileus and intestinal obstruction	B.4.4	0.0205	-0.0002	0.0203	0.0423	0.0220
Inguinal, femoral, and abdominal hernia	B.4.5	0.0028	0.0017	0.0045	0.0062	0.0018
Inflammatory bowel disease	B.4.6	0.0103	-0.0032	0.0071	0.0091	0.0021
Vascular intestinal disorders	B.4.7	0.0017	0.0000	0.0017	0.0015	-0.0002
Gallbladder and biliary diseases	B.4.8	0.0351	0.0252	0.0603	0.0292	-0.0310
Pancreatitis	B.4.9	0.0117	0.0003	0.0119	0.0100	-0.0020
Other digestive diseases	B.4.10	0.0371	0.0012	0.0383	0.0363	-0.0020
Neurological disorders	B.5	0.0503	-0.0786	-0.0283	0.0514	0.0797
Alzheimer's disease and other dementias	B.5.1	0.0043	-0.0403	-0.0360	0.0043	0.0402
Parkinson's disease	B.5.2	0.0162	-0.0073	0.0089	0.0143	0.0054
Idiopathic epilepsy	B.5.3	0.0288	-0.0030	0.0258	0.0275	0.0018
Multiple sclerosis	B.5.4	0.0009	-0.0001	0.0008	0.0009	0.0000
Motor neuron disease	B.5.5	0.0010	-0.0001	0.0009	0.0031	0.0022
Headache disorders	B.5.6	0.0000	-0.0236	-0.0236	0.0000	0.0236
Other neurological disorders	B.5.7	-0.0009	-0.0042	-0.0052	0.0014	0.0065
Mental disorders	B.6	-0.0001	0.0354	0.0353	-0.0001	-0.0353
Schizophrenia	B.6.1	0.0000	-0.0057	-0.0057	0.0000	0.0057
Depressive disorders	B.6.2	0.0000	0.0192	0.0192	0.0000	-0.0192

Causes	Level	ΔHALE			ΔLE	ΔLED
		Mortality Effect	Disability Effect	Total Effect	Total Effect	
Bipolar disorder	B.6.3	0.0000	-0.0001	-0.0001	0.0000	0.0001
Anxiety disorders	B.6.4	0.0000	0.0249	0.0249	0.0000	-0.0249
Eating disorders	B.6.5	-0.0001	-0.0065	-0.0065	-0.0001	0.0065
Autism spectrum disorders	B.6.6	0.0000	-0.0015	-0.0015	0.0000	0.0015
Attention-deficit/hyperactivity disorder	B.6.7	0.0000	-0.0010	-0.0010	0.0000	0.0010
Conduct disorder	B.6.8	0.0000	-0.0015	-0.0015	0.0000	0.0015
Idiopathic developmental intellectual disability	B.6.9	0.0000	0.0077	0.0077	0.0000	-0.0077
Other mental disorders	B.6.10	0.0000	-0.0002	-0.0002	0.0000	0.0002
Substance use disorders	B.7	0.0599	0.0063	0.0662	0.0419	-0.0244
Alcohol use disorders	B.7.1	-0.0032	-0.0002	-0.0034	-0.0029	0.0005
Drug use disorders	B.7.2	0.0631	0.0065	0.0696	0.0447	-0.0249
Diabetes and kidney diseases	B.8	0.0778	-0.0578	0.0200	0.0890	0.0690
Diabetes mellitus	B.8.1	-0.0010	-0.0517	-0.0527	0.0135	0.0661
Chronic kidney disease	B.8.2	0.0578	-0.0062	0.0516	0.0570	0.0054
Acute glomerulonephritis	B.8.3	0.0210	0.0000	0.0210	0.0185	-0.0025
Skin and subcutaneous diseases	B.9	0.0134	-0.0084	0.0049	0.0113	0.0064
Dermatitis	B.9.1	0.0000	-0.0012	-0.0012	0.0000	0.0012
Psoriasis	B.9.2	0.0000	0.0106	0.0106	0.0000	-0.0106
Bacterial skin diseases	B.9.3	0.0145	0.0001	0.0146	0.0119	-0.0027
Scabies	B.9.4	0.0000	0.0016	0.0016	0.0000	-0.0016
Fungal skin diseases	B.9.5	0.0000	0.0006	0.0006	0.0000	-0.0006
Viral skin diseases	B.9.6	0.0000	-0.0002	-0.0002	0.0000	0.0002
Acne vulgaris	B.9.7	0.0000	-0.0141	-0.0141	0.0000	0.0141
Alopecia areata	B.9.8	0.0000	0.0000	0.0000	0.0000	0.0000
Pruritus	B.9.9	0.0000	-0.0010	-0.0010	0.0000	0.0010
Urticaria	B.9.10	0.0000	0.0000	0.0000	0.0000	0.0000
Decubitus ulcer	B.9.11	-0.0015	-0.0003	-0.0017	-0.0008	0.0010
Other skin and subcutaneous diseases	B.9.12	0.0003	-0.0045	-0.0043	0.0002	0.0044
Sense organ diseases	B.10	0.0000	0.0491	0.0491	0.0000	-0.0491
Blindness and vision loss	B.10.1	0.0000	0.0311	0.0311	0.0000	-0.0311
Age-related and other hearing loss	B.10.2	0.0000	0.0199	0.0199	0.0000	-0.0199
Other sense organ diseases	B.10.3	0.0000	-0.0018	-0.0018	0.0000	0.0018
Musculoskeletal disorders	B.11	-0.0035	0.1084	0.1049	-0.0014	-0.1062
Rheumatoid arthritis	B.11.1	-0.0002	-0.0015	-0.0017	0.0005	0.0022
Osteoarthritis	B.11.2	0.0000	-0.0215	-0.0215	0.0000	0.0215
Low back pain	B.11.3	0.0000	0.2376	0.2376	0.0000	-0.2376
Neck pain	B.11.4	0.0000	-0.0093	-0.0093	0.0000	0.0093
Gout	B.11.5	0.0000	-0.0066	-0.0066	0.0000	0.0066
Other musculoskeletal disorders	B.11.6	-0.0033	-0.0905	-0.0938	-0.0019	0.0919
Other non-communicable diseases	B.12	0.0846	0.1352	0.2199	0.4755	0.2556
Congenital birth defects	B.12.1	0.0499	0.0078	0.0576	0.4223	0.3647
Urinary diseases and male infertility	B.12.2	0.0164	0.0053	0.0217	0.0140	-0.0077
Gynecological diseases	B.12.3	-0.0004	0.0477	0.0473	-0.0003	-0.0476

Causes	Level	ΔHALE			ΔLE	ΔLED
		Mortality Effect	Disability Effect	Total Effect	Total Effect	
Hemoglobinopathies and hemolytic anemias	B.12.4	0.0151	0.0573	0.0724	0.0210	-0.0514
Endocrine, metabolic, blood, and immune disorders	B.12.5	0.0036	0.0087	0.0123	0.0100	-0.0023
Oral disorders	B.12.6	0.0000	0.0085	0.0085	0.0000	-0.0085
**Injuries**	C	1.1618	-0.0525	1.1093	1.2036	0.0943
Transport injuries	C.1	0.2112	-0.0565	0.1547	0.2092	0.0545
Road injuries	C.1.1	0.1789	-0.0603	0.1186	0.1811	0.0625
Other transport injuries	C.1.2	0.0323	0.0038	0.0361	0.0281	-0.0080
Unintentional injuries	C.2	0.3668	-0.0063	0.3605	0.5719	0.2114
Falls	C.2.1	-0.0085	-0.0131	-0.0215	0.0208	0.0424
Drowning	C.2.2	0.2494	0.0011	0.2505	0.3204	0.0699
Fire, heat, and hot substances	C.2.3	0.0248	-0.0004	0.0244	0.0322	0.0077
Poisonings	C.2.4	0.0069	0.0000	0.0068	0.0195	0.0127
Exposure to mechanical forces	C.2.5	0.0270	-0.0097	0.0173	0.0276	0.0103
Adverse effects of medical treatment	C.2.6	0.0186	0.0000	0.0186	0.0276	0.0090
Animal contact	C.2.7	0.0171	0.0029	0.0200	0.0184	-0.0016
Foreign body	C.2.8	0.0038	0.0098	0.0136	0.0740	0.0604
Environmental heat and cold exposure	C.2.9	0.0207	0.0021	0.0227	0.0225	-0.0003
Exposure to forces of nature	C.2.10	0.0036	-0.0015	0.0021	0.0027	0.0006
Other unintentional injuries	C.2.11	0.0036	0.0025	0.0060	0.0063	0.0003
Self-harm and interpersonal violence	C.3	0.5838	0.0104	0.5941	0.4225	-0.1717
Self-harm	C.3.1	0.4787	0.0057	0.4844	0.3374	-0.1470
Interpersonal violence	C.3.2	0.0933	0.0047	0.0980	0.0778	-0.0202
Conflict and terrorism	C.3.3	0.0001	0.0000	0.0001	0.0001	0.0000
Executions and police conflict	C.3.4	0.0117	0.0000	0.0117	0.0073	-0.0044

Table S3 | Cause-specific effects of mortality and disability (years) on changes in life expectancy (LE) and health-adjusted life expectancy (HALE) from 1990 to 1999.

Causes	Level	ΔHALE			ΔLE	ΔLED
		Mortality Effect	Disability Effect	Total Effect	Total Effect	
**Communicable, maternal, neonatal, and nutritional diseases**	A	0.7619	0.1295	0.8914	1.2887	0.3973
HIV/AIDS and sexually transmitted infections	A.1	-0.0620	-0.0005	-0.0625	-0.0077	0.0548
HIV/AIDS	A.1.1	-0.0652	-0.0006	-0.0659	-0.0102	0.0556
Sexually transmitted infections excluding HIV	A.1.2	0.0032	0.0002	0.0034	0.0025	-0.0009
Respiratory infections and tuberculosis	A.2	0.4303	0.0146	0.4448	0.7645	0.3197
Tuberculosis	A.2.1	0.2843	0.0110	0.2953	0.1754	-0.1198
Lower respiratory infections	A.2.2	0.1298	0.0012	0.1310	0.5754	0.4444
Upper respiratory infections	A.2.3	0.0154	0.0002	0.0156	0.0133	-0.0023
Otitis media	A.2.4	0.0008	0.0021	0.0029	0.0003	-0.0026
Enteric infections	A.3	0.0758	0.0053	0.0810	0.1408	0.0598
Diarrheal diseases	A.3.1	0.0655	0.0052	0.0708	0.1382	0.0674
Typhoid and paratyphoid	A.3.2	0.0085	0.0000	0.0086	0.0017	-0.0068
Invasive Non-typhoidal Salmonella (iNTS)	A.3.3	0.0015	0.0000	0.0015	0.0005	-0.0010
Other intestinal infectious diseases	A.3.4	0.0002	0.0000	0.0002	0.0005	0.0002
Neglected tropical diseases and malaria	A.4	0.0146	0.0485	0.0630	0.0070	-0.0560
Malaria	A.4.1	0.0036	0.0005	0.0041	0.0012	-0.0029
Chagas disease	A.4.2	0.0000	0.0000	0.0000	0.0000	0.0000
Leishmaniasis	A.4.3	0.0000	0.0000	0.0000	0.0000	0.0000
African trypanosomiasis	A.4.4	0.0000	0.0000	0.0000	0.0000	0.0000
Schistosomiasis	A.4.5	0.0041	0.0023	0.0065	0.0021	-0.0044
Cysticercosis	A.4.6	0.0002	-0.0027	-0.0025	0.0001	0.0026
Cystic echinococcosis	A.4.7	0.0002	0.0000	0.0002	0.0001	-0.0001
Lymphatic filariasis	A.4.8	0.0000	0.0000	0.0000	0.0000	0.0000
Onchocerciasis	A.4.9	0.0000	0.0000	0.0000	0.0000	0.0000
Trachoma	A.4.10	0.0000	0.0009	0.0009	0.0000	-0.0009
Dengue	A.4.11	0.0002	-0.0003	-0.0001	0.0001	0.0002
Yellow fever	A.4.12	0.0000	0.0000	0.0000	0.0000	0.0000
Rabies	A.4.13	0.0038	0.0000	0.0038	0.0016	-0.0023
Intestinal nematode infections	A.4.14	0.0007	0.0200	0.0207	0.0007	-0.0200
Food-borne trematodiases	A.4.15	0.0000	0.0242	0.0242	0.0000	-0.0242
Leprosy	A.4.16	0.0000	0.0000	0.0000	0.0000	0.0000
Ebola	A.4.17	0.0000	0.0000	0.0000	0.0000	0.0000
Zika virus	A.4.18	0.0000	0.0000	0.0000	0.0000	0.0000
Guinea worm disease	A.4.19	0.0000	0.0000	0.0000	0.0000	0.0000
Other neglected tropical diseases	A.4.20	0.0017	0.0035	0.0052	0.0011	-0.0041
Other infectious diseases	A.5	0.1859	0.0072	0.1931	0.2368	0.0437
Meningitis	A.5.1	0.0299	0.0020	0.0319	0.0460	0.0142
Encephalitis	A.5.2	0.0035	0.0014	0.0049	0.0056	0.0007
Diphtheria	A.5.3	0.0004	0.0000	0.0004	0.0004	0.0000

Causes	Level	ΔHALE			ΔLE	ΔLED
		Mortality Effect	Disability Effect	Total Effect	Total Effect	
Whooping cough	A.5.4	0.0177	0.0004	0.0181	0.0533	0.0352
Tetanus	A.5.5	0.0209	0.0000	0.0210	0.0395	0.0186
Measles	A.5.6	0.0356	0.0003	0.0359	0.0531	0.0172
Varicella and herpes zoster	A.5.7	0.0058	0.0000	0.0058	0.0027	-0.0031
Acute hepatitis	A.5.8	0.0631	0.0001	0.0632	0.0312	-0.0320
Other unspecified infectious diseases	A.5.9	0.0090	0.0030	0.0120	0.0052	-0.0069
Maternal and neonatal disorders	A.6	0.0839	-0.0143	0.0696	0.0891	0.0194
Maternal disorders	A.6.1	0.0830	0.0027	0.0857	0.0111	-0.0747
Neonatal disorders	A.6.2	0.0009	-0.0170	-0.0161	0.0780	0.0941
Nutritional deficiencies	A.7	0.0334	0.0687	0.1021	0.0581	-0.0440
Protein-energy malnutrition	A.7.1	0.0325	-0.0036	0.0289	0.0571	0.0282
Iodine deficiency	A.7.2	0.0000	0.0019	0.0019	0.0000	-0.0019
Vitamin A deficiency	A.7.3	0.0000	0.0020	0.0020	0.0000	-0.0020
Dietary iron deficiency	A.7.4	0.0000	0.0665	0.0665	0.0000	-0.0665
Other nutritional deficiencies	A.7.5	0.0009	0.0020	0.0029	0.0011	-0.0018
**Non-communicable diseases**	B	1.4347	0.1446	1.5793	1.5873	0.0081
Neoplasms	B.1	-0.1800	-0.0048	-0.1848	0.1231	0.3079
Lip and oral cavity cancer	B.1.1	0.0004	0.0000	0.0004	0.0010	0.0007
Nasopharynx cancer	B.1.2	0.0141	-0.0001	0.0140	0.0091	-0.0049
Other pharynx cancer	B.1.3	0.0009	0.0000	0.0009	0.0007	-0.0002
Esophageal cancer	B.1.4	0.0032	0.0000	0.0031	0.0139	0.0107
Stomach cancer	B.1.5	0.0570	0.0006	0.0576	0.0816	0.0240
Colon and rectum cancer	B.1.6	-0.0263	-0.0010	-0.0273	-0.0023	0.0251
Liver cancer	B.1.7	-0.0121	0.0001	-0.0120	0.0102	0.0221
Gallbladder and biliary tract cancer	B.1.8	0.0026	0.0000	0.0026	0.0020	-0.0006
Pancreatic cancer	B.1.9	-0.0135	-0.0001	-0.0136	-0.0062	0.0074
Larynx cancer	B.1.10	0.0037	0.0000	0.0038	0.0031	-0.0006
Tracheal, bronchus, and lung cancer	B.1.11	-0.0773	-0.0009	-0.0782	-0.0264	0.0518
Malignant skin melanoma	B.1.12	-0.0004	0.0000	-0.0004	0.0004	0.0008
Non-melanoma skin cancer	B.1.13	-0.0012	0.0000	-0.0013	0.0003	0.0015
Breast cancer	B.1.14	-0.0379	-0.0009	-0.0388	0.0012	0.0400
Cervical cancer	B.1.15	-0.0162	0.0000	-0.0162	0.0082	0.0244
Uterine cancer	B.1.16	-0.0053	-0.0002	-0.0055	0.0017	0.0073
Ovarian cancer	B.1.17	-0.0093	-0.0001	-0.0095	-0.0024	0.0071
Prostate cancer	B.1.18	-0.0017	-0.0007	-0.0024	-0.0005	0.0019
Testicular cancer	B.1.19	-0.0005	-0.0001	-0.0005	0.0000	0.0005
Kidney cancer	B.1.20	-0.0007	-0.0001	-0.0008	-0.0004	0.0003
Bladder cancer	B.1.21	0.0031	-0.0002	0.0029	0.0019	-0.0010
Brain and central nervous system cancer	B.1.22	-0.0189	-0.0001	-0.0190	-0.0005	0.0185
Thyroid cancer	B.1.23	-0.0013	-0.0001	-0.0014	0.0006	0.0019
Mesothelioma	B.1.24	0.0002	0.0000	0.0002	0.0003	0.0001
Hodgkin lymphoma	B.1.25	0.0088	0.0000	0.0088	0.0031	-0.0057

Causes	Level	ΔHALE			ΔLE	ΔLED
		Mortality Effect	Disability Effect	Total Effect	Total Effect	
Non-Hodgkin lymphoma	B.1.26	-0.0133	-0.0003	-0.0135	0.0000	0.0135
Multiple myeloma	B.1.27	-0.0056	0.0000	-0.0057	0.0000	0.0057
Leukemia	B.1.28	-0.0234	0.0001	-0.0233	0.0156	0.0390
Other malignant neoplasms	B.1.29	-0.0080	-0.0005	-0.0085	0.0072	0.0157
Other neoplasms	B.1.30	-0.0010	0.0000	-0.0010	-0.0002	0.0008
Cardiovascular diseases	B.2	0.6131	-0.0112	0.6019	0.6348	0.0329
Rheumatic heart disease	B.2.1	0.1844	0.0019	0.1864	0.1018	-0.0846
Ischemic heart disease	B.2.2	0.0206	-0.0036	0.0170	0.1038	0.0868
Stroke	B.2.3	0.1553	-0.0128	0.1426	0.2647	0.1222
Hypertensive heart disease	B.2.4	0.2587	0.0013	0.2600	0.1525	-0.1076
Non-rheumatic valvular heart disease	B.2.5	0.0008	-0.0001	0.0008	0.0008	0.0000
Cardiomyopathy and myocarditis	B.2.6	-0.0081	0.0000	-0.0081	0.0013	0.0094
Atrial fibrillation and flutter	B.2.8	0.0003	0.0026	0.0029	0.0015	-0.0015
Aortic aneurysm	B.2.9	0.0002	0.0000	0.0002	0.0006	0.0004
Peripheral artery disease	B.2.10	-0.0002	-0.0001	-0.0003	0.0000	0.0002
Endocarditis	B.2.11	-0.0047	0.0000	-0.0047	0.0001	0.0048
Other cardiovascular and circulatory diseases	B.2.12	0.0056	-0.0005	0.0051	0.0077	0.0026
Chronic respiratory diseases	B.3	0.8253	-0.0039	0.8214	0.5520	-0.2694
Chronic obstructive pulmonary disease	B.3.1	0.7761	-0.0066	0.7695	0.5228	-0.2466
Pneumoconiosis	B.3.2	0.0097	-0.0037	0.0059	0.0061	0.0002
Asthma	B.3.3	0.0409	0.0075	0.0484	0.0222	-0.0262
Interstitial lung disease and pulmonary sarcoidosis	B.3.4	0.0008	0.0003	0.0011	0.0007	-0.0004
Other chronic respiratory diseases	B.3.5	-0.0022	-0.0014	-0.0035	0.0001	0.0037
Digestive diseases	B.4	0.3044	0.0220	0.3264	0.1755	-0.1509
Cirrhosis and other chronic liver diseases	B.4.1	0.1444	0.0011	0.1455	0.0767	-0.0688
Upper digestive system diseases	B.4.2	0.0858	0.0087	0.0944	0.0466	-0.0478
Appendicitis	B.4.3	0.0070	0.0001	0.0070	0.0029	-0.0042
Paralytic ileus and intestinal obstruction	B.4.4	0.0147	-0.0002	0.0145	0.0149	0.0004
Inguinal, femoral, and abdominal hernia	B.4.5	0.0014	0.0009	0.0023	0.0022	0.0000
Inflammatory bowel disease	B.4.6	0.0026	-0.0014	0.0012	0.0022	0.0010
Vascular intestinal disorders	B.4.7	0.0006	0.0000	0.0006	0.0006	-0.0001
Gallbladder and biliary diseases	B.4.8	0.0147	0.0121	0.0268	0.0100	-0.0168
Pancreatitis	B.4.9	0.0086	0.0002	0.0088	0.0037	-0.0051
Other digestive diseases	B.4.10	0.0247	0.0005	0.0251	0.0156	-0.0096
Neurological disorders	B.5	0.0108	-0.0350	-0.0243	0.0157	0.0399
Alzheimer's disease and other dementias	B.5.1	0.0126	-0.0165	-0.0038	0.0057	0.0096
Parkinson's disease	B.5.2	0.0023	-0.0057	-0.0035	0.0038	0.0073
Idiopathic epilepsy	B.5.3	0.0016	-0.0147	-0.0131	0.0059	0.0189
Multiple sclerosis	B.5.4	-0.0020	0.0000	-0.0020	0.0000	0.0020
Motor neuron disease	B.5.5	-0.0016	0.0000	-0.0016	0.0000	0.0015
Headache disorders	B.5.6	0.0000	0.0044	0.0044	0.0000	-0.0044
Other neurological disorders	B.5.7	-0.0022	-0.0025	-0.0047	0.0003	0.0050

Causes	Level	ΔHALE			ΔLE	ΔLED
		Mortality Effect	Disability Effect	Total Effect	Total Effect	
Mental disorders	B.6	-0.0001	-0.0096	-0.0098	0.0000	0.0098
Schizophrenia	B.6.1	0.0000	-0.0007	-0.0007	0.0000	0.0007
Depressive disorders	B.6.2	0.0000	0.0014	0.0014	0.0000	-0.0014
Bipolar disorder	B.6.3	0.0000	-0.0001	-0.0001	0.0000	0.0001
Anxiety disorders	B.6.4	0.0000	-0.0086	-0.0086	0.0000	0.0086
Eating disorders	B.6.5	-0.0001	-0.0007	-0.0008	0.0000	0.0008
Autism spectrum disorders	B.6.6	0.0000	-0.0007	-0.0007	0.0000	0.0007
Attention-deficit/hyperactivity disorder	B.6.7	0.0000	-0.0013	-0.0013	0.0000	0.0013
Conduct disorder	B.6.8	0.0000	-0.0001	-0.0001	0.0000	0.0001
Idiopathic developmental intellectual disability	B.6.9	0.0000	0.0015	0.0015	0.0000	-0.0015
Other mental disorders	B.6.10	0.0000	-0.0003	-0.0003	0.0000	0.0003
Substance use disorders	B.7	-0.1063	0.0079	-0.0983	-0.0055	0.0928
Alcohol use disorders	B.7.1	-0.0015	-0.0008	-0.0023	0.0005	0.0028
Drug use disorders	B.7.2	-0.1048	0.0087	-0.0960	-0.0060	0.0900
Diabetes and kidney diseases	B.8	-0.0165	-0.0074	-0.0239	0.0259	0.0498
Diabetes mellitus	B.8.1	-0.0318	-0.0157	-0.0475	-0.0026	0.0449
Chronic kidney disease	B.8.2	-0.0109	0.0083	-0.0026	0.0190	0.0216
Acute glomerulonephritis	B.8.3	0.0262	0.0000	0.0261	0.0095	-0.0166
Skin and subcutaneous diseases	B.9	0.0045	-0.0017	0.0028	0.0030	0.0002
Dermatitis	B.9.1	0.0000	0.0003	0.0003	0.0000	-0.0003
Psoriasis	B.9.2	0.0000	0.0030	0.0030	0.0000	-0.0030
Bacterial skin diseases	B.9.3	0.0098	0.0000	0.0098	0.0046	-0.0052
Scabies	B.9.4	0.0000	0.0006	0.0006	0.0000	-0.0006
Fungal skin diseases	B.9.5	0.0000	0.0001	0.0001	0.0000	-0.0001
Viral skin diseases	B.9.6	0.0000	-0.0001	-0.0001	0.0000	0.0001
Acne vulgaris	B.9.7	0.0000	-0.0042	-0.0042	0.0000	0.0042
Alopecia areata	B.9.8	0.0000	0.0000	0.0000	0.0000	0.0000
Pruritus	B.9.9	0.0000	-0.0002	-0.0002	0.0000	0.0002
Urticaria	B.9.10	0.0000	0.0000	0.0000	0.0000	0.0000
Decubitus ulcer	B.9.11	-0.0025	-0.0001	-0.0026	-0.0007	0.0019
Other skin and subcutaneous diseases	B.9.12	-0.0028	-0.0011	-0.0038	-0.0009	0.0029
Sense organ diseases	B.10	0.0000	-0.0270	-0.0270	0.0000	0.0270
Blindness and vision loss	B.10.1	0.0000	-0.0243	-0.0243	0.0000	0.0243
Age-related and other hearing loss	B.10.2	0.0000	-0.0021	-0.0021	0.0000	0.0021
Other sense organ diseases	B.10.3	0.0000	-0.0006	-0.0006	0.0000	0.0006
Musculoskeletal disorders	B.11	-0.0179	0.1421	0.1242	-0.0012	-0.1254
Rheumatoid arthritis	B.11.1	0.0007	-0.0008	-0.0001	0.0008	0.0010
Osteoarthritis	B.11.2	0.0000	-0.0003	-0.0003	0.0000	0.0003
Low back pain	B.11.3	0.0000	0.1752	0.1752	0.0000	-0.1752
Neck pain	B.11.4	0.0000	-0.0030	-0.0030	0.0000	0.0030
Gout	B.11.5	0.0000	0.0009	0.0009	0.0000	-0.0009
Other musculoskeletal disorders	B.11.6	-0.0186	-0.0299	-0.0485	-0.0020	0.0465

Causes	Level	ΔHALE			ΔLE	ΔLED
		Mortality Effect	Disability Effect	Total Effect	Total Effect	
Other non-communicable diseases	B.12	-0.0026	0.0733	0.0706	0.0642	-0.0065
Congenital birth defects	B.12.1	-0.0078	0.0017	-0.0060	0.0516	0.0576
Urinary diseases and male infertility	B.12.2	-0.0003	0.0038	0.0036	0.0033	-0.0002
Gynecological diseases	B.12.3	0.0000	0.0004	0.0004	0.0001	-0.0003
Hemoglobinopathies and hemolytic anemias	B.12.4	0.0063	0.0223	0.0286	0.0061	-0.0225
Endocrine, metabolic, blood, and immune disorders	B.12.5	-0.0009	0.0045	0.0035	0.0034	-0.0002
Oral disorders	B.12.6	0.0000	0.0406	0.0406	0.0000	-0.0406
**Injuries**	C	0.3761	0.0064	0.3825	0.3204	-0.0621
Transport injuries	C.1	-0.3562	-0.0033	-0.3595	0.0076	0.3671
Road injuries	C.1.1	-0.3736	-0.0054	-0.3790	0.0006	0.3796
Other transport injuries	C.1.2	0.0175	0.0020	0.0195	0.0070	-0.0125
Unintentional injuries	C.2	0.2916	0.0067	0.2983	0.1893	-0.1091
Falls	C.2.1	0.0540	-0.0013	0.0527	0.0192	-0.0335
Drowning	C.2.2	0.1073	0.0003	0.1076	0.0971	-0.0105
Fire, heat, and hot substances	C.2.3	0.0241	0.0024	0.0265	0.0138	-0.0127
Poisonings	C.2.4	-0.0037	0.0003	-0.0034	0.0054	0.0088
Exposure to mechanical forces	C.2.5	0.0511	-0.0002	0.0509	0.0015	-0.0494
Adverse effects of medical treatment	C.2.6	0.0123	0.0001	0.0123	0.0099	-0.0024
Animal contact	C.2.7	0.0146	0.0013	0.0159	0.0063	-0.0096
Foreign body	C.2.8	-0.0052	0.0022	-0.0031	0.0225	0.0255
Environmental heat and cold exposure	C.2.9	0.0250	0.0014	0.0263	0.0098	-0.0165
Exposure to forces of nature	C.2.10	-0.0026	-0.0004	-0.0030	-0.0004	0.0026
Other unintentional injuries	C.2.11	0.0148	0.0007	0.0156	0.0043	-0.0113
Self-harm and interpersonal violence	C.3	0.4407	0.0030	0.4437	0.1235	-0.3202
Self-harm	C.3.1	0.3162	0.0019	0.3182	0.1004	-0.2177
Interpersonal violence	C.3.2	0.1014	0.0011	0.1025	0.0211	-0.0814
Conflict and terrorism	C.3.3	-0.0042	0.0000	-0.0042	-0.0006	0.0037
Executions and police conflict	C.3.4	0.0273	0.0000	0.0273	0.0026	-0.0247

Table S4 | Cause-specific effects of mortality and disability (years) on changes in life expectancy (LE) and health-adjusted life expectancy (HALE) from 2000 to 2009.

Causes	Level	ΔHALE			ΔLE	ΔLED
		Mortality Effect	Disability Effect	Total Effect	Total Effect	
**Communicable, maternal, neonatal, and nutritional diseases**	A	0.4342	0.0821	0.5163	1.2765	0.7603
HIV/AIDS and sexually transmitted infections	A.1	-0.0109	-0.0011	-0.0121	-0.0094	0.0026
HIV/AIDS	A.1.1	-0.0112	-0.0017	-0.0129	-0.0087	0.0042
Sexually transmitted infections excluding HIV	A.1.2	0.0003	0.0005	0.0008	-0.0007	-0.0015
Respiratory infections and tuberculosis	A.2	0.3312	0.0150	0.3462	0.6761	0.3298
Tuberculosis	A.2.1	0.1367	0.0110	0.1476	0.1202	-0.0274
Lower respiratory infections	A.2.2	0.1766	0.0013	0.1779	0.5388	0.3609
Upper respiratory infections	A.2.3	0.0179	-0.0002	0.0177	0.0170	-0.0007
Otitis media	A.2.4	0.0000	0.0029	0.0030	0.0000	-0.0029
Enteric infections	A.3	0.0219	0.0024	0.0243	0.0985	0.0742
Diarrheal diseases	A.3.1	0.0201	0.0024	0.0225	0.0969	0.0744
Typhoid and paratyphoid	A.3.2	0.0012	0.0000	0.0012	0.0008	-0.0004
Invasive Non-typhoidal Salmonella (iNTS)	A.3.3	0.0004	0.0000	0.0004	0.0004	0.0000
Other intestinal infectious diseases	A.3.4	0.0002	0.0000	0.0002	0.0004	0.0002
Neglected tropical diseases and malaria	A.4	-0.0008	0.0363	0.0355	0.0000	-0.0355
Malaria	A.4.1	0.0003	0.0001	0.0004	0.0003	-0.0001
Chagas disease	A.4.2	0.0000	0.0000	0.0000	0.0000	0.0000
Leishmaniasis	A.4.3	0.0000	0.0000	0.0000	0.0000	0.0000
African trypanosomiasis	A.4.4	0.0000	0.0000	0.0000	0.0000	0.0000
Schistosomiasis	A.4.5	0.0018	0.0011	0.0029	0.0014	-0.0016
Cysticercosis	A.4.6	0.0001	0.0046	0.0047	0.0001	-0.0046
Cystic echinococcosis	A.4.7	0.0001	0.0000	0.0001	0.0000	0.0000
Lymphatic filariasis	A.4.8	0.0000	0.0000	0.0000	0.0000	0.0000
Onchocerciasis	A.4.9	0.0000	0.0000	0.0000	0.0000	0.0000
Trachoma	A.4.10	0.0000	0.0018	0.0018	0.0000	-0.0018
Dengue	A.4.11	0.0000	-0.0010	-0.0009	0.0000	0.0010
Yellow fever	A.4.12	0.0000	0.0000	0.0000	0.0000	0.0000
Rabies	A.4.13	-0.0034	0.0000	-0.0034	-0.0025	0.0009
Intestinal nematode infections	A.4.14	0.0002	0.0167	0.0170	0.0003	-0.0166
Food-borne trematodiases	A.4.15	0.0000	0.0096	0.0096	0.0000	-0.0096
Leprosy	A.4.16	0.0000	0.0000	0.0000	0.0000	0.0000
Ebola	A.4.17	0.0000	0.0000	0.0000	0.0000	0.0000
Zika virus	A.4.18	0.0000	0.0000	0.0000	0.0000	0.0000
Guinea worm disease	A.4.19	0.0000	0.0000	0.0000	0.0000	0.0000
Other neglected tropical diseases	A.4.20	0.0001	0.0033	0.0034	0.0004	-0.0031
Other infectious diseases	A.5	0.0552	0.0067	0.0619	0.1142	0.0523
Meningitis	A.5.1	0.0152	0.0021	0.0172	0.0289	0.0117
Encephalitis	A.5.2	0.0020	0.0008	0.0028	0.0054	0.0026
Diphtheria	A.5.3	0.0000	0.0000	0.0000	0.0001	0.0001

Causes	Level	ΔHALE			ΔLE	ΔLED
		Mortality Effect	Disability Effect	Total Effect	Total Effect	
Whooping cough	A.5.4	0.0035	0.0001	0.0036	0.0172	0.0136
Tetanus	A.5.5	0.0033	0.0000	0.0033	0.0111	0.0078
Measles	A.5.6	0.0061	0.0003	0.0064	0.0196	0.0132
Varicella and herpes zoster	A.5.7	0.0025	0.0000	0.0025	0.0027	0.0002
Acute hepatitis	A.5.8	0.0187	0.0005	0.0191	0.0196	0.0005
Other unspecified infectious diseases	A.5.9	0.0040	0.0029	0.0069	0.0096	0.0027
Maternal and neonatal disorders	A.6	0.0273	-0.0359	-0.0087	0.3613	0.3700
Maternal disorders	A.6.1	0.0233	0.0007	0.0241	0.0158	-0.0082
Neonatal disorders	A.6.2	0.0040	-0.0367	-0.0327	0.3455	0.3782
Nutritional deficiencies	A.7	0.0102	0.0588	0.0690	0.0359	-0.0331
Protein-energy malnutrition	A.7.1	0.0118	-0.0046	0.0073	0.0357	0.0284
Iodine deficiency	A.7.2	0.0000	-0.0020	-0.0020	0.0000	0.0020
Vitamin A deficiency	A.7.3	0.0000	0.0012	0.0012	0.0000	-0.0012
Dietary iron deficiency	A.7.4	0.0000	0.0683	0.0683	0.0000	-0.0683
Other nutritional deficiencies	A.7.5	-0.0016	-0.0041	-0.0057	0.0002	0.0059
**Non-communicable diseases**	B	2.2806	0.0502	2.3307	2.0617	-0.2690
Neoplasms	B.1	0.3577	-0.0203	0.3374	0.3586	0.0212
Lip and oral cavity cancer	B.1.1	-0.0057	-0.0003	-0.0060	-0.0047	0.0013
Nasopharynx cancer	B.1.2	0.0185	-0.0007	0.0179	0.0144	-0.0035
Other pharynx cancer	B.1.3	0.0009	0.0000	0.0008	0.0006	-0.0002
Esophageal cancer	B.1.4	0.0715	0.0006	0.0721	0.0654	-0.0067
Stomach cancer	B.1.5	0.0650	-0.0014	0.0637	0.0633	-0.0004
Colon and rectum cancer	B.1.6	-0.0603	-0.0063	-0.0666	-0.0446	0.0220
Liver cancer	B.1.7	0.3406	0.0032	0.3439	0.2822	-0.0616
Gallbladder and biliary tract cancer	B.1.8	-0.0107	-0.0002	-0.0109	-0.0078	0.0031
Pancreatic cancer	B.1.9	-0.0248	-0.0003	-0.0251	-0.0187	0.0064
Larynx cancer	B.1.10	0.0028	-0.0003	0.0026	0.0027	0.0001
Tracheal, bronchus, and lung cancer	B.1.11	-0.0820	-0.0022	-0.0842	-0.0478	0.0364
Malignant skin melanoma	B.1.12	0.0005	-0.0001	0.0003	0.0004	0.0000
Non-melanoma skin cancer	B.1.13	-0.0045	0.0000	-0.0045	-0.0031	0.0015
Breast cancer	B.1.14	0.0043	-0.0031	0.0013	0.0034	0.0021
Cervical cancer	B.1.15	-0.0046	-0.0005	-0.0051	-0.0039	0.0012
Uterine cancer	B.1.16	0.0014	-0.0008	0.0007	0.0012	0.0005
Ovarian cancer	B.1.17	-0.0026	-0.0002	-0.0028	-0.0024	0.0003
Prostate cancer	B.1.18	0.0018	-0.0018	0.0000	0.0019	0.0019
Testicular cancer	B.1.19	0.0005	-0.0001	0.0003	0.0004	0.0000
Kidney cancer	B.1.20	-0.0101	-0.0006	-0.0106	-0.0073	0.0033
Bladder cancer	B.1.21	0.0029	-0.0008	0.0021	0.0024	0.0002
Brain and central nervous system cancer	B.1.22	0.0108	-0.0002	0.0105	0.0126	0.0020
Thyroid cancer	B.1.23	-0.0002	-0.0002	-0.0004	-0.0001	0.0004
Mesothelioma	B.1.24	-0.0008	0.0000	-0.0008	-0.0007	0.0002
Hodgkin lymphoma	B.1.25	0.0043	0.0000	0.0042	0.0034	-0.0008

Causes	Level	ΔHALE			ΔLE	ΔLED
		Mortality Effect	Disability Effect	Total Effect	Total Effect	
Non-Hodgkin lymphoma	B.1.26	-0.0070	-0.0013	-0.0083	-0.0054	0.0029
Multiple myeloma	B.1.27	0.0003	-0.0001	0.0002	0.0002	0.0000
Leukemia	B.1.28	0.0372	0.0002	0.0374	0.0400	0.0026
Other malignant neoplasms	B.1.29	0.0080	-0.0019	0.0061	0.0108	0.0047
Other neoplasms	B.1.30	-0.0005	-0.0009	-0.0014	-0.0003	0.0012
Cardiovascular diseases	B.2	0.4021	-0.0119	0.3902	0.4180	0.0278
Rheumatic heart disease	B.2.1	0.0915	-0.0008	0.0907	0.0677	-0.0230
Ischemic heart disease	B.2.2	-0.4836	0.0017	-0.4820	-0.2674	0.2146
Stroke	B.2.3	0.6466	-0.0122	0.6344	0.5078	-0.1266
Hypertensive heart disease	B.2.4	0.1366	0.0032	0.1398	0.0960	-0.0439
Non-rheumatic valvular heart disease	B.2.5	0.0022	-0.0005	0.0018	0.0015	-0.0003
Cardiomyopathy and myocarditis	B.2.6	-0.0046	0.0000	-0.0046	-0.0004	0.0042
Atrial fibrillation and flutter	B.2.8	0.0029	-0.0025	0.0004	0.0016	0.0012
Aortic aneurysm	B.2.9	0.0005	0.0000	0.0005	0.0003	-0.0002
Peripheral artery disease	B.2.10	-0.0001	0.0011	0.0009	-0.0001	-0.0010
Endocarditis	B.2.11	0.0041	0.0000	0.0041	0.0049	0.0008
Other cardiovascular and circulatory diseases	B.2.12	0.0061	-0.0020	0.0042	0.0061	0.0020
Chronic respiratory diseases	B.3	1.2184	0.0718	1.2902	0.8150	-0.4752
Chronic obstructive pulmonary disease	B.3.1	1.1761	0.0571	1.2331	0.7844	-0.4488
Pneumoconiosis	B.3.2	0.0048	0.0026	0.0074	0.0041	-0.0033
Asthma	B.3.3	0.0411	0.0138	0.0548	0.0285	-0.0263
Interstitial lung disease and pulmonary sarcoidosis	B.3.4	-0.0015	-0.0007	-0.0021	-0.0010	0.0012
Other chronic respiratory diseases	B.3.5	-0.0020	-0.0010	-0.0030	-0.0010	0.0020
Digestive diseases	B.4	0.1758	0.0157	0.1916	0.1497	-0.0419
Cirrhosis and other chronic liver diseases	B.4.1	0.0890	0.0014	0.0904	0.0727	-0.0178
Upper digestive system diseases	B.4.2	0.0357	0.0099	0.0456	0.0281	-0.0175
Appendicitis	B.4.3	0.0041	-0.0002	0.0039	0.0030	-0.0010
Paralytic ileus and intestinal obstruction	B.4.4	0.0060	-0.0001	0.0059	0.0130	0.0071
Inguinal, femoral, and abdominal hernia	B.4.5	0.0011	0.0005	0.0017	0.0021	0.0004
Inflammatory bowel disease	B.4.6	0.0053	-0.0008	0.0045	0.0039	-0.0005
Vascular intestinal disorders	B.4.7	0.0003	0.0000	0.0003	0.0003	-0.0001
Gallbladder and biliary diseases	B.4.8	0.0153	0.0043	0.0197	0.0113	-0.0084
Pancreatitis	B.4.9	0.0036	0.0000	0.0036	0.0028	-0.0008
Other digestive diseases	B.4.10	0.0153	0.0006	0.0159	0.0126	-0.0033
Neurological disorders	B.5	0.0043	-0.0202	-0.0159	0.0116	0.0275
Alzheimer's disease and other dementias	B.5.1	-0.0193	-0.0037	-0.0229	-0.0108	0.0122
Parkinson's disease	B.5.2	0.0061	-0.0005	0.0055	0.0040	-0.0015
Idiopathic epilepsy	B.5.3	0.0149	0.0060	0.0209	0.0138	-0.0071
Multiple sclerosis	B.5.4	0.0008	0.0000	0.0008	0.0006	-0.0002
Motor neuron disease	B.5.5	0.0018	0.0000	0.0018	0.0031	0.0012
Headache disorders	B.5.6	0.0000	-0.0213	-0.0213	0.0000	0.0213
Other neurological disorders	B.5.7	-0.0001	-0.0007	-0.0007	0.0009	0.0016

Causes	Level	ΔHALE			ΔLE	ΔLED
		Mortality Effect	Disability Effect	Total Effect	Total Effect	
Mental disorders	B.6	0.0000	0.0444	0.0444	0.0000	-0.0444
Schizophrenia	B.6.1	0.0000	-0.0018	-0.0018	0.0000	0.0018
Depressive disorders	B.6.2	0.0000	0.0203	0.0203	0.0000	-0.0203
Bipolar disorder	B.6.3	0.0000	-0.0001	-0.0001	0.0000	0.0001
Anxiety disorders	B.6.4	0.0000	0.0262	0.0262	0.0000	-0.0262
Eating disorders	B.6.5	0.0000	-0.0022	-0.0022	0.0000	0.0022
Autism spectrum disorders	B.6.6	0.0000	-0.0004	-0.0004	0.0000	0.0004
Attention-deficit/hyperactivity disorder	B.6.7	0.0000	0.0001	0.0001	0.0000	-0.0001
Conduct disorder	B.6.8	0.0000	-0.0012	-0.0012	0.0000	0.0012
Idiopathic developmental intellectual disability	B.6.9	0.0000	0.0036	0.0036	0.0000	-0.0036
Other mental disorders	B.6.10	0.0000	-0.0001	-0.0001	0.0000	0.0001
Substance use disorders	B.7	0.0554	0.0133	0.0687	0.0394	-0.0293
Alcohol use disorders	B.7.1	-0.0099	0.0072	-0.0027	-0.0079	-0.0052
Drug use disorders	B.7.2	0.0653	0.0061	0.0713	0.0472	-0.0241
Diabetes and kidney diseases	B.8	0.0207	-0.0865	-0.0658	0.0264	0.0923
Diabetes mellitus	B.8.1	0.0033	-0.0606	-0.0572	0.0081	0.0653
Chronic kidney disease	B.8.2	0.0126	-0.0260	-0.0134	0.0143	0.0278
Acute glomerulonephritis	B.8.3	0.0048	0.0000	0.0048	0.0040	-0.0008
Skin and subcutaneous diseases	B.9	0.0068	-0.0034	0.0033	0.0056	0.0023
Dermatitis	B.9.1	0.0000	-0.0008	-0.0008	0.0000	0.0008
Psoriasis	B.9.2	0.0000	0.0035	0.0035	0.0000	-0.0035
Bacterial skin diseases	B.9.3	0.0070	0.0001	0.0071	0.0053	-0.0017
Scabies	B.9.4	0.0000	0.0004	0.0004	0.0000	-0.0004
Fungal skin diseases	B.9.5	0.0000	0.0003	0.0003	0.0000	-0.0003
Viral skin diseases	B.9.6	0.0000	-0.0001	-0.0001	0.0000	0.0001
Acne vulgaris	B.9.7	0.0000	-0.0049	-0.0049	0.0000	0.0049
Alopecia areata	B.9.8	0.0000	0.0000	0.0000	0.0000	0.0000
Pruritus	B.9.9	0.0000	-0.0003	-0.0003	0.0000	0.0003
Urticaria	B.9.10	0.0000	0.0000	0.0000	0.0000	0.0000
Decubitus ulcer	B.9.11	-0.0016	-0.0001	-0.0017	-0.0009	0.0008
Other skin and subcutaneous diseases	B.9.12	0.0013	-0.0015	-0.0001	0.0011	0.0013
Sense organ diseases	B.10	0.0000	0.0381	0.0381	0.0000	-0.0381
Blindness and vision loss	B.10.1	0.0000	0.0318	0.0318	0.0000	-0.0318
Age-related and other hearing loss	B.10.2	0.0000	0.0068	0.0068	0.0000	-0.0068
Other sense organ diseases	B.10.3	0.0000	-0.0004	-0.0004	0.0000	0.0004
Musculoskeletal disorders	B.11	-0.0019	-0.0383	-0.0402	-0.0013	0.0390
Rheumatoid arthritis	B.11.1	-0.0021	-0.0015	-0.0036	-0.0014	0.0022
Osteoarthritis	B.11.2	0.0000	-0.0170	-0.0170	0.0000	0.0170
Low back pain	B.11.3	0.0000	0.0217	0.0217	0.0000	-0.0217
Neck pain	B.11.4	0.0000	-0.0047	-0.0047	0.0000	0.0047
Gout	B.11.5	0.0000	-0.0038	-0.0038	0.0000	0.0038
Other musculoskeletal disorders	B.11.6	0.0002	-0.0330	-0.0328	0.0001	0.0329

Causes	Level	ΔHALE			ΔLE	ΔLED
		Mortality Effect	Disability Effect	Total Effect	Total Effect	
Other non-communicable diseases	B.12	0.0414	0.0475	0.0888	0.2388	0.1500
Congenital birth defects	B.12.1	0.0271	0.0040	0.0311	0.2157	0.1846
Urinary diseases and male infertility	B.12.2	0.0071	0.0003	0.0074	0.0056	-0.0018
Gynecological diseases	B.12.3	-0.0007	0.0449	0.0442	-0.0006	-0.0448
Hemoglobinopathies and hemolytic anemias	B.12.4	0.0047	0.0215	0.0262	0.0071	-0.0191
Endocrine, metabolic, blood, and immune disorders	B.12.5	0.0032	0.0038	0.0070	0.0056	-0.0015
Oral disorders	B.12.6	0.0000	-0.0271	-0.0271	0.0000	0.0271
**Injuries**	C	0.2456	0.0724	0.3180	0.3139	-0.0041
Transport injuries	C.1	0.0009	-0.0162	-0.0153	0.0140	0.0292
Road injuries	C.1.1	-0.0094	-0.0180	-0.0274	0.0051	0.0326
Other transport injuries	C.1.2	0.0104	0.0018	0.0122	0.0089	-0.0033
Unintentional injuries	C.2	0.0301	0.0841	0.1142	0.1421	0.0279
Falls	C.2.1	-0.0519	0.0708	0.0189	-0.0223	-0.0411
Drowning	C.2.2	0.0983	0.0008	0.0991	0.1203	0.0212
Fire, heat, and hot substances	C.2.3	0.0077	0.0004	0.0081	0.0099	0.0018
Poisonings	C.2.4	-0.0120	0.0001	-0.0119	-0.0004	0.0115
Exposure to mechanical forces	C.2.5	-0.0142	-0.0029	-0.0171	0.0015	0.0186
Adverse effects of medical treatment	C.2.6	0.0082	0.0001	0.0083	0.0111	0.0028
Animal contact	C.2.7	0.0078	0.0022	0.0100	0.0077	-0.0024
Foreign body	C.2.8	0.0021	0.0121	0.0142	0.0231	0.0089
Environmental heat and cold exposure	C.2.9	0.0084	0.0010	0.0094	0.0083	-0.0010
Exposure to forces of nature	C.2.10	0.0003	-0.0018	-0.0015	0.0002	0.0018
Other unintentional injuries	C.2.11	-0.0246	0.0013	-0.0233	-0.0172	0.0061
Self-harm and interpersonal violence	C.3	0.2145	0.0046	0.2191	0.1577	-0.0613
Self-harm	C.3.1	0.1771	0.0022	0.1793	0.1247	-0.0546
Interpersonal violence	C.3.2	0.0324	0.0024	0.0348	0.0296	-0.0053
Conflict and terrorism	C.3.3	-0.0005	0.0000	-0.0005	-0.0004	0.0001
Executions and police conflict	C.3.4	0.0055	0.0000	0.0055	0.0039	-0.0016

Table S5 | Cause-specific effects of mortality and disability (years) on changes in life expectancy (LE) and health-adjusted life expectancy (HALE) from 2010 to 2019.

Causes	Level	ΔHALE			ΔLE	ΔLED
		Mortality Effect	Disability Effect	Total Effect	Total Effect	
**Communicable, maternal, neonatal, and nutritional diseases**	A	0.1174	-0.0094	0.1080	0.4863	0.3783
HIV/AIDS and sexually transmitted infections	A.1	-0.0172	-0.0012	-0.0184	-0.0144	0.0040
HIV/AIDS	A.1.1	-0.0174	-0.0008	-0.0182	-0.0183	-0.0001
Sexually transmitted infections excluding HIV	A.1.2	0.0002	-0.0004	-0.0002	0.0039	0.0041
Respiratory infections and tuberculosis	A.2	0.1098	0.0098	0.1195	0.2166	0.0970
Tuberculosis	A.2.1	0.0331	0.0081	0.0411	0.0415	0.0004
Lower respiratory infections	A.2.2	0.0745	0.0005	0.0750	0.1723	0.0973
Upper respiratory infections	A.2.3	0.0022	-0.0005	0.0017	0.0028	0.0011
Otitis media	A.2.4	0.0000	0.0017	0.0017	0.0000	-0.0017
Enteric infections	A.3	0.0042	-0.0090	-0.0048	0.0149	0.0197
Diarrheal diseases	A.3.1	0.0035	-0.0090	-0.0056	0.0139	0.0195
Typhoid and paratyphoid	A.3.2	0.0005	0.0000	0.0006	0.0007	0.0001
Invasive Non-typhoidal Salmonella (iNTS)	A.3.3	0.0002	0.0000	0.0002	0.0002	0.0000
Other intestinal infectious diseases	A.3.4	0.0001	0.0000	0.0001	0.0001	0.0001
Neglected tropical diseases and malaria	A.4	0.0029	0.0072	0.0101	0.0039	-0.0061
Malaria	A.4.1	0.0000	0.0000	0.0001	0.0000	0.0000
Chagas disease	A.4.2	0.0000	0.0000	0.0000	0.0000	0.0000
Leishmaniasis	A.4.3	0.0000	0.0000	0.0000	0.0000	0.0000
African trypanosomiasis	A.4.4	0.0000	0.0000	0.0000	0.0000	0.0000
Schistosomiasis	A.4.5	0.0003	0.0005	0.0008	0.0004	-0.0005
Cysticercosis	A.4.6	0.0000	0.0034	0.0034	0.0000	-0.0034
Cystic echinococcosis	A.4.7	0.0000	0.0000	0.0000	0.0000	0.0000
Lymphatic filariasis	A.4.8	0.0000	0.0000	0.0000	0.0000	0.0000
Onchocerciasis	A.4.9	0.0000	0.0000	0.0000	0.0000	0.0000
Trachoma	A.4.10	0.0000	0.0003	0.0003	0.0000	-0.0003
Dengue	A.4.11	0.0000	-0.0008	-0.0008	0.0000	0.0008
Yellow fever	A.4.12	0.0000	0.0000	0.0000	0.0000	0.0000
Rabies	A.4.13	0.0022	0.0000	0.0022	0.0031	0.0008
Intestinal nematode infections	A.4.14	0.0000	0.0017	0.0017	0.0001	-0.0016
Food-borne trematodiases	A.4.15	0.0000	0.0013	0.0013	0.0000	-0.0013
Leprosy	A.4.16	0.0000	0.0000	0.0000	0.0000	0.0000
Ebola	A.4.17	0.0000	0.0000	0.0000	0.0000	0.0000
Zika virus	A.4.18	0.0000	0.0000	0.0000	0.0000	0.0000
Guinea worm disease	A.4.19	0.0000	0.0000	0.0000	0.0000	0.0000
Other neglected tropical diseases	A.4.20	0.0003	0.0009	0.0012	0.0004	-0.0007
Other infectious diseases	A.5	0.0104	0.0027	0.0132	0.0366	0.0235
Meningitis	A.5.1	0.0038	0.0005	0.0043	0.0116	0.0073
Encephalitis	A.5.2	0.0020	0.0007	0.0028	0.0068	0.0041
Diphtheria	A.5.3	0.0000	0.0000	0.0000	0.0001	0.0000

Causes	Level	ΔHALE			ΔLE	ΔLED
		Mortality Effect	Disability Effect	Total Effect	Total Effect	
Whooping cough	A.5.4	0.0021	0.0004	0.0026	0.0136	0.0110
Tetanus	A.5.5	0.0004	0.0000	0.0004	0.0009	0.0006
Measles	A.5.6	-0.0001	-0.0001	-0.0002	-0.0004	-0.0002
Varicella and herpes zoster	A.5.7	0.0005	-0.0001	0.0004	0.0007	0.0003
Acute hepatitis	A.5.8	0.0017	0.0007	0.0024	0.0027	0.0003
Other unspecified infectious diseases	A.5.9	-0.0001	0.0006	0.0004	0.0006	0.0001
Maternal and neonatal disorders	A.6	0.0084	-0.0349	-0.0265	0.2248	0.2513
Maternal disorders	A.6.1	0.0055	0.0003	0.0058	0.0044	-0.0014
Neonatal disorders	A.6.2	0.0029	-0.0352	-0.0323	0.2204	0.2527
Nutritional deficiencies	A.7	-0.0011	0.0160	0.0149	0.0038	-0.0111
Protein-energy malnutrition	A.7.1	-0.0013	-0.0044	-0.0057	0.0024	0.0081
Iodine deficiency	A.7.2	0.0000	0.0024	0.0024	0.0000	-0.0024
Vitamin A deficiency	A.7.3	0.0000	0.0001	0.0001	0.0000	-0.0001
Dietary iron deficiency	A.7.4	0.0000	0.0190	0.0190	0.0000	-0.0190
Other nutritional deficiencies	A.7.5	0.0002	-0.0012	-0.0010	0.0014	0.0023
**Non-communicable diseases**	B	1.9068	0.0191	1.9259	2.2493	0.3234
Neoplasms	B.1	0.2699	-0.0122	0.2577	0.3712	0.1135
Lip and oral cavity cancer	B.1.1	0.0010	-0.0001	0.0009	0.0015	0.0005
Nasopharynx cancer	B.1.2	0.0038	-0.0009	0.0029	0.0054	0.0025
Other pharynx cancer	B.1.3	0.0001	0.0000	0.0001	0.0002	0.0001
Esophageal cancer	B.1.4	0.0749	0.0013	0.0762	0.0986	0.0224
Stomach cancer	B.1.5	0.1380	0.0015	0.1395	0.1779	0.0383
Colon and rectum cancer	B.1.6	0.0017	-0.0038	-0.0020	0.0073	0.0093
Liver cancer	B.1.7	-0.0044	-0.0003	-0.0047	-0.0051	-0.0004
Gallbladder and biliary tract cancer	B.1.8	0.0045	0.0001	0.0046	0.0059	0.0013
Pancreatic cancer	B.1.9	-0.0102	-0.0002	-0.0104	-0.0114	-0.0010
Larynx cancer	B.1.10	0.0015	-0.0002	0.0013	0.0021	0.0008
Tracheal, bronchus, and lung cancer	B.1.11	0.0256	-0.0005	0.0251	0.0380	0.0130
Malignant skin melanoma	B.1.12	0.0002	-0.0001	0.0001	0.0003	0.0002
Non-melanoma skin cancer	B.1.13	0.0018	-0.0001	0.0017	0.0022	0.0005
Breast cancer	B.1.14	-0.0007	-0.0027	-0.0034	-0.0001	0.0033
Cervical cancer	B.1.15	0.0027	-0.0001	0.0026	0.0030	0.0005
Uterine cancer	B.1.16	0.0077	0.0006	0.0083	0.0098	0.0015
Ovarian cancer	B.1.17	-0.0031	-0.0002	-0.0033	-0.0040	-0.0007
Prostate cancer	B.1.18	0.0012	-0.0016	-0.0004	0.0022	0.0025
Testicular cancer	B.1.19	-0.0002	-0.0002	-0.0003	-0.0002	0.0002
Kidney cancer	B.1.20	-0.0001	-0.0002	-0.0003	0.0004	0.0008
Bladder cancer	B.1.21	0.0014	-0.0006	0.0009	0.0025	0.0016
Brain and central nervous system cancer	B.1.22	0.0018	-0.0004	0.0015	0.0037	0.0022
Thyroid cancer	B.1.23	0.0006	-0.0001	0.0004	0.0007	0.0003
Mesothelioma	B.1.24	0.0004	0.0000	0.0004	0.0005	0.0001
Hodgkin lymphoma	B.1.25	0.0007	-0.0001	0.0006	0.0009	0.0003

Causes	Level	ΔHALE			ΔLE	ΔLED
		Mortality Effect	Disability Effect	Total Effect	Total Effect	
Non-Hodgkin lymphoma	B.1.26	-0.0004	-0.0011	-0.0015	0.0003	0.0018
Multiple myeloma	B.1.27	-0.0007	-0.0001	-0.0008	-0.0007	0.0001
Leukemia	B.1.28	0.0141	-0.0007	0.0133	0.0208	0.0075
Other malignant neoplasms	B.1.29	0.0061	-0.0012	0.0049	0.0086	0.0037
Other neoplasms	B.1.30	-0.0002	-0.0003	-0.0005	-0.0001	0.0004
Cardiovascular diseases	B.2	0.8607	-0.0529	0.8078	0.9477	0.1399
Rheumatic heart disease	B.2.1	0.0425	0.0001	0.0426	0.0475	0.0049
Ischemic heart disease	B.2.2	0.2011	-0.0020	0.1991	0.2095	0.0103
Stroke	B.2.3	0.6161	-0.0439	0.5722	0.6783	0.1061
Hypertensive heart disease	B.2.4	-0.0097	-0.0022	-0.0119	-0.0055	0.0064
Non-rheumatic valvular heart disease	B.2.5	0.0013	-0.0004	0.0009	0.0013	0.0004
Cardiomyopathy and myocarditis	B.2.6	0.0059	-0.0001	0.0058	0.0101	0.0042
Atrial fibrillation and flutter	B.2.8	-0.0002	-0.0049	-0.0050	0.0007	0.0057
Aortic aneurysm	B.2.9	0.0004	0.0000	0.0004	0.0007	0.0003
Peripheral artery disease	B.2.10	-0.0001	0.0005	0.0004	0.0000	-0.0005
Endocarditis	B.2.11	0.0003	0.0000	0.0003	0.0007	0.0004
Other cardiovascular and circulatory diseases	B.2.12	0.0030	0.0000	0.0030	0.0045	0.0015
Chronic respiratory diseases	B.3	0.5909	0.0411	0.6321	0.5984	-0.0336
Chronic obstructive pulmonary disease	B.3.1	0.5702	0.0476	0.6178	0.5758	-0.0420
Pneumoconiosis	B.3.2	0.0040	0.0034	0.0074	0.0046	-0.0028
Asthma	B.3.3	0.0138	-0.0100	0.0037	0.0144	0.0107
Interstitial lung disease and pulmonary sarcoidosis	B.3.4	0.0009	-0.0004	0.0004	0.0012	0.0008
Other chronic respiratory diseases	B.3.5	0.0022	0.0006	0.0027	0.0024	-0.0004
Digestive diseases	B.4	0.0983	0.0055	0.1038	0.1241	0.0204
Cirrhosis and other chronic liver diseases	B.4.1	0.0528	0.0002	0.0530	0.0660	0.0129
Upper digestive system diseases	B.4.2	0.0274	-0.0008	0.0266	0.0310	0.0044
Appendicitis	B.4.3	0.0011	-0.0001	0.0010	0.0012	0.0002
Paralytic ileus and intestinal obstruction	B.4.4	0.0039	0.0001	0.0040	0.0103	0.0063
Inguinal, femoral, and abdominal hernia	B.4.5	0.0005	0.0000	0.0005	0.0012	0.0007
Inflammatory bowel disease	B.4.6	0.0023	-0.0006	0.0017	0.0027	0.0010
Vascular intestinal disorders	B.4.7	0.0006	0.0000	0.0006	0.0007	0.0001
Gallbladder and biliary diseases	B.4.8	0.0053	0.0068	0.0120	0.0056	-0.0064
Pancreatitis	B.4.9	0.0025	0.0000	0.0025	0.0029	0.0004
Other digestive diseases	B.4.10	0.0017	0.0001	0.0018	0.0025	0.0007
Neurological disorders	B.5	0.0287	-0.0175	0.0112	0.0315	0.0204
Alzheimer's disease and other dementias	B.5.1	0.0166	-0.0155	0.0012	0.0161	0.0150
Parkinson's disease	B.5.2	0.0089	0.0006	0.0095	0.0101	0.0006
Idiopathic epilepsy	B.5.3	0.0040	0.0057	0.0096	0.0054	-0.0042
Multiple sclerosis	B.5.4	0.0001	-0.0001	0.0000	0.0002	0.0001
Motor neuron disease	B.5.5	-0.0005	-0.0001	-0.0006	-0.0005	0.0001
Headache disorders	B.5.6	0.0000	-0.0073	-0.0073	0.0000	0.0073
Other neurological disorders	B.5.7	-0.0004	-0.0009	-0.0013	0.0002	0.0015

Causes	Level	ΔHALE			ΔLE	ΔLED
		Mortality Effect	Disability Effect	Total Effect	Total Effect	
Mental disorders	B.6	0.0000	-0.0049	-0.0049	0.0000	0.0049
Schizophrenia	B.6.1	0.0000	-0.0030	-0.0030	0.0000	0.0030
Depressive disorders	B.6.2	0.0000	-0.0092	-0.0092	0.0000	0.0092
Bipolar disorder	B.6.3	0.0000	0.0001	0.0001	0.0000	-0.0001
Anxiety disorders	B.6.4	0.0000	0.0085	0.0085	0.0000	-0.0085
Eating disorders	B.6.5	0.0000	-0.0032	-0.0032	0.0000	0.0032
Autism spectrum disorders	B.6.6	0.0000	-0.0003	-0.0003	0.0000	0.0003
Attention-deficit/hyperactivity disorder	B.6.7	0.0000	0.0002	0.0002	0.0000	-0.0002
Conduct disorder	B.6.8	0.0000	-0.0001	-0.0001	0.0000	0.0001
Idiopathic developmental intellectual disability	B.6.9	0.0000	0.0020	0.0020	0.0000	-0.0020
Other mental disorders	B.6.10	0.0000	0.0001	0.0001	0.0000	-0.0001
Substance use disorders	B.7	0.0035	-0.0229	-0.0194	0.0048	0.0241
Alcohol use disorders	B.7.1	0.0049	-0.0089	-0.0040	0.0060	0.0100
Drug use disorders	B.7.2	-0.0013	-0.0140	-0.0154	-0.0012	0.0142
Diabetes and kidney diseases	B.8	0.0334	0.0402	0.0736	0.0431	-0.0304
Diabetes mellitus	B.8.1	0.0067	0.0275	0.0342	0.0118	-0.0224
Chronic kidney disease	B.8.2	0.0235	0.0127	0.0362	0.0278	-0.0084
Acute glomerulonephritis	B.8.3	0.0031	0.0000	0.0031	0.0036	0.0004
Skin and subcutaneous diseases	B.9	0.0025	-0.0025	0.0000	0.0024	0.0024
Dermatitis	B.9.1	0.0000	-0.0006	-0.0006	0.0000	0.0006
Psoriasis	B.9.2	0.0000	0.0033	0.0033	0.0000	-0.0033
Bacterial skin diseases	B.9.3	0.0002	0.0000	0.0002	0.0004	0.0002
Scabies	B.9.4	0.0000	0.0005	0.0005	0.0000	-0.0005
Fungal skin diseases	B.9.5	0.0000	0.0001	0.0001	0.0000	-0.0001
Viral skin diseases	B.9.6	0.0000	0.0000	0.0000	0.0000	0.0000
Acne vulgaris	B.9.7	0.0000	-0.0040	-0.0040	0.0000	0.0040
Alopecia areata	B.9.8	0.0000	0.0000	0.0000	0.0000	0.0000
Pruritus	B.9.9	0.0000	-0.0003	-0.0003	0.0000	0.0003
Urticaria	B.9.10	0.0000	0.0000	0.0000	0.0000	0.0000
Decubitus ulcer	B.9.11	0.0022	0.0000	0.0022	0.0019	-0.0003
Other skin and subcutaneous diseases	B.9.12	0.0001	-0.0017	-0.0016	0.0001	0.0017
Sense organ diseases	B.10	0.0000	0.0459	0.0459	0.0000	-0.0459
Blindness and vision loss	B.10.1	0.0000	0.0297	0.0297	0.0000	-0.0297
Age-related and other hearing loss	B.10.2	0.0000	0.0169	0.0169	0.0000	-0.0169
Other sense organ diseases	B.10.3	0.0000	-0.0007	-0.0007	0.0000	0.0007
Musculoskeletal disorders	B.11	0.0020	-0.0001	0.0018	0.0024	0.0006
Rheumatoid arthritis	B.11.1	0.0010	0.0013	0.0023	0.0015	-0.0009
Osteoarthritis	B.11.2	0.0000	-0.0046	-0.0046	0.0000	0.0046
Low back pain	B.11.3	0.0000	0.0265	0.0265	0.0000	-0.0265
Neck pain	B.11.4	0.0000	-0.0001	-0.0001	0.0000	0.0001
Gout	B.11.5	0.0000	-0.0035	-0.0035	0.0000	0.0035
Other musculoskeletal disorders	B.11.6	0.0009	-0.0197	-0.0188	0.0009	0.0197

Causes	Level	ΔHALE			ΔLE	ΔLED
		Mortality Effect	Disability Effect	Total Effect	Total Effect	
Other non-communicable diseases	B.12	0.0170	-0.0006	0.0164	0.1236	0.1072
Congenital birth defects	B.12.1	0.0107	0.0014	0.0122	0.1115	0.0993
Urinary diseases and male infertility	B.12.2	0.0045	0.0005	0.0050	0.0050	0.0000
Gynecological diseases	B.12.3	0.0002	-0.0024	-0.0022	0.0002	0.0024
Hemoglobinopathies and hemolytic anemias	B.12.4	0.0038	0.0083	0.0122	0.0060	-0.0062
Endocrine, metabolic, blood, and immune disorders	B.12.5	-0.0023	-0.0009	-0.0032	-0.0009	0.0023
Oral disorders	B.12.6	0.0000	-0.0075	-0.0075	0.0000	0.0075
**Injuries**	C	0.4055	-0.1394	0.2661	0.5019	0.2358
Transport injuries	C.1	0.1922	-0.0352	0.1571	0.2104	0.0534
Road injuries	C.1.1	0.1823	-0.0346	0.1478	0.1997	0.0520
Other transport injuries	C.1.2	0.0099	-0.0006	0.0093	0.0107	0.0014
Unintentional injuries	C.2	0.1363	-0.1060	0.0303	0.2063	0.1760
Falls	C.2.1	0.0149	-0.0895	-0.0746	0.0204	0.0950
Drowning	C.2.2	0.0428	-0.0002	0.0426	0.0750	0.0324
Fire, heat, and hot substances	C.2.3	0.0044	-0.0036	0.0008	0.0060	0.0052
Poisonings	C.2.4	0.0131	-0.0005	0.0127	0.0168	0.0042
Exposure to mechanical forces	C.2.5	0.0226	-0.0063	0.0163	0.0261	0.0098
Adverse effects of medical treatment	C.2.6	0.0020	-0.0001	0.0019	0.0034	0.0015
Animal contact	C.2.7	0.0013	-0.0009	0.0005	0.0018	0.0014
Foreign body	C.2.8	0.0014	-0.0055	-0.0041	0.0201	0.0242
Environmental heat and cold exposure	C.2.9	0.0010	-0.0006	0.0004	0.0014	0.0010
Exposure to forces of nature	C.2.10	0.0136	0.0010	0.0146	0.0147	0.0001
Other unintentional injuries	C.2.11	0.0191	0.0001	0.0192	0.0205	0.0013
Self-harm and interpersonal violence	C.3	0.0770	0.0017	0.0787	0.0852	0.0065
Self-harm	C.3.1	0.0619	0.0010	0.0629	0.0691	0.0062
Interpersonal violence	C.3.2	0.0152	0.0007	0.0159	0.0161	0.0002
Conflict and terrorism	C.3.3	0.0000	0.0000	0.0000	0.0000	0.0000
Executions and police conflict	C.3.4	-0.0001	0.0000	-0.0001	-0.0001	0.0000

Table S6a | Cause-specific effects of mortality and disability (years) on changes in life expectancy (LE) and health-adjusted life expectancy (HALE) for males from 1990 to 2019.

Causes	Level	ΔHALE			ΔLE	ΔLED
		Mortality Effect	Disability Effect	Total Effect	Total Effect	
**Communicable, maternal, neonatal, and nutritional diseases**	A	1.0082	0.1694	1.1775	3.2198	2.0423
HIV/AIDS and sexually transmitted infections	A.1	-0.0738	-0.0044	-0.0782	-0.0443	0.0339
HIV/AIDS	A.1.1	-0.0754	-0.0047	-0.0801	-0.0503	0.0298
Sexually transmitted infections excluding HIV	A.1.2	0.0016	0.0002	0.0019	0.0060	0.0041
Respiratory infections and tuberculosis	A.2	0.7789	0.0492	0.8281	1.7113	0.8832
Tuberculosis	A.2.1	0.4321	0.0387	0.4708	0.3796	-0.0912
Lower respiratory infections	A.2.2	0.3185	0.0026	0.3211	1.3020	0.9809
Upper respiratory infections	A.2.3	0.0280	0.0001	0.0282	0.0293	0.0012
Otitis media	A.2.4	0.0003	0.0077	0.0080	0.0003	-0.0077
Enteric infections	A.3	0.0770	0.0000	0.0770	0.2701	0.1931
Diarrheal diseases	A.3.1	0.0689	-0.0001	0.0688	0.2644	0.1955
Typhoid and paratyphoid	A.3.2	0.0064	0.0001	0.0065	0.0038	-0.0027
Invasive Non-typhoidal Salmonella (iNTS)	A.3.3	0.0013	0.0000	0.0013	0.0011	-0.0003
Other intestinal infectious diseases	A.3.4	0.0004	0.0000	0.0004	0.0008	0.0004
Neglected tropical diseases and malaria	A.4	0.0146	0.0951	0.1097	0.0134	-0.0963
Malaria	A.4.1	0.0052	0.0005	0.0057	0.0036	-0.0021
Chagas disease	A.4.2	0.0000	0.0000	0.0000	0.0000	0.0000
Leishmaniasis	A.4.3	0.0000	0.0000	0.0000	0.0000	0.0000
African trypanosomiasis	A.4.4	0.0000	0.0000	0.0000	0.0000	0.0000
Schistosomiasis	A.4.5	0.0046	0.0030	0.0076	0.0037	-0.0039
Cysticercosis	A.4.6	0.0002	0.0047	0.0049	0.0001	-0.0047
Cystic echinococcosis	A.4.7	0.0001	0.0000	0.0001	0.0001	0.0000
Lymphatic filariasis	A.4.8	0.0000	0.0000	0.0000	0.0000	0.0000
Onchocerciasis	A.4.9	0.0000	0.0000	0.0000	0.0000	0.0000
Trachoma	A.4.10	0.0000	0.0027	0.0027	0.0000	-0.0027
Dengue	A.4.11	0.0001	-0.0022	-0.0020	0.0002	0.0023
Yellow fever	A.4.12	0.0000	0.0000	0.0000	0.0000	0.0000
Rabies	A.4.13	0.0024	0.0000	0.0024	0.0026	0.0002
Intestinal nematode infections	A.4.14	0.0007	0.0364	0.0371	0.0013	-0.0358
Food-borne trematodiases	A.4.15	0.0000	0.0437	0.0437	0.0000	-0.0437
Leprosy	A.4.16	0.0000	0.0001	0.0001	0.0000	-0.0001
Ebola	A.4.17	0.0000	0.0000	0.0000	0.0000	0.0000
Zika virus	A.4.18	0.0000	0.0000	0.0000	0.0000	0.0000
Guinea worm disease	A.4.19	0.0000	0.0000	0.0000	0.0000	0.0000
Other neglected tropical diseases	A.4.20	0.0013	0.0063	0.0076	0.0019	-0.0057
Other infectious diseases	A.5	0.1824	0.0162	0.1987	0.4437	0.2450
Meningitis	A.5.1	0.0410	0.0049	0.0459	0.1039	0.0580
Encephalitis	A.5.2	0.0067	0.0031	0.0098	0.0183	0.0085
Diphtheria	A.5.3	0.0003	0.0000	0.0003	0.0006	0.0003

Causes	Level	ΔHALE			ΔLE	ΔLED
		Mortality Effect	Disability Effect	Total Effect	Total Effect	
Whooping cough	A.5.4	0.0137	0.0009	0.0145	0.0779	0.0634
Tetanus	A.5.5	0.0172	0.0001	0.0173	0.0671	0.0499
Measles	A.5.6	0.0240	0.0007	0.0246	0.0826	0.0579
Varicella and herpes zoster	A.5.7	0.0050	0.0000	0.0050	0.0055	0.0004
Acute hepatitis	A.5.8	0.0634	0.0015	0.0649	0.0667	0.0019
Other unspecified infectious diseases	A.5.9	0.0112	0.0052	0.0164	0.0211	0.0047
Maternal and neonatal disorders	A.6	0.0086	-0.0899	-0.0812	0.7395	0.8207
Maternal disorders	A.6.1	0.0000	0.0000	0.0000	0.0000	0.0000
Neonatal disorders	A.6.2	0.0086	-0.0899	-0.0812	0.7395	0.8207
Nutritional deficiencies	A.7	0.0204	0.1031	0.1235	0.0861	-0.0374
Protein-energy malnutrition	A.7.1	0.0219	-0.0212	0.0007	0.0839	0.0832
Iodine deficiency	A.7.2	0.0000	0.0051	0.0051	0.0000	-0.0051
Vitamin A deficiency	A.7.3	0.0000	0.0044	0.0044	0.0000	-0.0044
Dietary iron deficiency	A.7.4	0.0000	0.1194	0.1194	0.0000	-0.1194
Other nutritional deficiencies	A.7.5	-0.0015	-0.0046	-0.0062	0.0022	0.0084
**Non-communicable diseases**	B	5.1347	0.1102	5.2449	4.5046	-0.7403
Neoplasms	B.1	0.6692	-0.0487	0.6205	0.7585	0.1380
Lip and oral cavity cancer	B.1.1	-0.0112	-0.0008	-0.0120	-0.0086	0.0035
Nasopharynx cancer	B.1.2	0.0368	-0.0028	0.0340	0.0298	-0.0043
Other pharynx cancer	B.1.3	0.0022	-0.0001	0.0021	0.0015	-0.0005
Esophageal cancer	B.1.4	0.1510	0.0014	0.1525	0.1454	-0.0070
Stomach cancer	B.1.5	0.3101	-0.0015	0.3086	0.2848	-0.0238
Colon and rectum cancer	B.1.6	-0.1211	-0.0154	-0.1365	-0.0802	0.0563
Liver cancer	B.1.7	0.4475	0.0039	0.4515	0.3886	-0.0628
Gallbladder and biliary tract cancer	B.1.8	-0.0068	-0.0002	-0.0070	-0.0046	0.0024
Pancreatic cancer	B.1.9	-0.0581	-0.0007	-0.0589	-0.0428	0.0161
Larynx cancer	B.1.10	0.0095	-0.0009	0.0086	0.0086	0.0001
Tracheal, bronchus, and lung cancer	B.1.11	-0.1445	-0.0054	-0.1499	-0.0573	0.0926
Malignant skin melanoma	B.1.12	0.0013	-0.0003	0.0011	0.0011	0.0000
Non-melanoma skin cancer	B.1.13	-0.0022	-0.0002	-0.0024	-0.0009	0.0015
Breast cancer	B.1.14	-0.0032	-0.0004	-0.0037	-0.0028	0.0008
Cervical cancer	B.1.15	0.0000	0.0000	0.0000	0.0000	0.0000
Uterine cancer	B.1.16	0.0000	0.0000	0.0000	0.0000	0.0000
Ovarian cancer	B.1.17	0.0000	0.0000	0.0000	0.0000	0.0000
Prostate cancer	B.1.18	0.0080	-0.0078	0.0001	0.0084	0.0083
Testicular cancer	B.1.19	0.0004	-0.0007	-0.0003	0.0004	0.0007
Kidney cancer	B.1.20	-0.0197	-0.0014	-0.0211	-0.0132	0.0079
Bladder cancer	B.1.21	0.0033	-0.0029	0.0004	0.0032	0.0027
Brain and central nervous system cancer	B.1.22	0.0080	-0.0005	0.0075	0.0156	0.0082
Thyroid cancer	B.1.23	-0.0026	-0.0005	-0.0031	-0.0014	0.0018
Mesothelioma	B.1.24	-0.0011	0.0000	-0.0011	-0.0009	0.0002
Hodgkin lymphoma	B.1.25	0.0113	-0.0002	0.0111	0.0085	-0.0026

Causes	Level	ΔHALE			ΔLE	ΔLED
		Mortality Effect	Disability Effect	Total Effect	Total Effect	
Non-Hodgkin lymphoma	B.1.26	-0.0193	-0.0037	-0.0230	-0.0130	0.0101
Multiple myeloma	B.1.27	-0.0036	-0.0004	-0.0040	-0.0021	0.0019
Leukemia	B.1.28	0.0628	-0.0007	0.0621	0.0724	0.0103
Other malignant neoplasms	B.1.29	0.0120	-0.0047	0.0072	0.0186	0.0114
Other neoplasms	B.1.30	-0.0017	-0.0016	-0.0033	-0.0009	0.0024
Cardiovascular diseases	B.2	1.2564	-0.0493	1.2071	1.1889	-0.0182
Rheumatic heart disease	B.2.1	0.1820	0.0016	0.1836	0.1262	-0.0574
Ischemic heart disease	B.2.2	-0.3899	0.0002	-0.3897	-0.0817	0.3080
Stroke	B.2.3	1.1494	-0.0525	1.0968	0.9115	-0.1853
Hypertensive heart disease	B.2.4	0.2986	0.0055	0.3041	0.2000	-0.1041
Non-rheumatic valvular heart disease	B.2.5	0.0036	-0.0007	0.0028	0.0023	-0.0006
Cardiomyopathy and myocarditis	B.2.6	-0.0013	0.0000	-0.0013	0.0085	0.0098
Atrial fibrillation and flutter	B.2.8	-0.0047	-0.0021	-0.0068	-0.0007	0.0061
Aortic aneurysm	B.2.9	-0.0005	0.0000	-0.0005	0.0004	0.0009
Peripheral artery disease	B.2.10	-0.0008	0.0008	0.0000	-0.0004	-0.0004
Endocarditis	B.2.11	0.0046	0.0000	0.0046	0.0063	0.0017
Other cardiovascular and circulatory diseases	B.2.12	0.0154	-0.0021	0.0134	0.0164	0.0030
Chronic respiratory diseases	B.3	2.4486	0.0845	2.5331	1.4886	-1.0446
Chronic obstructive pulmonary disease	B.3.1	2.3353	0.0711	2.4064	1.4116	-0.9948
Pneumoconiosis	B.3.2	0.0315	0.0033	0.0348	0.0235	-0.0114
Asthma	B.3.3	0.0827	0.0121	0.0948	0.0524	-0.0424
Interstitial lung disease and pulmonary sarcoidosis	B.3.4	-0.0003	-0.0009	-0.0012	0.0004	0.0017
Other chronic respiratory diseases	B.3.5	-0.0006	-0.0011	-0.0018	0.0006	0.0024
Digestive diseases	B.4	0.5565	0.0434	0.5998	0.4702	-0.1296
Cirrhosis and other chronic liver diseases	B.4.1	0.2972	0.0033	0.3005	0.2428	-0.0577
Upper digestive system diseases	B.4.2	0.1493	0.0199	0.1692	0.1130	-0.0561
Appendicitis	B.4.3	0.0091	-0.0003	0.0088	0.0065	-0.0023
Paralytic ileus and intestinal obstruction	B.4.4	0.0197	-0.0003	0.0195	0.0379	0.0184
Inguinal, femoral, and abdominal hernia	B.4.5	0.0042	0.0024	0.0066	0.0085	0.0020
Inflammatory bowel disease	B.4.6	0.0086	-0.0031	0.0055	0.0060	0.0005
Vascular intestinal disorders	B.4.7	0.0007	0.0000	0.0007	0.0007	0.0000
Gallbladder and biliary diseases	B.4.8	0.0227	0.0202	0.0429	0.0164	-0.0266
Pancreatitis	B.4.9	0.0106	0.0002	0.0108	0.0076	-0.0032
Other digestive diseases	B.4.10	0.0343	0.0011	0.0354	0.0308	-0.0047
Neurological disorders	B.5	0.0299	-0.0596	-0.0297	0.0389	0.0686
Alzheimer's disease and other dementias	B.5.1	-0.0098	-0.0265	-0.0363	-0.0018	0.0345
Parkinson's disease	B.5.2	0.0097	-0.0085	0.0012	0.0086	0.0073
Idiopathic epilepsy	B.5.3	0.0304	-0.0031	0.0273	0.0276	0.0003
Multiple sclerosis	B.5.4	0.0007	-0.0001	0.0006	0.0006	-0.0001
Motor neuron disease	B.5.5	0.0009	-0.0001	0.0008	0.0027	0.0019
Headache disorders	B.5.6	0.0000	-0.0172	-0.0172	0.0000	0.0172
Other neurological disorders	B.5.7	-0.0020	-0.0042	-0.0062	0.0013	0.0074

Causes	Level	ΔHALE			ΔLE	ΔLED
		Mortality Effect	Disability Effect	Total Effect	Total Effect	
Mental disorders	B.6	-0.0001	0.0155	0.0154	-0.0001	-0.0154
Schizophrenia	B.6.1	0.0000	-0.0055	-0.0055	0.0000	0.0055
Depressive disorders	B.6.2	0.0000	0.0080	0.0080	0.0000	-0.0080
Bipolar disorder	B.6.3	0.0000	-0.0001	-0.0001	0.0000	0.0001
Anxiety disorders	B.6.4	0.0000	0.0157	0.0157	0.0000	-0.0157
Eating disorders	B.6.5	-0.0001	-0.0059	-0.0060	-0.0001	0.0060
Autism spectrum disorders	B.6.6	0.0000	-0.0022	-0.0022	0.0000	0.0022
Attention-deficit/hyperactivity disorder	B.6.7	0.0000	-0.0011	-0.0011	0.0000	0.0011
Conduct disorder	B.6.8	0.0000	-0.0012	-0.0012	0.0000	0.0012
Idiopathic developmental intellectual disability	B.6.9	0.0000	0.0080	0.0080	0.0000	-0.0080
Other mental disorders	B.6.10	0.0000	-0.0002	-0.0002	0.0000	0.0002
Substance use disorders	B.7	0.0443	0.0065	0.0508	0.0266	-0.0242
Alcohol use disorders	B.7.1	-0.0167	0.0003	-0.0164	-0.0116	0.0049
Drug use disorders	B.7.2	0.0610	0.0062	0.0672	0.0382	-0.0290
Diabetes and kidney diseases	B.8	0.0454	-0.0766	-0.0311	0.0540	0.0851
Diabetes mellitus	B.8.1	-0.0255	-0.0713	-0.0968	-0.0062	0.0906
Chronic kidney disease	B.8.2	0.0484	-0.0053	0.0431	0.0434	0.0003
Acute glomerulonephritis	B.8.3	0.0225	0.0000	0.0226	0.0168	-0.0058
Skin and subcutaneous diseases	B.9	0.0116	-0.0046	0.0070	0.0093	0.0023
Dermatitis	B.9.1	0.0000	-0.0003	-0.0003	0.0000	0.0003
Psoriasis	B.9.2	0.0000	0.0106	0.0106	0.0000	-0.0106
Bacterial skin diseases	B.9.3	0.0129	0.0001	0.0130	0.0099	-0.0031
Scabies	B.9.4	0.0000	0.0018	0.0018	0.0000	-0.0018
Fungal skin diseases	B.9.5	0.0000	0.0007	0.0007	0.0000	-0.0007
Viral skin diseases	B.9.6	0.0000	-0.0001	-0.0001	0.0000	0.0001
Acne vulgaris	B.9.7	0.0000	-0.0118	-0.0118	0.0000	0.0118
Alopecia areata	B.9.8	0.0000	0.0000	0.0000	0.0000	0.0000
Pruritus	B.9.9	0.0000	-0.0008	-0.0008	0.0000	0.0008
Urticaria	B.9.10	0.0000	-0.0001	-0.0001	0.0000	0.0001
Decubitus ulcer	B.9.11	-0.0016	-0.0002	-0.0018	-0.0007	0.0011
Other skin and subcutaneous diseases	B.9.12	0.0003	-0.0045	-0.0042	0.0001	0.0043
Sense organ diseases	B.10	0.0000	0.0453	0.0453	0.0000	-0.0453
Blindness and vision loss	B.10.1	0.0000	0.0253	0.0253	0.0000	-0.0253
Age-related and other hearing loss	B.10.2	0.0000	0.0214	0.0214	0.0000	-0.0214
Other sense organ diseases	B.10.3	0.0000	-0.0014	-0.0014	0.0000	0.0014
Musculoskeletal disorders	B.11	0.0007	0.0794	0.0801	0.0014	-0.0787
Rheumatoid arthritis	B.11.1	-0.0009	-0.0009	-0.0018	0.0001	0.0019
Osteoarthritis	B.11.2	0.0000	-0.0168	-0.0168	0.0000	0.0168
Low back pain	B.11.3	0.0000	0.1996	0.1996	0.0000	-0.1996
Neck pain	B.11.4	0.0000	-0.0047	-0.0047	0.0000	0.0047
Gout	B.11.5	0.0000	-0.0089	-0.0089	0.0000	0.0089
Other musculoskeletal disorders	B.11.6	0.0017	-0.0890	-0.0873	0.0014	0.0887

Causes	Level	ΔHALE			ΔLE	ΔLED
		Mortality Effect	Disability Effect	Total Effect	Total Effect	
Other non-communicable diseases	B.12	0.0721	0.0746	0.1467	0.4682	0.3215
Congenital birth defects	B.12.1	0.0466	0.0090	0.0555	0.4260	0.3705
Urinary diseases and male infertility	B.12.2	0.0139	0.0119	0.0259	0.0106	-0.0153
Gynecological diseases	B.12.3	0.0000	0.0000	0.0000	0.0000	0.0000
Hemoglobinopathies and hemolytic anemias	B.12.4	0.0133	0.0388	0.0521	0.0188	-0.0333
Endocrine, metabolic, blood, and immune disorders	B.12.5	-0.0017	0.0071	0.0054	0.0059	0.0005
Oral disorders	B.12.6	0.0000	0.0078	0.0078	0.0000	-0.0078
**Injuries**	C	1.2773	-0.0501	1.2273	1.1974	-0.0299
Transport injuries	C.1	0.2812	-0.0653	0.2158	0.2398	0.0240
Road injuries	C.1.1	0.2350	-0.0704	0.1647	0.2061	0.0415
Other transport injuries	C.1.2	0.0461	0.0051	0.0512	0.0337	-0.0175
Unintentional injuries	C.2	0.4645	0.0062	0.4707	0.6221	0.1514
Falls	C.2.1	-0.0044	-0.0018	-0.0062	0.0253	0.0315
Drowning	C.2.2	0.3015	0.0022	0.3038	0.3576	0.0538
Fire, heat, and hot substances	C.2.3	0.0305	0.0006	0.0311	0.0348	0.0037
Poisonings	C.2.4	0.0104	-0.0001	0.0102	0.0233	0.0130
Exposure to mechanical forces	C.2.5	0.0474	-0.0123	0.0351	0.0390	0.0039
Adverse effects of medical treatment	C.2.6	0.0169	0.0000	0.0169	0.0235	0.0065
Animal contact	C.2.7	0.0246	0.0031	0.0277	0.0230	-0.0047
Foreign body	C.2.8	0.0014	0.0115	0.0129	0.0608	0.0479
Environmental heat and cold exposure	C.2.9	0.0317	0.0026	0.0342	0.0279	-0.0063
Exposure to forces of nature	C.2.10	0.0046	-0.0018	0.0028	0.0030	0.0002
Other unintentional injuries	C.2.11	-0.0001	0.0021	0.0020	0.0038	0.0018
Self-harm and interpersonal violence	C.3	0.5316	0.0091	0.5407	0.3355	-0.2053
Self-harm	C.3.1	0.3697	0.0022	0.3719	0.2309	-0.1409
Interpersonal violence	C.3.2	0.1376	0.0069	0.1445	0.0920	-0.0525
Conflict and terrorism	C.3.3	0.0001	0.0000	0.0002	0.0001	-0.0001
Executions and police conflict	C.3.4	0.0242	0.0000	0.0242	0.0125	-0.0117

Table S6b | Cause-specific effects of mortality and disability (years) on changes in life expectancy (LE) and health-adjusted life expectancy (HALE) for females from 1990 to 2019.

Causes	Level	ΔHALE			ΔLE	ΔLED
		Mortality Effect	Disability Effect	Total Effect	Total Effect	
**Communicable, maternal, neonatal, and nutritional diseases**	A	1.2126	0.2983	1.5109	3.7428	2.2319
HIV/AIDS and sexually transmitted infections	A.1	-0.0218	-0.0011	-0.0228	-0.0147	0.0081
HIV/AIDS	A.1.1	-0.0245	-0.0018	-0.0264	-0.0226	0.0038
Sexually transmitted infections excluding HIV	A.1.2	0.0028	0.0008	0.0035	0.0078	0.0043
Respiratory infections and tuberculosis	A.2	0.8117	0.0369	0.8486	2.0374	1.1888
Tuberculosis	A.2.1	0.2945	0.0269	0.3215	0.3572	0.0357
Lower respiratory infections	A.2.2	0.4748	0.0042	0.4790	1.6323	1.1533
Upper respiratory infections	A.2.3	0.0418	-0.0011	0.0407	0.0473	0.0066
Otitis media	A.2.4	0.0005	0.0069	0.0074	0.0006	-0.0068
Enteric infections	A.3	0.0691	0.0012	0.0703	0.3067	0.2364
Diarrheal diseases	A.3.1	0.0616	0.0011	0.0627	0.3006	0.2379
Typhoid and paratyphoid	A.3.2	0.0055	0.0001	0.0056	0.0032	-0.0024
Invasive Non-typhoidal Salmonella (iNTS)	A.3.3	0.0014	0.0000	0.0014	0.0015	0.0000
Other intestinal infectious diseases	A.3.4	0.0005	0.0000	0.0005	0.0014	0.0009
Neglected tropical diseases and malaria	A.4	0.0129	0.1040	0.1169	0.0154	-0.1014
Malaria	A.4.1	0.0041	0.0009	0.0051	0.0042	-0.0008
Chagas disease	A.4.2	0.0000	0.0000	0.0000	0.0000	0.0000
Leishmaniasis	A.4.3	0.0000	0.0000	0.0000	0.0000	0.0000
African trypanosomiasis	A.4.4	0.0000	0.0000	0.0000	0.0000	0.0000
Schistosomiasis	A.4.5	0.0049	0.0058	0.0107	0.0053	-0.0054
Cysticercosis	A.4.6	0.0002	0.0074	0.0076	0.0002	-0.0074
Cystic echinococcosis	A.4.7	0.0001	0.0000	0.0001	0.0001	0.0000
Lymphatic filariasis	A.4.8	0.0000	0.0000	0.0000	0.0000	0.0000
Onchocerciasis	A.4.9	0.0000	0.0000	0.0000	0.0000	0.0000
Trachoma	A.4.10	0.0000	0.0039	0.0039	0.0000	-0.0039
Dengue	A.4.11	0.0000	-0.0023	-0.0022	0.0001	0.0023
Yellow fever	A.4.12	0.0000	0.0000	0.0000	0.0000	0.0000
Rabies	A.4.13	0.0017	0.0000	0.0017	0.0021	0.0003
Intestinal nematode infections	A.4.14	0.0007	0.0459	0.0466	0.0014	-0.0451
Food-borne trematodiases	A.4.15	0.0000	0.0316	0.0316	0.0000	-0.0316
Leprosy	A.4.16	0.0000	0.0000	0.0000	0.0000	0.0000
Ebola	A.4.17	0.0000	0.0000	0.0000	0.0000	0.0000
Zika virus	A.4.18	0.0000	0.0000	0.0000	0.0000	0.0000
Guinea worm disease	A.4.19	0.0000	0.0000	0.0000	0.0000	0.0000
Other neglected tropical diseases	A.4.20	0.0010	0.0109	0.0118	0.0019	-0.0099
Other infectious diseases	A.5	0.1594	0.0206	0.1800	0.4560	0.2760
Meningitis	A.5.1	0.0387	0.0051	0.0438	0.0932	0.0494
Encephalitis	A.5.2	0.0070	0.0031	0.0101	0.0213	0.0111
Diphtheria	A.5.3	0.0003	0.0000	0.0003	0.0006	0.0004

Causes	Level	ΔHALE			ΔLE	ΔLED
		Mortality Effect	Disability Effect	Total Effect	Total Effect	
Whooping cough	A.5.4	0.0209	0.0011	0.0220	0.1159	0.0939
Tetanus	A.5.5	0.0093	0.0000	0.0093	0.0461	0.0367
Measles	A.5.6	0.0332	0.0007	0.0339	0.1019	0.0679
Varicella and herpes zoster	A.5.7	0.0084	0.0000	0.0084	0.0085	0.0001
Acute hepatitis	A.5.8	0.0358	0.0011	0.0369	0.0545	0.0176
Other unspecified infectious diseases	A.5.9	0.0058	0.0095	0.0153	0.0141	-0.0012
Maternal and neonatal disorders	A.6	0.1296	-0.0937	0.0359	0.7934	0.7575
Maternal disorders	A.6.1	0.1217	0.0081	0.1298	0.0802	-0.0496
Neonatal disorders	A.6.2	0.0079	-0.1018	-0.0940	0.7132	0.8072
Nutritional deficiencies	A.7	0.0517	0.2304	0.2821	0.1486	-0.1335
Protein-energy malnutrition	A.7.1	0.0523	-0.0049	0.0475	0.1452	0.0977
Iodine deficiency	A.7.2	0.0000	0.0003	0.0003	0.0000	-0.0003
Vitamin A deficiency	A.7.3	0.0000	0.0025	0.0025	0.0000	-0.0025
Dietary iron deficiency	A.7.4	0.0000	0.2319	0.2319	0.0000	-0.2319
Other nutritional deficiencies	A.7.5	-0.0006	0.0005	-0.0001	0.0034	0.0035
**Non-communicable diseases**	B	7.2870	0.3589	7.6459	7.6789	0.0330
Neoplasms	B.1	0.7711	-0.0334	0.7377	0.9586	0.2209
Lip and oral cavity cancer	B.1.1	0.0045	-0.0001	0.0044	0.0047	0.0003
Nasopharynx cancer	B.1.2	0.0312	-0.0006	0.0306	0.0333	0.0027
Other pharynx cancer	B.1.3	0.0015	0.0000	0.0015	0.0019	0.0004
Esophageal cancer	B.1.4	0.1619	0.0020	0.1638	0.1986	0.0348
Stomach cancer	B.1.5	0.2823	0.0021	0.2844	0.3283	0.0439
Colon and rectum cancer	B.1.6	-0.0075	-0.0079	-0.0155	-0.0013	0.0142
Liver cancer	B.1.7	0.2253	0.0027	0.2280	0.2629	0.0349
Gallbladder and biliary tract cancer	B.1.8	0.0007	-0.0001	0.0006	0.0015	0.0010
Pancreatic cancer	B.1.9	-0.0363	-0.0006	-0.0369	-0.0378	-0.0009
Larynx cancer	B.1.10	0.0057	0.0000	0.0057	0.0069	0.0011
Tracheal, bronchus, and lung cancer	B.1.11	-0.0812	-0.0031	-0.0843	-0.0644	0.0200
Malignant skin melanoma	B.1.12	0.0010	-0.0003	0.0007	0.0011	0.0005
Non-melanoma skin cancer	B.1.13	-0.0038	-0.0001	-0.0039	-0.0027	0.0013
Breast cancer	B.1.14	0.0109	-0.0145	-0.0036	0.0141	0.0177
Cervical cancer	B.1.15	0.0161	-0.0016	0.0145	0.0179	0.0034
Uterine cancer	B.1.16	0.0256	-0.0011	0.0245	0.0305	0.0060
Ovarian cancer	B.1.17	-0.0178	-0.0013	-0.0191	-0.0233	-0.0041
Prostate cancer	B.1.18	0.0000	0.0000	0.0000	0.0000	0.0000
Testicular cancer	B.1.19	0.0000	0.0000	0.0000	0.0000	0.0000
Kidney cancer	B.1.20	-0.0034	-0.0005	-0.0039	-0.0022	0.0017
Bladder cancer	B.1.21	0.0122	-0.0003	0.0119	0.0125	0.0006
Brain and central nervous system cancer	B.1.22	0.0134	-0.0011	0.0123	0.0180	0.0057
Thyroid cancer	B.1.23	0.0048	-0.0005	0.0043	0.0048	0.0006
Mesothelioma	B.1.24	0.0008	0.0000	0.0008	0.0008	0.0001
Hodgkin lymphoma	B.1.25	0.0080	-0.0001	0.0079	0.0080	0.0001

Causes	Level	ΔHALE			ΔLE	ΔLED
		Mortality Effect	Disability Effect	Total Effect	Total Effect	
Non-Hodgkin lymphoma	B.1.26	0.0018	-0.0020	-0.0003	0.0017	0.0020
Multiple myeloma	B.1.27	0.0014	-0.0002	0.0012	0.0021	0.0009
Leukemia	B.1.28	0.0770	-0.0001	0.0769	0.0966	0.0197
Other malignant neoplasms	B.1.29	0.0355	-0.0029	0.0326	0.0440	0.0114
Other neoplasms	B.1.30	-0.0003	-0.0010	-0.0013	-0.0001	0.0012
Cardiovascular diseases	B.2	2.5295	-0.1188	2.4108	2.7732	0.3625
Rheumatic heart disease	B.2.1	0.3859	0.0009	0.3868	0.3922	0.0055
Ischemic heart disease	B.2.2	-0.2253	-0.0089	-0.2342	0.0083	0.2425
Stroke	B.2.3	1.8593	-0.1042	1.7551	1.8880	0.1329
Hypertensive heart disease	B.2.4	0.4619	0.0026	0.4644	0.4205	-0.0439
Non-rheumatic valvular heart disease	B.2.5	0.0067	-0.0015	0.0052	0.0056	0.0004
Cardiomyopathy and myocarditis	B.2.6	0.0046	0.0000	0.0045	0.0146	0.0101
Atrial fibrillation and flutter	B.2.8	0.0110	-0.0066	0.0044	0.0104	0.0060
Aortic aneurysm	B.2.9	0.0028	0.0000	0.0028	0.0026	-0.0002
Peripheral artery disease	B.2.10	0.0000	0.0019	0.0019	0.0001	-0.0018
Endocarditis	B.2.11	0.0038	0.0000	0.0038	0.0068	0.0030
Other cardiovascular and circulatory diseases	B.2.12	0.0189	-0.0029	0.0160	0.0240	0.0080
Chronic respiratory diseases	B.3	3.1692	0.1375	3.3067	2.6772	-0.6295
Chronic obstructive pulmonary disease	B.3.1	3.0623	0.1237	3.1860	2.5817	-0.6044
Pneumoconiosis	B.3.2	0.0021	0.0011	0.0032	0.0021	-0.0011
Asthma	B.3.3	0.1030	0.0163	0.1193	0.0904	-0.0288
Interstitial lung disease and pulmonary sarcoidosis	B.3.4	0.0011	-0.0008	0.0003	0.0015	0.0012
Other chronic respiratory diseases	B.3.5	0.0007	-0.0028	-0.0021	0.0015	0.0037
Digestive diseases	B.4	0.4481	0.0536	0.5017	0.4979	-0.0038
Cirrhosis and other chronic liver diseases	B.4.1	0.1892	0.0026	0.1918	0.2142	0.0224
Upper digestive system diseases	B.4.2	0.1050	0.0204	0.1255	0.1034	-0.0220
Appendicitis	B.4.3	0.0108	-0.0002	0.0107	0.0095	-0.0012
Paralytic ileus and intestinal obstruction	B.4.4	0.0225	-0.0002	0.0223	0.0481	0.0257
Inguinal, femoral, and abdominal hernia	B.4.5	0.0017	0.0010	0.0027	0.0031	0.0004
Inflammatory bowel disease	B.4.6	0.0127	-0.0033	0.0094	0.0132	0.0038
Vascular intestinal disorders	B.4.7	0.0026	0.0000	0.0026	0.0026	0.0000
Gallbladder and biliary diseases	B.4.8	0.0489	0.0315	0.0803	0.0475	-0.0328
Pancreatitis	B.4.9	0.0131	0.0003	0.0134	0.0132	-0.0002
Other digestive diseases	B.4.10	0.0416	0.0013	0.0429	0.0430	0.0001
Neurological disorders	B.5	0.0703	-0.1023	-0.0320	0.0718	0.1038
Alzheimer's disease and other dementias	B.5.1	0.0139	-0.0599	-0.0460	0.0119	0.0579
Parkinson's disease	B.5.2	0.0268	-0.0056	0.0211	0.0258	0.0047
Idiopathic epilepsy	B.5.3	0.0271	-0.0029	0.0242	0.0274	0.0033
Multiple sclerosis	B.5.4	0.0012	-0.0002	0.0010	0.0013	0.0002
Motor neuron disease	B.5.5	0.0011	-0.0001	0.0010	0.0037	0.0027
Headache disorders	B.5.6	0.0000	-0.0294	-0.0294	0.0000	0.0294
Other neurological disorders	B.5.7	0.0003	-0.0043	-0.0040	0.0017	0.0057

Causes	Level	ΔHALE			ΔLE	ΔLED
		Mortality Effect	Disability Effect	Total Effect	Total Effect	
Mental disorders	B.6	-0.0001	0.0581	0.0580	-0.0001	-0.0581
Schizophrenia	B.6.1	0.0000	-0.0063	-0.0063	0.0000	0.0063
Depressive disorders	B.6.2	0.0000	0.0317	0.0317	0.0000	-0.0317
Bipolar disorder	B.6.3	0.0000	0.0000	0.0000	0.0000	0.0000
Anxiety disorders	B.6.4	0.0000	0.0345	0.0345	0.0000	-0.0345
Eating disorders	B.6.5	-0.0001	-0.0070	-0.0071	-0.0001	0.0071
Autism spectrum disorders	B.6.6	0.0000	-0.0005	-0.0005	0.0000	0.0005
Attention-deficit/hyperactivity disorder	B.6.7	0.0000	-0.0006	-0.0006	0.0000	0.0006
Conduct disorder	B.6.8	0.0000	-0.0006	-0.0006	0.0000	0.0006
Idiopathic developmental intellectual disability	B.6.9	0.0000	0.0074	0.0074	0.0000	-0.0074
Other mental disorders	B.6.10	0.0000	-0.0005	-0.0005	0.0000	0.0005
Substance use disorders	B.7	0.0749	0.0035	0.0784	0.0606	-0.0177
Alcohol use disorders	B.7.1	0.0092	-0.0029	0.0063	0.0083	0.0020
Drug use disorders	B.7.2	0.0657	0.0064	0.0721	0.0524	-0.0197
Diabetes and kidney diseases	B.8	0.1158	-0.0372	0.0786	0.1445	0.0659
Diabetes mellitus	B.8.1	0.0244	-0.0299	-0.0055	0.0454	0.0510
Chronic kidney disease	B.8.2	0.0714	-0.0073	0.0641	0.0786	0.0146
Acute glomerulonephritis	B.8.3	0.0200	0.0000	0.0201	0.0204	0.0004
Skin and subcutaneous diseases	B.9	0.0163	-0.0129	0.0033	0.0137	0.0103
Dermatitis	B.9.1	0.0000	-0.0016	-0.0016	0.0000	0.0016
Psoriasis	B.9.2	0.0000	0.0102	0.0102	0.0000	-0.0102
Bacterial skin diseases	B.9.3	0.0174	0.0001	0.0175	0.0142	-0.0033
Scabies	B.9.4	0.0000	0.0013	0.0013	0.0000	-0.0013
Fungal skin diseases	B.9.5	0.0000	0.0006	0.0006	0.0000	-0.0006
Viral skin diseases	B.9.6	0.0000	-0.0001	-0.0001	0.0000	0.0001
Acne vulgaris	B.9.7	0.0000	-0.0172	-0.0172	0.0000	0.0172
Alopecia areata	B.9.8	0.0000	0.0000	0.0000	0.0000	0.0000
Pruritus	B.9.9	0.0000	-0.0011	-0.0011	0.0000	0.0011
Urticaria	B.9.10	0.0000	-0.0002	-0.0002	0.0000	0.0002
Decubitus ulcer	B.9.11	-0.0014	-0.0003	-0.0018	-0.0007	0.0011
Other skin and subcutaneous diseases	B.9.12	0.0003	-0.0045	-0.0042	0.0002	0.0044
Sense organ diseases	B.10	0.0000	0.0531	0.0531	0.0000	-0.0531
Blindness and vision loss	B.10.1	0.0000	0.0366	0.0366	0.0000	-0.0366
Age-related and other hearing loss	B.10.2	0.0000	0.0189	0.0189	0.0000	-0.0189
Other sense organ diseases	B.10.3	0.0000	-0.0025	-0.0025	0.0000	0.0025
Musculoskeletal disorders	B.11	-0.0079	0.1428	0.1350	-0.0050	-0.1400
Rheumatoid arthritis	B.11.1	0.0005	-0.0020	-0.0015	0.0013	0.0028
Osteoarthritis	B.11.2	0.0000	-0.0271	-0.0271	0.0000	0.0271
Low back pain	B.11.3	0.0000	0.2812	0.2812	0.0000	-0.2812
Neck pain	B.11.4	0.0000	-0.0130	-0.0130	0.0000	0.0130
Gout	B.11.5	0.0000	-0.0040	-0.0040	0.0000	0.0040
Other musculoskeletal disorders	B.11.6	-0.0084	-0.0924	-0.1007	-0.0063	0.0944

Causes	Level	ΔHALE			ΔLE	ΔLED
		Mortality Effect	Disability Effect	Total Effect	Total Effect	
Other non-communicable diseases	B.12	0.0998	0.2149	0.3147	0.4865	0.1717
Congenital birth defects	B.12.1	0.0535	0.0066	0.0601	0.4178	0.3578
Urinary diseases and male infertility	B.12.2	0.0203	0.0010	0.0213	0.0188	-0.0025
Gynecological diseases	B.12.3	-0.0008	0.1071	0.1063	-0.0008	-0.1071
Hemoglobinopathies and hemolytic anemias	B.12.4	0.0174	0.0781	0.0955	0.0242	-0.0713
Endocrine, metabolic, blood, and immune disorders	B.12.5	0.0093	0.0141	0.0234	0.0161	-0.0073
Oral disorders	B.12.6	0.0000	0.0081	0.0081	0.0000	-0.0081
**Injuries**	C	1.0332	-0.0589	0.9743	1.2129	0.2386
Transport injuries	C.1	0.1388	-0.0472	0.0915	0.1666	0.0750
Road injuries	C.1.1	0.1201	-0.0496	0.0705	0.1463	0.0758
Other transport injuries	C.1.2	0.0187	0.0023	0.0211	0.0202	-0.0008
Unintentional injuries	C.2	0.2472	-0.0235	0.2237	0.5114	0.2878
Falls	C.2.1	-0.0176	-0.0287	-0.0463	0.0173	0.0636
Drowning	C.2.2	0.1806	-0.0003	0.1804	0.2780	0.0976
Fire, heat, and hot substances	C.2.3	0.0200	-0.0016	0.0184	0.0283	0.0099
Poisonings	C.2.4	0.0023	0.0001	0.0024	0.0145	0.0122
Exposure to mechanical forces	C.2.5	0.0060	-0.0069	-0.0009	0.0118	0.0127
Adverse effects of medical treatment	C.2.6	0.0211	0.0000	0.0211	0.0328	0.0117
Animal contact	C.2.7	0.0094	0.0027	0.0121	0.0120	-0.0001
Foreign body	C.2.8	0.0062	0.0079	0.0141	0.0909	0.0768
Environmental heat and cold exposure	C.2.9	0.0104	0.0015	0.0119	0.0144	0.0025
Exposure to forces of nature	C.2.10	0.0028	-0.0012	0.0016	0.0023	0.0008
Other unintentional injuries	C.2.11	0.0062	0.0028	0.0090	0.0091	0.0001
Self-harm and interpersonal violence	C.3	0.6472	0.0119	0.6591	0.5349	-0.1242
Self-harm	C.3.1	0.5954	0.0096	0.6049	0.4753	-0.1296
Interpersonal violence	C.3.2	0.0514	0.0023	0.0537	0.0591	0.0053
Conflict and terrorism	C.3.3	0.0000	0.0000	0.0001	0.0000	0.0000
Executions and police conflict	C.3.4	0.0004	0.0000	0.0004	0.0005	0.0001

Table S7a | Cause-specific effects of mortality and disability (years) on changes in life expectancy (LE) and health-adjusted life expectancy (HALE) for those aged <14 years from 1990 to 2019.

Causes	Level	ΔHALE			ΔLE	ΔLED
		Mortality Effect	Disability Effect	Total Effect	Total Effect	
**Communicable, maternal, neonatal, and nutritional diseases**	A	0.2237	0.0417	0.2654	2.8015	2.5360
HIV/AIDS and sexually transmitted infections	A.1	-0.0012	0.0000	-0.0013	0.0031	0.0044
HIV/AIDS	A.1.1	-0.0014	0.0000	-0.0014	-0.0019	-0.0005
Sexually transmitted infections excluding HIV	A.1.2	0.0002	0.0000	0.0002	0.0050	0.0049
Respiratory infections and tuberculosis	A.2	0.1087	0.0048	0.1135	1.3392	1.2257
Tuberculosis	A.2.1	0.0160	0.0025	0.0185	0.0724	0.0538
Lower respiratory infections	A.2.2	0.0894	0.0012	0.0906	1.2471	1.1565
Upper respiratory infections	A.2.3	0.0032	-0.0002	0.0030	0.0196	0.0166
Otitis media	A.2.4	0.0001	0.0012	0.0013	0.0001	-0.0012
Enteric infections	A.3	0.0267	0.0009	0.0276	0.2552	0.2276
Diarrheal diseases	A.3.1	0.0231	0.0009	0.0240	0.2519	0.2279
Typhoid and paratyphoid	A.3.2	0.0028	0.0000	0.0028	0.0017	-0.0011
Invasive Non-typhoidal Salmonella (iNTS)	A.3.3	0.0005	0.0000	0.0005	0.0006	0.0001
Other intestinal infectious diseases	A.3.4	0.0003	0.0000	0.0003	0.0010	0.0007
Neglected tropical diseases and malaria	A.4	0.0030	0.0147	0.0178	0.0056	-0.0122
Malaria	A.4.1	0.0009	0.0002	0.0011	0.0014	0.0003
Chagas disease	A.4.2	0.0000	0.0000	0.0000	0.0000	0.0000
Leishmaniasis	A.4.3	0.0000	0.0000	0.0000	0.0000	0.0000
African trypanosomiasis	A.4.4	0.0000	0.0000	0.0000	0.0000	0.0000
Schistosomiasis	A.4.5	0.0001	0.0003	0.0005	0.0001	-0.0004
Cysticercosis	A.4.6	0.0000	0.0000	0.0000	0.0000	0.0000
Cystic echinococcosis	A.4.7	0.0000	0.0000	0.0000	0.0000	0.0000
Lymphatic filariasis	A.4.8	0.0000	0.0000	0.0000	0.0000	0.0000
Onchocerciasis	A.4.9	0.0000	0.0000	0.0000	0.0000	0.0000
Trachoma	A.4.10	0.0000	0.0000	0.0000	0.0000	0.0000
Dengue	A.4.11	0.0000	-0.0004	-0.0004	0.0001	0.0005
Yellow fever	A.4.12	0.0000	0.0000	0.0000	0.0000	0.0000
Rabies	A.4.13	0.0011	0.0000	0.0011	0.0016	0.0004
Intestinal nematode infections	A.4.14	0.0005	0.0097	0.0102	0.0012	-0.0090
Food-borne trematodiases	A.4.15	0.0000	0.0016	0.0016	0.0000	-0.0016
Leprosy	A.4.16	0.0000	0.0000	0.0000	0.0000	0.0000
Ebola	A.4.17	0.0000	0.0000	0.0000	0.0000	0.0000
Zika virus	A.4.18	0.0000	0.0000	0.0000	0.0000	0.0000
Guinea worm disease	A.4.19	0.0000	0.0000	0.0000	0.0000	0.0000
Other neglected tropical diseases	A.4.20	0.0002	0.0034	0.0037	0.0011	-0.0025
Other infectious diseases	A.5	0.0712	0.0059	0.0771	0.3741	0.2969
Meningitis	A.5.1	0.0184	0.0012	0.0196	0.0843	0.0647
Encephalitis	A.5.2	0.0040	0.0005	0.0046	0.0176	0.0131
Diphtheria	A.5.3	0.0002	0.0000	0.0002	0.0005	0.0003

Causes	Level	ΔHALE			ΔLE	ΔLED
		Mortality Effect	Disability Effect	Total Effect	Total Effect	
Whooping cough	A.5.4	0.0149	0.0010	0.0159	0.0939	0.0779
Tetanus	A.5.5	0.0021	0.0000	0.0022	0.0487	0.0465
Measles	A.5.6	0.0247	0.0007	0.0253	0.0896	0.0643
Varicella and herpes zoster	A.5.7	0.0015	0.0000	0.0015	0.0041	0.0025
Acute hepatitis	A.5.8	0.0030	0.0004	0.0034	0.0219	0.0185
Other unspecified infectious diseases	A.5.9	0.0023	0.0022	0.0045	0.0135	0.0090
Maternal and neonatal disorders	A.6	0.0087	-0.0233	-0.0147	0.7281	0.7427
Maternal disorders	A.6.1	0.0004	0.0000	0.0004	0.0002	-0.0002
Neonatal disorders	A.6.2	0.0083	-0.0234	-0.0151	0.7279	0.7429
Nutritional deficiencies	A.7	0.0066	0.0387	0.0453	0.0962	0.0509
Protein-energy malnutrition	A.7.1	0.0064	-0.0006	0.0058	0.0932	0.0873
Iodine deficiency	A.7.2	0.0000	0.0007	0.0007	0.0000	-0.0007
Vitamin A deficiency	A.7.3	0.0000	0.0035	0.0035	0.0000	-0.0035
Dietary iron deficiency	A.7.4	0.0000	0.0353	0.0353	0.0000	-0.0353
Other nutritional deficiencies	A.7.5	0.0002	-0.0002	0.0000	0.0030	0.0030
**Non-communicable diseases**	B	0.1495	0.0093	0.1588	0.6919	0.5331
Neoplasms	B.1	0.0471	0.0006	0.0477	0.0844	0.0366
Lip and oral cavity cancer	B.1.1	0.0000	0.0000	0.0000	0.0000	0.0000
Nasopharynx cancer	B.1.2	0.0011	0.0000	0.0011	0.0007	-0.0005
Other pharynx cancer	B.1.3	0.0000	0.0000	0.0000	0.0000	0.0000
Esophageal cancer	B.1.4	0.0000	0.0000	0.0000	0.0000	0.0000
Stomach cancer	B.1.5	0.0000	0.0000	0.0000	0.0000	0.0000
Colon and rectum cancer	B.1.6	0.0003	0.0000	0.0003	0.0002	-0.0001
Liver cancer	B.1.7	0.0025	0.0000	0.0026	0.0042	0.0017
Gallbladder and biliary tract cancer	B.1.8	0.0000	0.0000	0.0000	0.0000	0.0000
Pancreatic cancer	B.1.9	0.0000	0.0000	0.0000	0.0000	0.0000
Larynx cancer	B.1.10	0.0000	0.0000	0.0000	0.0000	0.0000
Tracheal, bronchus, and lung cancer	B.1.11	0.0002	0.0000	0.0002	0.0001	-0.0001
Malignant skin melanoma	B.1.12	0.0000	0.0000	0.0000	0.0000	0.0000
Non-melanoma skin cancer	B.1.13	0.0000	0.0000	0.0000	0.0000	0.0000
Breast cancer	B.1.14	0.0000	0.0000	0.0000	0.0000	0.0000
Cervical cancer	B.1.15	0.0000	0.0000	0.0000	0.0000	0.0000
Uterine cancer	B.1.16	0.0000	0.0000	0.0000	0.0000	0.0000
Ovarian cancer	B.1.17	0.0001	0.0000	0.0001	0.0001	0.0000
Prostate cancer	B.1.18	0.0000	0.0000	0.0000	0.0000	0.0000
Testicular cancer	B.1.19	0.0000	-0.0001	0.0000	0.0001	0.0001
Kidney cancer	B.1.20	0.0001	0.0000	0.0001	0.0008	0.0008
Bladder cancer	B.1.21	0.0000	0.0000	0.0000	0.0000	0.0000
Brain and central nervous system cancer	B.1.22	0.0071	-0.0001	0.0070	0.0120	0.0049
Thyroid cancer	B.1.23	0.0001	0.0000	0.0001	0.0001	0.0000
Mesothelioma	B.1.24	0.0000	0.0000	0.0000	0.0000	0.0000
Hodgkin lymphoma	B.1.25	0.0011	0.0000	0.0011	0.0012	0.0001

Causes	Level	ΔHALE			ΔLE	ΔLED
		Mortality Effect	Disability Effect	Total Effect	Total Effect	
Non-Hodgkin lymphoma	B.1.26	0.0020	0.0000	0.0020	0.0028	0.0009
Multiple myeloma	B.1.27	0.0000	0.0000	0.0000	0.0000	0.0000
Leukemia	B.1.28	0.0262	0.0010	0.0272	0.0494	0.0222
Other malignant neoplasms	B.1.29	0.0062	-0.0002	0.0059	0.0126	0.0066
Other neoplasms	B.1.30	0.0000	0.0000	0.0001	0.0002	0.0001
Cardiovascular diseases	B.2	0.0185	0.0002	0.0187	0.0594	0.0406
Rheumatic heart disease	B.2.1	0.0050	0.0003	0.0053	0.0060	0.0008
Ischemic heart disease	B.2.2	0.0000	0.0000	0.0000	0.0000	0.0000
Stroke	B.2.3	0.0086	0.0002	0.0088	0.0327	0.0239
Hypertensive heart disease	B.2.4	0.0000	0.0000	0.0000	0.0000	0.0000
Non-rheumatic valvular heart disease	B.2.5	0.0000	0.0000	0.0000	0.0000	0.0000
Cardiomyopathy and myocarditis	B.2.6	0.0014	0.0000	0.0014	0.0086	0.0072
Atrial fibrillation and flutter	B.2.8	0.0000	0.0000	0.0000	0.0000	0.0000
Aortic aneurysm	B.2.9	0.0000	0.0000	0.0000	0.0000	0.0000
Peripheral artery disease	B.2.10	0.0000	0.0000	0.0000	0.0000	0.0000
Endocarditis	B.2.11	0.0007	0.0000	0.0007	0.0041	0.0034
Other cardiovascular and circulatory diseases	B.2.12	0.0028	-0.0003	0.0026	0.0080	0.0054
Chronic respiratory diseases	B.3	0.0066	-0.0004	0.0062	0.0095	0.0032
Chronic obstructive pulmonary disease	B.3.1	0.0032	0.0004	0.0037	0.0041	0.0005
Pneumoconiosis	B.3.2	0.0000	0.0000	0.0000	0.0000	0.0000
Asthma	B.3.3	0.0026	0.0001	0.0027	0.0039	0.0011
Interstitial lung disease and pulmonary sarcoidosis	B.3.4	0.0002	0.0000	0.0002	0.0002	0.0001
Other chronic respiratory diseases	B.3.5	0.0006	-0.0009	-0.0003	0.0012	0.0016
Digestive diseases	B.4	0.0150	0.0018	0.0168	0.0567	0.0399
Cirrhosis and other chronic liver diseases	B.4.1	0.0032	0.0001	0.0033	0.0037	0.0003
Upper digestive system diseases	B.4.2	0.0018	0.0009	0.0027	0.0026	-0.0001
Appendicitis	B.4.3	0.0013	0.0000	0.0013	0.0014	0.0000
Paralytic ileus and intestinal obstruction	B.4.4	0.0020	-0.0001	0.0019	0.0284	0.0265
Inguinal, femoral, and abdominal hernia	B.4.5	0.0003	0.0003	0.0005	0.0043	0.0038
Inflammatory bowel disease	B.4.6	0.0009	0.0000	0.0009	0.0027	0.0018
Vascular intestinal disorders	B.4.7	0.0001	0.0000	0.0001	0.0001	0.0000
Gallbladder and biliary diseases	B.4.8	0.0006	0.0003	0.0009	0.0014	0.0005
Pancreatitis	B.4.9	0.0003	0.0000	0.0003	0.0003	-0.0001
Other digestive diseases	B.4.10	0.0046	0.0003	0.0049	0.0118	0.0070
Neurological disorders	B.5	0.0077	-0.0020	0.0057	0.0171	0.0114
Alzheimer's disease and other dementias	B.5.1	0.0000	0.0000	0.0000	0.0000	0.0000
Parkinson's disease	B.5.2	0.0000	0.0000	0.0000	0.0000	0.0000
Idiopathic epilepsy	B.5.3	0.0071	0.0005	0.0075	0.0130	0.0054
Multiple sclerosis	B.5.4	0.0001	0.0000	0.0001	0.0000	0.0000
Motor neuron disease	B.5.5	0.0002	0.0000	0.0002	0.0023	0.0021
Headache disorders	B.5.6	0.0000	-0.0013	-0.0013	0.0000	0.0013
Other neurological disorders	B.5.7	0.0004	-0.0012	-0.0008	0.0017	0.0025

Causes	Level	ΔHALE			ΔLE	ΔLED
		Mortality Effect	Disability Effect	Total Effect	Total Effect	
Mental disorders	B.6	0.0000	-0.0019	-0.0019	0.0000	0.0019
Schizophrenia	B.6.1	0.0000	0.0000	0.0000	0.0000	0.0000
Depressive disorders	B.6.2	0.0000	0.0001	0.0001	0.0000	-0.0001
Bipolar disorder	B.6.3	0.0000	0.0000	0.0000	0.0000	0.0000
Anxiety disorders	B.6.4	0.0000	-0.0012	-0.0012	0.0000	0.0012
Eating disorders	B.6.5	0.0000	-0.0003	-0.0003	0.0000	0.0003
Autism spectrum disorders	B.6.6	0.0000	-0.0005	-0.0005	0.0000	0.0005
Attention-deficit/hyperactivity disorder	B.6.7	0.0000	-0.0011	-0.0011	0.0000	0.0011
Conduct disorder	B.6.8	0.0000	-0.0010	-0.0010	0.0000	0.0010
Idiopathic developmental intellectual disability	B.6.9	0.0000	0.0022	0.0022	0.0000	-0.0022
Other mental disorders	B.6.10	0.0000	0.0000	0.0000	0.0000	0.0000
Substance use disorders	B.7	0.0000	0.0000	0.0000	0.0000	0.0000
Alcohol use disorders	B.7.1	0.0000	0.0001	0.0001	0.0000	-0.0001
Drug use disorders	B.7.2	0.0000	0.0000	0.0000	0.0000	0.0000
Diabetes and kidney diseases	B.8	0.0086	0.0001	0.0087	0.0149	0.0062
Diabetes mellitus	B.8.1	0.0010	-0.0001	0.0009	0.0021	0.0012
Chronic kidney disease	B.8.2	0.0052	0.0002	0.0054	0.0093	0.0039
Acute glomerulonephritis	B.8.3	0.0024	0.0000	0.0024	0.0035	0.0012
Skin and subcutaneous diseases	B.9	0.0008	-0.0037	-0.0029	0.0035	0.0064
Dermatitis	B.9.1	0.0000	-0.0001	-0.0001	0.0000	0.0001
Psoriasis	B.9.2	0.0000	0.0006	0.0006	0.0000	-0.0006
Bacterial skin diseases	B.9.3	0.0008	0.0000	0.0008	0.0035	0.0027
Scabies	B.9.4	0.0000	0.0004	0.0004	0.0000	-0.0004
Fungal skin diseases	B.9.5	0.0000	0.0003	0.0003	0.0000	-0.0003
Viral skin diseases	B.9.6	0.0000	-0.0001	-0.0001	0.0000	0.0001
Acne vulgaris	B.9.7	0.0000	-0.0041	-0.0041	0.0000	0.0041
Alopecia areata	B.9.8	0.0000	0.0000	0.0000	0.0000	0.0000
Pruritus	B.9.9	0.0000	-0.0001	-0.0001	0.0000	0.0001
Urticaria	B.9.10	0.0000	0.0000	0.0000	0.0000	0.0000
Decubitus ulcer	B.9.11	0.0000	0.0000	0.0000	0.0000	0.0000
Other skin and subcutaneous diseases	B.9.12	0.0000	-0.0006	-0.0006	0.0000	0.0006
Sense organ diseases	B.10	0.0000	0.0001	0.0001	0.0000	-0.0001
Blindness and vision loss	B.10.1	0.0000	-0.0003	-0.0003	0.0000	0.0003
Age-related and other hearing loss	B.10.2	0.0000	0.0005	0.0005	0.0000	-0.0005
Other sense organ diseases	B.10.3	0.0000	-0.0001	-0.0001	0.0000	0.0001
Musculoskeletal disorders	B.11	0.0005	0.0000	0.0006	0.0003	-0.0003
Rheumatoid arthritis	B.11.1	0.0001	0.0000	0.0001	0.0001	0.0000
Osteoarthritis	B.11.2	0.0000	0.0000	0.0000	0.0000	0.0000
Low back pain	B.11.3	0.0000	0.0001	0.0001	0.0000	-0.0001
Neck pain	B.11.4	0.0000	0.0000	0.0000	0.0000	0.0000
Gout	B.11.5	0.0000	0.0000	0.0000	0.0000	0.0000
Other musculoskeletal disorders	B.11.6	0.0004	-0.0001	0.0004	0.0003	-0.0001

Causes	Level	ΔHALE			ΔLE	ΔLED
		Mortality Effect	Disability Effect	Total Effect	Total Effect	
Other non-communicable diseases	B.12	0.0446	0.0143	0.0589	0.4462	0.3873
Congenital birth defects	B.12.1	0.0366	0.0018	0.0384	0.4145	0.3761
Urinary diseases and male infertility	B.12.2	0.0010	0.0000	0.0010	0.0018	0.0009
Gynecological diseases	B.12.3	0.0000	0.0004	0.0004	0.0000	-0.0004
Hemoglobinopathies and hemolytic anemias	B.12.4	0.0051	0.0102	0.0153	0.0131	-0.0021
Endocrine, metabolic, blood, and immune disorders	B.12.5	0.0019	0.0013	0.0033	0.0083	0.0050
Oral disorders	B.12.6	0.0000	0.0004	0.0004	0.0000	-0.0004
**Injuries**	C	0.3093	0.0035	0.3128	0.5921	0.2793
Transport injuries	C.1	0.0604	-0.0002	0.0602	0.1000	0.0398
Road injuries	C.1.1	0.0556	-0.0004	0.0552	0.0919	0.0366
Other transport injuries	C.1.2	0.0047	0.0002	0.0050	0.0081	0.0031
Unintentional injuries	C.2	0.2161	0.0033	0.2195	0.4570	0.2376
Falls	C.2.1	0.0092	0.0014	0.0106	0.0249	0.0144
Drowning	C.2.2	0.1698	0.0002	0.1700	0.2675	0.0975
Fire, heat, and hot substances	C.2.3	0.0065	0.0000	0.0065	0.0192	0.0127
Poisonings	C.2.4	0.0072	0.0000	0.0072	0.0197	0.0125
Exposure to mechanical forces	C.2.5	0.0050	0.0002	0.0052	0.0121	0.0069
Adverse effects of medical treatment	C.2.6	0.0042	0.0000	0.0042	0.0167	0.0125
Animal contact	C.2.7	0.0046	0.0002	0.0048	0.0081	0.0033
Foreign body	C.2.8	0.0047	0.0010	0.0056	0.0742	0.0686
Environmental heat and cold exposure	C.2.9	0.0014	0.0003	0.0016	0.0081	0.0065
Exposure to forces of nature	C.2.10	0.0008	0.0000	0.0008	0.0008	-0.0001
Other unintentional injuries	C.2.11	0.0027	0.0002	0.0029	0.0056	0.0027
Self-harm and interpersonal violence	C.3	0.0328	0.0004	0.0331	0.0351	0.0019
Self-harm	C.3.1	0.0178	0.0000	0.0179	0.0083	-0.0096
Interpersonal violence	C.3.2	0.0148	0.0003	0.0151	0.0265	0.0114
Conflict and terrorism	C.3.3	0.0000	0.0000	0.0000	0.0000	0.0000
Executions and police conflict	C.3.4	0.0001	0.0000	0.0002	0.0003	0.0001

Table S7b | Cause-specific effects of mortality and disability (years) on changes in life expectancy (LE) and health-adjusted life expectancy (HALE) for those aged 15-44 years from 1990 to 2019.

Causes	Level	ΔHALE			ΔLE	ΔLED
		Mortality Effect	Disability Effect	Total Effect	Total Effect	
**Communicable, maternal, neonatal, and nutritional diseases**	A	0.2484	0.0584	0.3068	0.1508	-0.1560
HIV/AIDS and sexually transmitted infections	A.1	-0.0321	-0.0010	-0.0331	-0.0203	0.0127
HIV/AIDS	A.1.1	-0.0329	-0.0012	-0.0342	-0.0209	0.0133
Sexually transmitted infections excluding HIV	A.1.2	0.0008	0.0003	0.0011	0.0005	-0.0006
Respiratory infections and tuberculosis	A.2	0.1446	0.0097	0.1543	0.0915	-0.0628
Tuberculosis	A.2.1	0.1023	0.0056	0.1078	0.0660	-0.0419
Lower respiratory infections	A.2.2	0.0391	0.0004	0.0396	0.0235	-0.0160
Upper respiratory infections	A.2.3	0.0031	-0.0001	0.0030	0.0019	-0.0011
Otitis media	A.2.4	0.0002	0.0037	0.0039	0.0001	-0.0038
Enteric infections	A.3	0.0148	0.0002	0.0150	0.0088	-0.0062
Diarrheal diseases	A.3.1	0.0111	0.0002	0.0112	0.0067	-0.0045
Typhoid and paratyphoid	A.3.2	0.0030	0.0000	0.0030	0.0016	-0.0014
Invasive Non-typhoidal Salmonella (iNTS)	A.3.3	0.0006	0.0000	0.0006	0.0003	-0.0002
Other intestinal infectious diseases	A.3.4	0.0001	0.0000	0.0001	0.0001	0.0000
Neglected tropical diseases and malaria	A.4	0.0053	0.0370	0.0423	0.0032	-0.0391
Malaria	A.4.1	0.0030	0.0003	0.0033	0.0017	-0.0016
Chagas disease	A.4.2	0.0000	0.0000	0.0000	0.0000	0.0000
Leishmaniasis	A.4.3	0.0000	0.0000	0.0000	0.0000	0.0000
African trypanosomiasis	A.4.4	0.0000	0.0000	0.0000	0.0000	0.0000
Schistosomiasis	A.4.5	0.0011	0.0027	0.0038	0.0008	-0.0031
Cysticercosis	A.4.6	0.0001	0.0011	0.0012	0.0001	-0.0012
Cystic echinococcosis	A.4.7	0.0000	0.0000	0.0000	0.0000	0.0000
Lymphatic filariasis	A.4.8	0.0000	0.0000	0.0000	0.0000	0.0000
Onchocerciasis	A.4.9	0.0000	0.0000	0.0000	0.0000	0.0000
Trachoma	A.4.10	0.0000	0.0001	0.0001	0.0000	-0.0001
Dengue	A.4.11	0.0000	-0.0012	-0.0012	0.0000	0.0012
Yellow fever	A.4.12	0.0000	0.0000	0.0000	0.0000	0.0000
Rabies	A.4.13	0.0006	0.0000	0.0006	0.0004	-0.0002
Intestinal nematode infections	A.4.14	0.0001	0.0199	0.0200	0.0000	-0.0199
Food-borne trematodiases	A.4.15	0.0000	0.0119	0.0119	0.0000	-0.0119
Leprosy	A.4.16	0.0000	0.0000	0.0000	0.0000	0.0000
Ebola	A.4.17	0.0000	0.0000	0.0000	0.0000	0.0000
Zika virus	A.4.18	0.0000	0.0000	0.0000	0.0000	0.0000
Guinea worm disease	A.4.19	0.0000	0.0000	0.0000	0.0000	0.0000
Other neglected tropical diseases	A.4.20	0.0004	0.0022	0.0026	0.0003	-0.0024
Other infectious diseases	A.5	0.0486	0.0067	0.0553	0.0301	-0.0251
Meningitis	A.5.1	0.0129	0.0022	0.0151	0.0075	-0.0076
Encephalitis	A.5.2	0.0020	0.0017	0.0037	0.0011	-0.0026
Diphtheria	A.5.3	0.0001	0.0000	0.0001	0.0000	0.0000

Causes	Level	ΔHALE			ΔLE	ΔLED
		Mortality Effect	Disability Effect	Total Effect	Total Effect	
Whooping cough	A.5.4	0.0016	0.0000	0.0016	0.0009	-0.0007
Tetanus	A.5.5	0.0049	0.0000	0.0049	0.0032	-0.0017
Measles	A.5.6	0.0028	0.0000	0.0028	0.0015	-0.0013
Varicella and herpes zoster	A.5.7	0.0010	0.0000	0.0010	0.0006	-0.0004
Acute hepatitis	A.5.8	0.0208	0.0005	0.0213	0.0137	-0.0075
Other unspecified infectious diseases	A.5.9	0.0026	0.0022	0.0048	0.0016	-0.0032
Maternal and neonatal disorders	A.6	0.0624	-0.0372	0.0251	0.0345	0.0094
Maternal disorders	A.6.1	0.0624	0.0039	0.0663	0.0345	-0.0317
Neonatal disorders	A.6.2	0.0000	-0.0412	-0.0412	0.0000	0.0412
Nutritional deficiencies	A.7	0.0049	0.0430	0.0479	0.0029	-0.0450
Protein-energy malnutrition	A.7.1	0.0048	-0.0056	-0.0009	0.0029	0.0037
Iodine deficiency	A.7.2	0.0000	0.0014	0.0014	0.0000	-0.0014
Vitamin A deficiency	A.7.3	0.0000	0.0001	0.0001	0.0000	-0.0001
Dietary iron deficiency	A.7.4	0.0000	0.0487	0.0487	0.0000	-0.0487
Other nutritional deficiencies	A.7.5	0.0001	-0.0015	-0.0014	0.0001	0.0014
**Non-communicable diseases**	B	0.7465	0.1433	0.8898	0.5117	-0.3781
Neoplasms	B.1	0.2084	-0.0052	0.2032	0.1505	-0.0528
Lip and oral cavity cancer	B.1.1	0.0005	0.0000	0.0005	0.0003	-0.0002
Nasopharynx cancer	B.1.2	0.0117	-0.0006	0.0111	0.0081	-0.0030
Other pharynx cancer	B.1.3	0.0006	0.0000	0.0006	0.0004	-0.0001
Esophageal cancer	B.1.4	0.0116	0.0001	0.0117	0.0096	-0.0020
Stomach cancer	B.1.5	0.0393	-0.0001	0.0391	0.0297	-0.0095
Colon and rectum cancer	B.1.6	-0.0038	-0.0009	-0.0047	-0.0030	0.0017
Liver cancer	B.1.7	0.0851	0.0003	0.0854	0.0634	-0.0219
Gallbladder and biliary tract cancer	B.1.8	0.0005	0.0000	0.0005	0.0003	-0.0002
Pancreatic cancer	B.1.9	-0.0043	0.0000	-0.0043	-0.0034	0.0009
Larynx cancer	B.1.10	0.0014	0.0000	0.0014	0.0010	-0.0003
Tracheal, bronchus, and lung cancer	B.1.11	0.0130	0.0000	0.0130	0.0099	-0.0030
Malignant skin melanoma	B.1.12	0.0005	-0.0001	0.0005	0.0004	-0.0001
Non-melanoma skin cancer	B.1.13	0.0000	0.0000	0.0000	0.0000	0.0000
Breast cancer	B.1.14	0.0052	-0.0011	0.0041	0.0041	0.0000
Cervical cancer	B.1.15	0.0020	-0.0003	0.0017	0.0011	-0.0005
Uterine cancer	B.1.16	0.0027	-0.0001	0.0026	0.0019	-0.0006
Ovarian cancer	B.1.17	0.0000	-0.0001	-0.0001	-0.0001	0.0000
Prostate cancer	B.1.18	0.0002	0.0000	0.0001	0.0001	0.0000
Testicular cancer	B.1.19	0.0002	-0.0002	0.0001	0.0001	0.0001
Kidney cancer	B.1.20	-0.0023	-0.0002	-0.0025	-0.0016	0.0009
Bladder cancer	B.1.21	0.0015	-0.0001	0.0014	0.0010	-0.0004
Brain and central nervous system cancer	B.1.22	0.0038	-0.0003	0.0034	0.0026	-0.0008
Thyroid cancer	B.1.23	0.0006	-0.0001	0.0004	0.0004	-0.0001
Mesothelioma	B.1.24	0.0000	0.0000	0.0000	0.0000	0.0000
Hodgkin lymphoma	B.1.25	0.0040	0.0000	0.0040	0.0024	-0.0016

Causes	Level	ΔHALE			ΔLE	ΔLED
		Mortality Effect	Disability Effect	Total Effect	Total Effect	
Non-Hodgkin lymphoma	B.1.26	-0.0022	-0.0003	-0.0025	-0.0014	0.0011
Multiple myeloma	B.1.27	-0.0009	0.0000	-0.0009	-0.0005	0.0004
Leukemia	B.1.28	0.0301	-0.0003	0.0298	0.0183	-0.0115
Other malignant neoplasms	B.1.29	0.0077	-0.0006	0.0071	0.0051	-0.0020
Other neoplasms	B.1.30	-0.0003	0.0000	-0.0002	-0.0002	0.0001
Cardiovascular diseases	B.2	0.1963	0.0021	0.1985	0.1364	-0.0620
Rheumatic heart disease	B.2.1	0.0562	0.0014	0.0576	0.0377	-0.0199
Ischemic heart disease	B.2.2	0.0264	-0.0003	0.0261	0.0175	-0.0086
Stroke	B.2.3	0.0899	0.0023	0.0921	0.0650	-0.0272
Hypertensive heart disease	B.2.4	0.0163	-0.0001	0.0162	0.0116	-0.0047
Non-rheumatic valvular heart disease	B.2.5	0.0009	0.0000	0.0009	0.0006	-0.0004
Cardiomyopathy and myocarditis	B.2.6	0.0016	0.0000	0.0015	0.0010	-0.0006
Atrial fibrillation and flutter	B.2.8	0.0001	0.0000	0.0000	0.0000	0.0000
Aortic aneurysm	B.2.9	-0.0002	0.0000	-0.0002	-0.0002	0.0000
Peripheral artery disease	B.2.10	0.0000	0.0000	0.0000	0.0000	0.0000
Endocarditis	B.2.11	0.0017	0.0000	0.0017	0.0010	-0.0007
Other cardiovascular and circulatory diseases	B.2.12	0.0034	-0.0010	0.0024	0.0022	-0.0002
Chronic respiratory diseases	B.3	0.0747	0.0112	0.0859	0.0524	-0.0335
Chronic obstructive pulmonary disease	B.3.1	0.0605	0.0097	0.0702	0.0430	-0.0272
Pneumoconiosis	B.3.2	0.0039	0.0010	0.0049	0.0028	-0.0021
Asthma	B.3.3	0.0101	0.0012	0.0114	0.0065	-0.0049
Interstitial lung disease and pulmonary sarcoidosis	B.3.4	0.0002	0.0001	0.0003	0.0001	-0.0002
Other chronic respiratory diseases	B.3.5	0.0000	-0.0009	-0.0009	0.0000	0.0009
Digestive diseases	B.4	0.1235	0.0076	0.1311	0.0854	-0.0456
Cirrhosis and other chronic liver diseases	B.4.1	0.0782	0.0006	0.0788	0.0556	-0.0233
Upper digestive system diseases	B.4.2	0.0223	0.0037	0.0260	0.0153	-0.0107
Appendicitis	B.4.3	0.0030	0.0000	0.0030	0.0018	-0.0012
Paralytic ileus and intestinal obstruction	B.4.4	0.0040	0.0000	0.0040	0.0024	-0.0015
Inguinal, femoral, and abdominal hernia	B.4.5	0.0003	0.0007	0.0010	0.0002	-0.0008
Inflammatory bowel disease	B.4.6	0.0014	-0.0010	0.0004	0.0009	0.0005
Vascular intestinal disorders	B.4.7	0.0002	0.0000	0.0002	0.0001	-0.0001
Gallbladder and biliary diseases	B.4.8	0.0036	0.0034	0.0070	0.0025	-0.0046
Pancreatitis	B.4.9	0.0048	0.0001	0.0049	0.0031	-0.0018
Other digestive diseases	B.4.10	0.0057	0.0002	0.0059	0.0037	-0.0022
Neurological disorders	B.5	0.0178	-0.0131	0.0047	0.0106	0.0059
Alzheimer's disease and other dementias	B.5.1	0.0000	0.0000	0.0000	0.0000	0.0000
Parkinson's disease	B.5.2	0.0003	-0.0001	0.0002	0.0003	0.0000
Idiopathic epilepsy	B.5.3	0.0181	0.0000	0.0180	0.0105	-0.0075
Multiple sclerosis	B.5.4	0.0004	0.0000	0.0004	0.0003	-0.0001
Motor neuron disease	B.5.5	0.0003	0.0000	0.0003	0.0002	-0.0001
Headache disorders	B.5.6	0.0000	-0.0120	-0.0120	0.0000	0.0120
Other neurological disorders	B.5.7	-0.0013	-0.0010	-0.0022	-0.0006	0.0016

Causes	Level	ΔHALE			ΔLE	ΔLED
		Mortality Effect	Disability Effect	Total Effect	Total Effect	
Mental disorders	B.6	-0.0001	0.0591	0.0590	0.0000	-0.0591
Schizophrenia	B.6.1	0.0000	-0.0035	-0.0035	0.0000	0.0035
Depressive disorders	B.6.2	0.0000	0.0503	0.0503	0.0000	-0.0503
Bipolar disorder	B.6.3	0.0000	-0.0001	-0.0001	0.0000	0.0001
Anxiety disorders	B.6.4	0.0000	0.0162	0.0162	0.0000	-0.0162
Eating disorders	B.6.5	-0.0001	-0.0059	-0.0060	0.0000	0.0059
Autism spectrum disorders	B.6.6	0.0000	-0.0007	-0.0007	0.0000	0.0007
Attention-deficit/hyperactivity disorder	B.6.7	0.0000	0.0000	0.0000	0.0000	0.0000
Conduct disorder	B.6.8	0.0000	-0.0004	-0.0004	0.0000	0.0004
Idiopathic developmental intellectual disability	B.6.9	0.0000	0.0033	0.0033	0.0000	-0.0033
Other mental disorders	B.6.10	0.0000	-0.0001	-0.0001	0.0000	0.0001
Substance use disorders	B.7	0.0456	0.0150	0.0606	0.0263	-0.0343
Alcohol use disorders	B.7.1	-0.0019	0.0042	0.0022	-0.0015	-0.0037
Drug use disorders	B.7.2	0.0475	0.0108	0.0584	0.0278	-0.0306
Diabetes and kidney diseases	B.8	0.0550	-0.0145	0.0405	0.0352	-0.0053
Diabetes mellitus	B.8.1	0.0060	-0.0149	-0.0089	0.0040	0.0129
Chronic kidney disease	B.8.2	0.0405	0.0004	0.0409	0.0259	-0.0150
Acute glomerulonephritis	B.8.3	0.0085	0.0000	0.0085	0.0053	-0.0033
Skin and subcutaneous diseases	B.9	0.0020	-0.0073	-0.0053	0.0013	0.0065
Dermatitis	B.9.1	0.0000	-0.0007	-0.0007	0.0000	0.0007
Psoriasis	B.9.2	0.0000	0.0035	0.0035	0.0000	-0.0035
Bacterial skin diseases	B.9.3	0.0022	0.0000	0.0022	0.0014	-0.0008
Scabies	B.9.4	0.0000	0.0008	0.0008	0.0000	-0.0008
Fungal skin diseases	B.9.5	0.0000	0.0002	0.0002	0.0000	-0.0002
Viral skin diseases	B.9.6	0.0000	-0.0001	-0.0001	0.0000	0.0001
Acne vulgaris	B.9.7	0.0000	-0.0091	-0.0091	0.0000	0.0091
Alopecia areata	B.9.8	0.0000	0.0000	0.0000	0.0000	0.0000
Pruritus	B.9.9	0.0000	-0.0003	-0.0003	0.0000	0.0003
Urticaria	B.9.10	0.0000	0.0000	0.0000	0.0000	0.0000
Decubitus ulcer	B.9.11	-0.0002	0.0000	-0.0002	-0.0001	0.0000
Other skin and subcutaneous diseases	B.9.12	0.0000	-0.0015	-0.0015	0.0000	0.0015
Sense organ diseases	B.10	0.0000	0.0023	0.0023	0.0000	-0.0023
Blindness and vision loss	B.10.1	0.0000	0.0011	0.0011	0.0000	-0.0011
Age-related and other hearing loss	B.10.2	0.0000	0.0015	0.0015	0.0000	-0.0015
Other sense organ diseases	B.10.3	0.0000	-0.0003	-0.0003	0.0000	0.0003
Musculoskeletal disorders	B.11	-0.0014	0.0295	0.0281	-0.0007	-0.0288
Rheumatoid arthritis	B.11.1	0.0005	-0.0003	0.0002	0.0004	0.0002
Osteoarthritis	B.11.2	0.0000	-0.0007	-0.0007	0.0000	0.0007
Low back pain	B.11.3	0.0000	0.0565	0.0565	0.0000	-0.0565
Neck pain	B.11.4	0.0000	0.0055	0.0055	0.0000	-0.0055
Gout	B.11.5	0.0000	-0.0006	-0.0006	0.0000	0.0006
Other musculoskeletal disorders	B.11.6	-0.0019	-0.0309	-0.0328	-0.0011	0.0318

Causes	Level	ΔHALE			ΔLE	ΔLED
		Mortality Effect	Disability Effect	Total Effect	Total Effect	
Other non-communicable diseases	B.12	0.0246	0.0566	0.0811	0.0143	-0.0668
Congenital birth defects	B.12.1	0.0125	0.0034	0.0159	0.0069	-0.0090
Urinary diseases and male infertility	B.12.2	0.0042	0.0002	0.0043	0.0027	-0.0017
Gynecological diseases	B.12.3	-0.0001	0.0235	0.0235	-0.0001	-0.0235
Hemoglobinopathies and hemolytic anemias	B.12.4	0.0054	0.0219	0.0273	0.0032	-0.0241
Endocrine, metabolic, blood, and immune disorders	B.12.5	0.0026	0.0062	0.0088	0.0016	-0.0072
Oral disorders	B.12.6	0.0000	0.0014	0.0014	0.0000	-0.0014
**Injuries**	C	0.6654	0.0103	0.6757	0.3942	-0.2815
Transport injuries	C.1	0.1276	-0.0079	0.1197	0.0791	-0.0405
Road injuries	C.1.1	0.1069	-0.0092	0.0978	0.0668	-0.0310
Other transport injuries	C.1.2	0.0207	0.0012	0.0219	0.0124	-0.0095
Unintentional injuries	C.2	0.1313	0.0141	0.1454	0.0776	-0.0678
Falls	C.2.1	0.0084	0.0100	0.0184	0.0052	-0.0132
Drowning	C.2.2	0.0650	0.0007	0.0656	0.0371	-0.0285
Fire, heat, and hot substances	C.2.3	0.0100	-0.0002	0.0097	0.0059	-0.0038
Poisonings	C.2.4	0.0018	0.0000	0.0018	0.0012	-0.0006
Exposure to mechanical forces	C.2.5	0.0188	-0.0018	0.0171	0.0114	-0.0057
Adverse effects of medical treatment	C.2.6	0.0078	0.0000	0.0078	0.0047	-0.0031
Animal contact	C.2.7	0.0070	0.0009	0.0079	0.0044	-0.0036
Foreign body	C.2.8	0.0003	0.0035	0.0037	0.0002	-0.0036
Environmental heat and cold exposure	C.2.9	0.0092	0.0007	0.0099	0.0057	-0.0042
Exposure to forces of nature	C.2.10	0.0021	-0.0006	0.0015	0.0012	-0.0003
Other unintentional injuries	C.2.11	0.0010	0.0010	0.0020	0.0007	-0.0013
Self-harm and interpersonal violence	C.3	0.4065	0.0041	0.4107	0.2374	-0.1732
Self-harm	C.3.1	0.3282	0.0021	0.3303	0.1925	-0.1378
Interpersonal violence	C.3.2	0.0676	0.0020	0.0696	0.0389	-0.0307
Conflict and terrorism	C.3.3	0.0001	0.0000	0.0001	0.0000	-0.0001
Executions and police conflict	C.3.4	0.0107	0.0000	0.0107	0.0060	-0.0047

Table S7c | Cause-specific effects of mortality and disability (years) on changes in life expectancy (LE) and health-adjusted life expectancy (HALE) for those aged 45-64 years from 1990 to 2019.

Causes	Level	ΔHALE			ΔLE	ΔLED
		Mortality Effect	Disability Effect	Total Effect	Total Effect	
**Communicable, maternal, neonatal, and nutritional diseases**	A	0.1534	0.0581	0.2115	0.1911	-0.0204
HIV/AIDS and sexually transmitted infections	A.1	-0.0094	-0.0010	-0.0104	-0.0112	-0.0008
HIV/AIDS	A.1.1	-0.0100	-0.0011	-0.0111	-0.0119	-0.0008
Sexually transmitted infections excluding HIV	A.1.2	0.0006	0.0001	0.0007	0.0008	0.0001
Respiratory infections and tuberculosis	A.2	0.1291	0.0122	0.1413	0.1608	0.0195
Tuberculosis	A.2.1	0.0962	0.0101	0.1063	0.1197	0.0134
Lower respiratory infections	A.2.2	0.0307	0.0005	0.0312	0.0384	0.0072
Upper respiratory infections	A.2.3	0.0020	-0.0001	0.0020	0.0025	0.0006
Otitis media	A.2.4	0.0001	0.0016	0.0017	0.0001	-0.0016
Enteric infections	A.3	0.0064	-0.0012	0.0052	0.0079	0.0027
Diarrheal diseases	A.3.1	0.0061	-0.0012	0.0049	0.0075	0.0026
Typhoid and paratyphoid	A.3.2	0.0001	0.0000	0.0001	0.0002	0.0000
Invasive Non-typhoidal Salmonella (iNTS)	A.3.3	0.0001	0.0000	0.0001	0.0002	0.0000
Other intestinal infectious diseases	A.3.4	0.0000	0.0000	0.0000	0.0000	0.0000
Neglected tropical diseases and malaria	A.4	0.0027	0.0280	0.0307	0.0033	-0.0274
Malaria	A.4.1	0.0005	0.0001	0.0007	0.0006	0.0000
Chagas disease	A.4.2	0.0000	0.0000	0.0000	0.0000	0.0000
Leishmaniasis	A.4.3	0.0000	0.0000	0.0000	0.0000	0.0000
African trypanosomiasis	A.4.4	0.0000	0.0000	0.0000	0.0000	0.0000
Schistosomiasis	A.4.5	0.0015	0.0009	0.0024	0.0019	-0.0005
Cysticercosis	A.4.6	0.0000	0.0021	0.0022	0.0001	-0.0021
Cystic echinococcosis	A.4.7	0.0000	0.0000	0.0000	0.0000	0.0000
Lymphatic filariasis	A.4.8	0.0000	0.0000	0.0000	0.0000	0.0000
Onchocerciasis	A.4.9	0.0000	0.0000	0.0000	0.0000	0.0000
Trachoma	A.4.10	0.0000	0.0009	0.0009	0.0000	-0.0009
Dengue	A.4.11	0.0000	-0.0003	-0.0003	0.0000	0.0003
Yellow fever	A.4.12	0.0000	0.0000	0.0000	0.0000	0.0000
Rabies	A.4.13	0.0003	0.0000	0.0003	0.0004	0.0001
Intestinal nematode infections	A.4.14	0.0000	0.0071	0.0071	0.0000	-0.0071
Food-borne trematodiases	A.4.15	0.0000	0.0154	0.0154	0.0000	-0.0154
Leprosy	A.4.16	0.0000	0.0000	0.0000	0.0000	0.0000
Ebola	A.4.17	0.0000	0.0000	0.0000	0.0000	0.0000
Zika virus	A.4.18	0.0000	0.0000	0.0000	0.0000	0.0000
Guinea worm disease	A.4.19	0.0000	0.0000	0.0000	0.0000	0.0000
Other neglected tropical diseases	A.4.20	0.0003	0.0018	0.0021	0.0004	-0.0017
Other infectious diseases	A.5	0.0215	0.0037	0.0252	0.0264	0.0013
Meningitis	A.5.1	0.0031	0.0010	0.0041	0.0038	-0.0002
Encephalitis	A.5.2	0.0004	0.0006	0.0011	0.0005	-0.0005
Diphtheria	A.5.3	0.0000	0.0000	0.0000	0.0000	0.0000

Causes	Level	ΔHALE			ΔLE	ΔLED
		Mortality Effect	Disability Effect	Total Effect	Total Effect	
Whooping cough	A.5.4	0.0002	0.0000	0.0002	0.0002	0.0000
Tetanus	A.5.5	0.0029	0.0000	0.0029	0.0035	0.0006
Measles	A.5.6	0.0001	0.0000	0.0001	0.0001	0.0000
Varicella and herpes zoster	A.5.7	0.0003	0.0000	0.0003	0.0004	0.0001
Acute hepatitis	A.5.8	0.0133	0.0003	0.0136	0.0163	0.0028
Other unspecified infectious diseases	A.5.9	0.0012	0.0018	0.0029	0.0014	-0.0015
Maternal and neonatal disorders	A.6	0.0007	-0.0230	-0.0223	0.0007	0.0230
Maternal disorders	A.6.1	0.0007	0.0000	0.0007	0.0007	0.0001
Neonatal disorders	A.6.2	0.0000	-0.0230	-0.0230	0.0000	0.0230
Nutritional deficiencies	A.7	0.0025	0.0394	0.0419	0.0031	-0.0388
Protein-energy malnutrition	A.7.1	0.0024	-0.0041	-0.0017	0.0030	0.0047
Iodine deficiency	A.7.2	0.0000	0.0004	0.0004	0.0000	-0.0004
Vitamin A deficiency	A.7.3	0.0000	0.0000	0.0000	0.0000	0.0000
Dietary iron deficiency	A.7.4	0.0000	0.0435	0.0435	0.0000	-0.0435
Other nutritional deficiencies	A.7.5	0.0001	-0.0003	-0.0003	0.0001	0.0003
**Non-communicable diseases**	B	1.4898	0.0612	1.5510	1.8684	0.3174
Neoplasms	B.1	0.3848	-0.0114	0.3734	0.4800	0.1066
Lip and oral cavity cancer	B.1.1	-0.0010	-0.0002	-0.0011	-0.0013	-0.0001
Nasopharynx cancer	B.1.2	0.0131	-0.0008	0.0123	0.0162	0.0039
Other pharynx cancer	B.1.3	0.0007	0.0000	0.0007	0.0009	0.0002
Esophageal cancer	B.1.4	0.0679	0.0008	0.0687	0.0856	0.0169
Stomach cancer	B.1.5	0.1249	0.0005	0.1254	0.1571	0.0317
Colon and rectum cancer	B.1.6	-0.0097	-0.0036	-0.0133	-0.0121	0.0012
Liver cancer	B.1.7	0.1477	0.0016	0.1493	0.1829	0.0337
Gallbladder and biliary tract cancer	B.1.8	-0.0002	0.0000	-0.0002	-0.0003	-0.0001
Pancreatic cancer	B.1.9	-0.0105	-0.0001	-0.0107	-0.0131	-0.0024
Larynx cancer	B.1.10	0.0036	-0.0002	0.0034	0.0046	0.0011
Tracheal, bronchus, and lung cancer	B.1.11	0.0192	-0.0004	0.0188	0.0238	0.0050
Malignant skin melanoma	B.1.12	0.0005	-0.0001	0.0004	0.0006	0.0002
Non-melanoma skin cancer	B.1.13	0.0000	0.0000	-0.0001	0.0000	0.0000
Breast cancer	B.1.14	0.0044	-0.0032	0.0012	0.0051	0.0040
Cervical cancer	B.1.15	0.0015	-0.0004	0.0011	0.0019	0.0008
Uterine cancer	B.1.16	0.0051	-0.0003	0.0048	0.0064	0.0016
Ovarian cancer	B.1.17	-0.0040	-0.0003	-0.0043	-0.0050	-0.0008
Prostate cancer	B.1.18	0.0015	-0.0006	0.0009	0.0020	0.0010
Testicular cancer	B.1.19	0.0001	-0.0001	0.0000	0.0001	0.0001
Kidney cancer	B.1.20	-0.0029	-0.0004	-0.0033	-0.0036	-0.0004
Bladder cancer	B.1.21	0.0021	-0.0005	0.0017	0.0027	0.0010
Brain and central nervous system cancer	B.1.22	0.0037	-0.0001	0.0035	0.0045	0.0010
Thyroid cancer	B.1.23	0.0005	-0.0002	0.0002	0.0006	0.0003
Mesothelioma	B.1.24	-0.0001	0.0000	-0.0001	-0.0002	0.0000
Hodgkin lymphoma	B.1.25	0.0022	0.0000	0.0022	0.0027	0.0006

Causes	Level	ΔHALE			ΔLE	ΔLED
		Mortality Effect	Disability Effect	Total Effect	Total Effect	
Non-Hodgkin lymphoma	B.1.26	-0.0027	-0.0008	-0.0035	-0.0034	0.0001
Multiple myeloma	B.1.27	0.0003	-0.0001	0.0002	0.0004	0.0002
Leukemia	B.1.28	0.0098	-0.0005	0.0093	0.0120	0.0027
Other malignant neoplasms	B.1.29	0.0075	-0.0009	0.0066	0.0094	0.0027
Other neoplasms	B.1.30	-0.0001	-0.0005	-0.0006	-0.0002	0.0004
Cardiovascular diseases	B.2	0.5701	-0.0029	0.5671	0.7178	0.1506
Rheumatic heart disease	B.2.1	0.0684	-0.0001	0.0683	0.0850	0.0168
Ischemic heart disease	B.2.2	0.0641	-0.0015	0.0626	0.0808	0.0182
Stroke	B.2.3	0.3704	0.0017	0.3721	0.4673	0.0953
Hypertensive heart disease	B.2.4	0.0593	-0.0008	0.0584	0.0746	0.0162
Non-rheumatic valvular heart disease	B.2.5	0.0009	-0.0001	0.0008	0.0011	0.0003
Cardiomyopathy and myocarditis	B.2.6	0.0016	-0.0001	0.0015	0.0020	0.0005
Atrial fibrillation and flutter	B.2.8	0.0008	-0.0012	-0.0004	0.0010	0.0014
Aortic aneurysm	B.2.9	0.0007	0.0000	0.0007	0.0009	0.0002
Peripheral artery disease	B.2.10	0.0000	0.0002	0.0002	0.0000	-0.0002
Endocarditis	B.2.11	0.0006	0.0000	0.0005	0.0007	0.0001
Other cardiovascular and circulatory diseases	B.2.12	0.0034	-0.0010	0.0024	0.0043	0.0019
Chronic respiratory diseases	B.3	0.3215	0.0445	0.3660	0.4058	0.0398
Chronic obstructive pulmonary disease	B.3.1	0.3047	0.0391	0.3438	0.3849	0.0411
Pneumoconiosis	B.3.2	0.0046	0.0017	0.0063	0.0057	-0.0006
Asthma	B.3.3	0.0118	0.0038	0.0156	0.0147	-0.0009
Interstitial lung disease and pulmonary sarcoidosis	B.3.4	0.0004	-0.0001	0.0003	0.0005	0.0002
Other chronic respiratory diseases	B.3.5	0.0001	0.0000	0.0000	0.0001	0.0001
Digestive diseases	B.4	0.1503	0.0182	0.1685	0.1867	0.0182
Cirrhosis and other chronic liver diseases	B.4.1	0.0947	0.0015	0.0962	0.1173	0.0211
Upper digestive system diseases	B.4.2	0.0340	0.0082	0.0423	0.0424	0.0002
Appendicitis	B.4.3	0.0014	-0.0002	0.0012	0.0017	0.0005
Paralytic ileus and intestinal obstruction	B.4.4	0.0029	0.0000	0.0029	0.0036	0.0007
Inguinal, femoral, and abdominal hernia	B.4.5	0.0004	0.0007	0.0010	0.0005	-0.0006
Inflammatory bowel disease	B.4.6	0.0012	-0.0014	-0.0002	0.0015	0.0017
Vascular intestinal disorders	B.4.7	0.0004	0.0000	0.0004	0.0005	0.0001
Gallbladder and biliary diseases	B.4.8	0.0067	0.0090	0.0157	0.0084	-0.0073
Pancreatitis	B.4.9	0.0030	0.0001	0.0031	0.0037	0.0006
Other digestive diseases	B.4.10	0.0057	0.0002	0.0059	0.0071	0.0012
Neurological disorders	B.5	0.0076	-0.0141	-0.0065	0.0094	0.0159
Alzheimer's disease and other dementias	B.5.1	0.0008	-0.0019	-0.0011	0.0011	0.0022
Parkinson's disease	B.5.2	0.0029	-0.0020	0.0009	0.0037	0.0028
Idiopathic epilepsy	B.5.3	0.0027	-0.0014	0.0013	0.0032	0.0019
Multiple sclerosis	B.5.4	0.0003	-0.0001	0.0003	0.0004	0.0001
Motor neuron disease	B.5.5	0.0004	0.0000	0.0004	0.0005	0.0001
Headache disorders	B.5.6	0.0000	-0.0076	-0.0076	0.0000	0.0076
Other neurological disorders	B.5.7	0.0004	-0.0011	-0.0007	0.0005	0.0012

Causes	Level	ΔHALE			ΔLE	ΔLED
		Mortality Effect	Disability Effect	Total Effect	Total Effect	
Mental disorders	B.6	0.0000	-0.0068	-0.0068	0.0000	0.0068
Schizophrenia	B.6.1	0.0000	-0.0014	-0.0014	0.0000	0.0014
Depressive disorders	B.6.2	0.0000	-0.0113	-0.0113	0.0000	0.0113
Bipolar disorder	B.6.3	0.0000	-0.0001	-0.0001	0.0000	0.0001
Anxiety disorders	B.6.4	0.0000	0.0046	0.0046	0.0000	-0.0046
Eating disorders	B.6.5	0.0000	-0.0003	-0.0003	0.0000	0.0003
Autism spectrum disorders	B.6.6	0.0000	-0.0001	-0.0001	0.0000	0.0001
Attention-deficit/hyperactivity disorder	B.6.7	0.0000	0.0001	0.0001	0.0000	-0.0001
Conduct disorder	B.6.8	0.0000	0.0000	0.0000	0.0000	0.0000
Idiopathic developmental intellectual disability	B.6.9	0.0000	0.0015	0.0015	0.0000	-0.0015
Other mental disorders	B.6.10	0.0000	0.0001	0.0001	0.0000	-0.0001
Substance use disorders	B.7	0.0091	-0.0028	0.0062	0.0111	0.0048
Alcohol use disorders	B.7.1	-0.0009	-0.0009	-0.0019	-0.0010	0.0008
Drug use disorders	B.7.2	0.0100	-0.0019	0.0081	0.0121	0.0040
Diabetes and kidney diseases	B.8	0.0380	-0.0268	0.0112	0.0472	0.0359
Diabetes mellitus	B.8.1	0.0133	-0.0226	-0.0093	0.0168	0.0261
Chronic kidney disease	B.8.2	0.0207	-0.0042	0.0166	0.0255	0.0089
Acute glomerulonephritis	B.8.3	0.0040	0.0000	0.0040	0.0049	0.0009
Skin and subcutaneous diseases	B.9	0.0013	0.0016	0.0029	0.0016	-0.0013
Dermatitis	B.9.1	0.0000	-0.0003	-0.0003	0.0000	0.0003
Psoriasis	B.9.2	0.0000	0.0039	0.0039	0.0000	-0.0039
Bacterial skin diseases	B.9.3	0.0013	0.0000	0.0013	0.0016	0.0003
Scabies	B.9.4	0.0000	0.0002	0.0002	0.0000	-0.0002
Fungal skin diseases	B.9.5	0.0000	0.0001	0.0001	0.0000	-0.0001
Viral skin diseases	B.9.6	0.0000	0.0001	0.0001	0.0000	-0.0001
Acne vulgaris	B.9.7	0.0000	-0.0007	-0.0007	0.0000	0.0007
Alopecia areata	B.9.8	0.0000	0.0000	0.0000	0.0000	0.0000
Pruritus	B.9.9	0.0000	-0.0003	-0.0003	0.0000	0.0003
Urticaria	B.9.10	0.0000	-0.0001	-0.0001	0.0000	0.0001
Decubitus ulcer	B.9.11	-0.0001	0.0000	-0.0001	-0.0001	0.0000
Other skin and subcutaneous diseases	B.9.12	0.0000	-0.0013	-0.0012	0.0000	0.0013
Sense organ diseases	B.10	0.0000	0.0077	0.0077	0.0000	-0.0077
Blindness and vision loss	B.10.1	0.0000	0.0002	0.0002	0.0000	-0.0002
Age-related and other hearing loss	B.10.2	0.0000	0.0081	0.0081	0.0000	-0.0081
Other sense organ diseases	B.10.3	0.0000	-0.0006	-0.0006	0.0000	0.0006
Musculoskeletal disorders	B.11	0.0003	0.0215	0.0217	0.0003	-0.0214
Rheumatoid arthritis	B.11.1	0.0005	-0.0007	-0.0002	0.0006	0.0009
Osteoarthritis	B.11.2	0.0000	-0.0095	-0.0095	0.0000	0.0095
Low back pain	B.11.3	0.0000	0.0814	0.0814	0.0000	-0.0814
Neck pain	B.11.4	0.0000	-0.0090	-0.0090	0.0000	0.0090
Gout	B.11.5	0.0000	-0.0021	-0.0021	0.0000	0.0021
Other musculoskeletal disorders	B.11.6	-0.0002	-0.0385	-0.0388	-0.0003	0.0385

Causes	Level	ΔHALE			ΔLE	ΔLED
		Mortality Effect	Disability Effect	Total Effect	Total Effect	
Other non-communicable diseases	B.12	0.0069	0.0326	0.0395	0.0086	-0.0309
Congenital birth defects	B.12.1	0.0007	0.0017	0.0023	0.0008	-0.0015
Urinary diseases and male infertility	B.12.2	0.0033	0.0032	0.0065	0.0041	-0.0024
Gynecological diseases	B.12.3	-0.0001	0.0171	0.0170	-0.0001	-0.0171
Hemoglobinopathies and hemolytic anemias	B.12.4	0.0022	0.0117	0.0139	0.0027	-0.0112
Endocrine, metabolic, blood, and immune disorders	B.12.5	0.0009	-0.0027	-0.0018	0.0011	0.0029
Oral disorders	B.12.6	0.0000	0.0016	0.0016	0.0000	-0.0016
**Injuries**	C	0.1336	-0.0102	0.1234	0.1624	0.0390
Transport injuries	C.1	0.0251	-0.0199	0.0052	0.0302	0.0250
Road injuries	C.1.1	0.0203	-0.0212	-0.0009	0.0244	0.0253
Other transport injuries	C.1.2	0.0048	0.0013	0.0061	0.0058	-0.0003
Unintentional injuries	C.2	0.0271	0.0058	0.0328	0.0331	0.0002
Falls	C.2.1	0.0015	0.0040	0.0055	0.0019	-0.0036
Drowning	C.2.2	0.0090	0.0004	0.0094	0.0109	0.0015
Fire, heat, and hot substances	C.2.3	0.0025	0.0000	0.0025	0.0031	0.0005
Poisonings	C.2.4	0.0002	0.0000	0.0001	0.0002	0.0000
Exposure to mechanical forces	C.2.5	0.0034	-0.0037	-0.0003	0.0041	0.0044
Adverse effects of medical treatment	C.2.6	0.0027	0.0000	0.0027	0.0033	0.0006
Animal contact	C.2.7	0.0033	0.0010	0.0043	0.0040	-0.0002
Foreign body	C.2.8	0.0002	0.0031	0.0033	0.0003	-0.0031
Environmental heat and cold exposure	C.2.9	0.0037	0.0005	0.0042	0.0045	0.0003
Exposure to forces of nature	C.2.10	0.0004	-0.0005	-0.0001	0.0005	0.0006
Other unintentional injuries	C.2.11	0.0002	0.0009	0.0011	0.0003	-0.0008
Self-harm and interpersonal violence	C.3	0.0814	0.0039	0.0853	0.0991	0.0138
Self-harm	C.3.1	0.0724	0.0020	0.0744	0.0883	0.0139
Interpersonal violence	C.3.2	0.0083	0.0019	0.0102	0.0099	-0.0003
Conflict and terrorism	C.3.3	0.0000	0.0000	0.0000	0.0000	0.0000
Executions and police conflict	C.3.4	0.0008	0.0000	0.0008	0.0009	0.0002

Table S7d | Cause-specific effects of mortality and disability (years) on changes in life expectancy (LE) and health-adjusted life expectancy (HALE) for those aged 65+ years from 1990 to 2019.

Causes	Level	ΔHALE			ΔLE	ΔLED
		Mortality Effect	Disability Effect	Total Effect	Total Effect	
**Communicable, maternal, neonatal, and nutritional diseases**	A	0.4670	0.0708	0.5378	0.3158	-0.2220
HIV/AIDS and sexually transmitted infections	A.1	-0.0035	-0.0009	-0.0044	-0.0034	0.0009
HIV/AIDS	A.1.1	-0.0041	-0.0009	-0.0050	-0.0039	0.0011
Sexually transmitted infections excluding HIV	A.1.2	0.0006	0.0000	0.0007	0.0005	-0.0002
Respiratory infections and tuberculosis	A.2	0.3966	0.0163	0.4129	0.2707	-0.1422
Tuberculosis	A.2.1	0.1455	0.0145	0.1600	0.1205	-0.0395
Lower respiratory infections	A.2.2	0.2258	0.0011	0.2269	0.1368	-0.0901
Upper respiratory infections	A.2.3	0.0253	-0.0001	0.0252	0.0133	-0.0118
Otitis media	A.2.4	0.0001	0.0007	0.0008	0.0001	-0.0007
Enteric infections	A.3	0.0241	0.0005	0.0246	0.0153	-0.0093
Diarrheal diseases	A.3.1	0.0239	0.0005	0.0244	0.0151	-0.0093
Typhoid and paratyphoid	A.3.2	0.0000	0.0000	0.0000	0.0000	0.0000
Invasive Non-typhoidal Salmonella (iNTS)	A.3.3	0.0001	0.0000	0.0001	0.0001	0.0000
Other intestinal infectious diseases	A.3.4	0.0001	0.0000	0.0000	0.0000	0.0000
Neglected tropical diseases and malaria	A.4	0.0026	0.0196	0.0222	0.0022	-0.0200
Malaria	A.4.1	0.0002	0.0001	0.0002	0.0002	-0.0001
Chagas disease	A.4.2	0.0000	0.0000	0.0000	0.0000	0.0000
Leishmaniasis	A.4.3	0.0000	0.0000	0.0000	0.0000	0.0000
African trypanosomiasis	A.4.4	0.0000	0.0000	0.0000	0.0000	0.0000
Schistosomiasis	A.4.5	0.0020	0.0004	0.0024	0.0017	-0.0007
Cysticercosis	A.4.6	0.0000	0.0027	0.0027	0.0000	-0.0027
Cystic echinococcosis	A.4.7	0.0000	0.0000	0.0000	0.0000	0.0000
Lymphatic filariasis	A.4.8	0.0000	0.0000	0.0000	0.0000	0.0000
Onchocerciasis	A.4.9	0.0000	0.0000	0.0000	0.0000	0.0000
Trachoma	A.4.10	0.0000	0.0022	0.0022	0.0000	-0.0022
Dengue	A.4.11	0.0000	-0.0002	-0.0002	0.0000	0.0002
Yellow fever	A.4.12	0.0000	0.0000	0.0000	0.0000	0.0000
Rabies	A.4.13	0.0001	0.0000	0.0001	0.0001	0.0000
Intestinal nematode infections	A.4.14	0.0001	0.0043	0.0044	0.0000	-0.0043
Food-borne trematodiases	A.4.15	0.0000	0.0091	0.0091	0.0000	-0.0091
Leprosy	A.4.16	0.0000	0.0000	0.0000	0.0000	0.0000
Ebola	A.4.17	0.0000	0.0000	0.0000	0.0000	0.0000
Zika virus	A.4.18	0.0000	0.0000	0.0000	0.0000	0.0000
Guinea worm disease	A.4.19	0.0000	0.0000	0.0000	0.0000	0.0000
Other neglected tropical diseases	A.4.20	0.0002	0.0011	0.0013	0.0002	-0.0011
Other infectious diseases	A.5	0.0264	0.0021	0.0284	0.0201	-0.0083
Meningitis	A.5.1	0.0049	0.0006	0.0055	0.0036	-0.0019
Encephalitis	A.5.2	0.0003	0.0002	0.0005	0.0003	-0.0002
Diphtheria	A.5.3	0.0000	0.0000	0.0000	0.0000	0.0000

Causes	Level	ΔHALE			ΔLE	ΔLED
		Mortality Effect	Disability Effect	Total Effect	Total Effect	
Whooping cough	A.5.4	0.0000	0.0000	0.0000	0.0000	0.0000
Tetanus	A.5.5	0.0033	0.0000	0.0033	0.0026	-0.0007
Measles	A.5.6	0.0000	0.0000	0.0000	0.0000	0.0000
Varicella and herpes zoster	A.5.7	0.0036	0.0000	0.0036	0.0018	-0.0018
Acute hepatitis	A.5.8	0.0120	0.0002	0.0121	0.0102	-0.0020
Other unspecified infectious diseases	A.5.9	0.0023	0.0011	0.0034	0.0016	-0.0018
Maternal and neonatal disorders	A.6	0.0000	-0.0081	-0.0081	0.0000	0.0081
Maternal disorders	A.6.1	0.0000	0.0000	0.0000	0.0000	0.0000
Neonatal disorders	A.6.2	0.0000	-0.0081	-0.0081	0.0000	0.0081
Nutritional deficiencies	A.7	0.0208	0.0413	0.0621	0.0109	-0.0512
Protein-energy malnutrition	A.7.1	0.0222	-0.0032	0.0190	0.0115	-0.0076
Iodine deficiency	A.7.2	0.0000	0.0003	0.0003	0.0000	-0.0003
Vitamin A deficiency	A.7.3	0.0000	0.0000	0.0000	0.0000	0.0000
Dietary iron deficiency	A.7.4	0.0000	0.0445	0.0445	0.0000	-0.0445
Other nutritional deficiencies	A.7.5	-0.0014	-0.0002	-0.0016	-0.0005	0.0010
**Non-communicable diseases**	B	3.6646	0.0146	3.6791	2.7537	-0.9254
Neoplasms	B.1	0.0653	-0.0265	0.0388	0.1150	0.0762
Lip and oral cavity cancer	B.1.1	-0.0032	-0.0003	-0.0035	-0.0026	0.0008
Nasopharynx cancer	B.1.2	0.0081	-0.0004	0.0077	0.0068	-0.0009
Other pharynx cancer	B.1.3	0.0005	0.0000	0.0005	0.0004	-0.0001
Esophageal cancer	B.1.4	0.0760	0.0007	0.0767	0.0726	-0.0040
Stomach cancer	B.1.5	0.1296	-0.0002	0.1294	0.1197	-0.0096
Colon and rectum cancer	B.1.6	-0.0520	-0.0075	-0.0595	-0.0372	0.0223
Liver cancer	B.1.7	0.1042	0.0014	0.1056	0.0939	-0.0118
Gallbladder and biliary tract cancer	B.1.8	-0.0034	-0.0001	-0.0036	-0.0024	0.0012
Pancreatic cancer	B.1.9	-0.0324	-0.0005	-0.0329	-0.0255	0.0074
Larynx cancer	B.1.10	0.0025	-0.0003	0.0021	0.0023	0.0002
Tracheal, bronchus, and lung cancer	B.1.11	-0.1490	-0.0040	-0.1530	-0.1055	0.0475
Malignant skin melanoma	B.1.12	0.0002	-0.0001	0.0001	0.0002	0.0001
Non-melanoma skin cancer	B.1.13	-0.0030	-0.0001	-0.0031	-0.0017	0.0014
Breast cancer	B.1.14	-0.0071	-0.0029	-0.0100	-0.0057	0.0043
Cervical cancer	B.1.15	0.0041	0.0000	0.0041	0.0035	-0.0005
Uterine cancer	B.1.16	0.0046	-0.0001	0.0044	0.0040	-0.0005
Ovarian cancer	B.1.17	-0.0048	-0.0002	-0.0050	-0.0044	0.0006
Prostate cancer	B.1.18	-0.0010	-0.0038	-0.0047	0.0010	0.0057
Testicular cancer	B.1.19	-0.0001	-0.0001	-0.0002	-0.0001	0.0001
Kidney cancer	B.1.20	-0.0065	-0.0004	-0.0069	-0.0048	0.0021
Bladder cancer	B.1.21	0.0028	-0.0012	0.0017	0.0025	0.0008
Brain and central nervous system cancer	B.1.22	-0.0038	-0.0003	-0.0041	-0.0026	0.0015
Thyroid cancer	B.1.23	-0.0002	-0.0002	-0.0003	0.0000	0.0003
Mesothelioma	B.1.24	0.0000	0.0000	0.0000	0.0000	0.0000
Hodgkin lymphoma	B.1.25	0.0023	-0.0001	0.0023	0.0021	-0.0002

Causes	Level	ΔHALE			ΔLE	ΔLED
		Mortality Effect	Disability Effect	Total Effect	Total Effect	
Non-Hodgkin lymphoma	B.1.26	-0.0061	-0.0019	-0.0079	-0.0053	0.0026
Multiple myeloma	B.1.27	-0.0006	-0.0002	-0.0008	-0.0004	0.0004
Leukemia	B.1.28	0.0031	-0.0007	0.0025	0.0030	0.0005
Other malignant neoplasms	B.1.29	0.0013	-0.0022	-0.0009	0.0019	0.0028
Other neoplasms	B.1.30	-0.0007	-0.0010	-0.0016	-0.0005	0.0012
Cardiovascular diseases	B.2	1.0592	-0.0771	0.9821	0.9082	-0.0739
Rheumatic heart disease	B.2.1	0.1484	-0.0003	0.1481	0.1085	-0.0396
Ischemic heart disease	B.2.2	-0.3870	-0.0022	-0.3892	-0.1663	0.2228
Stroke	B.2.3	0.9916	-0.0771	0.9145	0.7485	-0.1660
Hypertensive heart disease	B.2.4	0.2947	0.0049	0.2996	0.2075	-0.0921
Non-rheumatic valvular heart disease	B.2.5	0.0032	-0.0010	0.0022	0.0020	-0.0002
Cardiomyopathy and myocarditis	B.2.6	-0.0028	0.0001	-0.0027	-0.0007	0.0020
Atrial fibrillation and flutter	B.2.8	0.0027	-0.0027	0.0000	0.0025	0.0025
Aortic aneurysm	B.2.9	0.0005	0.0000	0.0005	0.0005	0.0000
Peripheral artery disease	B.2.10	-0.0004	0.0014	0.0010	-0.0002	-0.0012
Endocarditis	B.2.11	0.0012	0.0000	0.0011	0.0008	-0.0003
Other cardiovascular and circulatory diseases	B.2.12	0.0071	-0.0002	0.0070	0.0052	-0.0017
Chronic respiratory diseases	B.3	2.3184	0.0559	2.3743	1.5502	-0.8241
Chronic obstructive pulmonary disease	B.3.1	2.2457	0.0489	2.2946	1.4994	-0.7952
Pneumoconiosis	B.3.2	0.0080	-0.0005	0.0075	0.0069	-0.0006
Asthma	B.3.3	0.0657	0.0085	0.0743	0.0444	-0.0299
Interstitial lung disease and pulmonary sarcoidosis	B.3.4	-0.0004	-0.0009	-0.0013	-0.0001	0.0013
Other chronic respiratory diseases	B.3.5	-0.0006	-0.0001	-0.0007	-0.0003	0.0003
Digestive diseases	B.4	0.2080	0.0202	0.2282	0.1597	-0.0685
Cirrhosis and other chronic liver diseases	B.4.1	0.0663	0.0008	0.0671	0.0580	-0.0092
Upper digestive system diseases	B.4.2	0.0673	0.0072	0.0745	0.0513	-0.0233
Appendicitis	B.4.3	0.0042	-0.0001	0.0041	0.0029	-0.0012
Paralytic ileus and intestinal obstruction	B.4.4	0.0116	-0.0001	0.0116	0.0079	-0.0037
Inguinal, femoral, and abdominal hernia	B.4.5	0.0019	0.0000	0.0019	0.0013	-0.0006
Inflammatory bowel disease	B.4.6	0.0068	-0.0008	0.0060	0.0041	-0.0020
Vascular intestinal disorders	B.4.7	0.0011	0.0000	0.0011	0.0008	-0.0002
Gallbladder and biliary diseases	B.4.8	0.0241	0.0125	0.0366	0.0169	-0.0197
Pancreatitis	B.4.9	0.0036	0.0001	0.0037	0.0029	-0.0007
Other digestive diseases	B.4.10	0.0211	0.0005	0.0217	0.0137	-0.0079
Neurological disorders	B.5	0.0172	-0.0494	-0.0322	0.0143	0.0466
Alzheimer's disease and other dementias	B.5.1	0.0035	-0.0384	-0.0349	0.0032	0.0381
Parkinson's disease	B.5.2	0.0130	-0.0052	0.0077	0.0103	0.0026
Idiopathic epilepsy	B.5.3	0.0009	-0.0020	-0.0011	0.0008	0.0019
Multiple sclerosis	B.5.4	0.0001	0.0000	0.0001	0.0001	0.0000
Motor neuron disease	B.5.5	0.0001	0.0000	0.0001	0.0001	0.0000
Headache disorders	B.5.6	0.0000	-0.0027	-0.0027	0.0000	0.0027
Other neurological disorders	B.5.7	-0.0004	-0.0010	-0.0014	-0.0002	0.0012

Causes	Level	ΔHALE Mortality Effect	ΔHALE Disability Effect	ΔHALE Total Effect	ΔLE Total Effect	ΔLED
Mental disorders	B.6	0.0000	-0.0151	-0.0151	0.0000	0.0151
Schizophrenia	B.6.1	0.0000	-0.0007	-0.0007	0.0000	0.0007
Depressive disorders	B.6.2	0.0000	-0.0200	-0.0200	0.0000	0.0200
Bipolar disorder	B.6.3	0.0000	0.0000	0.0000	0.0000	0.0000
Anxiety disorders	B.6.4	0.0000	0.0054	0.0054	0.0000	-0.0054
Eating disorders	B.6.5	0.0000	0.0000	0.0000	0.0000	0.0000
Autism spectrum disorders	B.6.6	0.0000	-0.0002	-0.0002	0.0000	0.0002
Attention-deficit/hyperactivity disorder	B.6.7	0.0000	0.0000	0.0000	0.0000	0.0000
Conduct disorder	B.6.8	0.0000	0.0000	0.0000	0.0000	0.0000
Idiopathic developmental intellectual disability	B.6.9	0.0000	0.0007	0.0007	0.0000	-0.0007
Other mental disorders	B.6.10	0.0000	-0.0002	-0.0002	0.0000	0.0002
Substance use disorders	B.7	0.0053	-0.0059	-0.0006	0.0045	0.0052
Alcohol use disorders	B.7.1	-0.0004	-0.0034	-0.0038	-0.0003	0.0035
Drug use disorders	B.7.2	0.0056	-0.0025	0.0032	0.0049	0.0017
Diabetes and kidney diseases	B.8	-0.0238	-0.0167	-0.0405	-0.0083	0.0322
Diabetes mellitus	B.8.1	-0.0212	-0.0142	-0.0354	-0.0094	0.0259
Chronic kidney disease	B.8.2	-0.0086	-0.0026	-0.0112	-0.0037	0.0076
Acute glomerulonephritis	B.8.3	0.0061	0.0000	0.0061	0.0048	-0.0013
Skin and subcutaneous diseases	B.9	0.0093	0.0008	0.0102	0.0050	-0.0052
Dermatitis	B.9.1	0.0000	0.0000	0.0000	0.0000	0.0000
Psoriasis	B.9.2	0.0000	0.0027	0.0027	0.0000	-0.0027
Bacterial skin diseases	B.9.3	0.0103	0.0000	0.0103	0.0053	-0.0049
Scabies	B.9.4	0.0000	0.0002	0.0002	0.0000	-0.0002
Fungal skin diseases	B.9.5	0.0000	0.0001	0.0001	0.0000	-0.0001
Viral skin diseases	B.9.6	0.0000	-0.0001	-0.0001	0.0000	0.0001
Acne vulgaris	B.9.7	0.0000	-0.0002	-0.0002	0.0000	0.0002
Alopecia areata	B.9.8	0.0000	0.0000	0.0000	0.0000	0.0000
Pruritus	B.9.9	0.0000	-0.0003	-0.0003	0.0000	0.0003
Urticaria	B.9.10	0.0000	0.0000	0.0000	0.0000	0.0000
Decubitus ulcer	B.9.11	-0.0012	-0.0003	-0.0015	-0.0006	0.0009
Other skin and subcutaneous diseases	B.9.12	0.0003	-0.0012	-0.0009	0.0002	0.0011
Sense organ diseases	B.10	0.0000	0.0391	0.0391	0.0000	-0.0391
Blindness and vision loss	B.10.1	0.0000	0.0301	0.0301	0.0000	-0.0301
Age-related and other hearing loss	B.10.2	0.0000	0.0099	0.0099	0.0000	-0.0099
Other sense organ diseases	B.10.3	0.0000	-0.0008	-0.0008	0.0000	0.0008
Musculoskeletal disorders	B.11	-0.0029	0.0574	0.0545	-0.0013	-0.0558
Rheumatoid arthritis	B.11.1	-0.0014	-0.0004	-0.0018	-0.0005	0.0012
Osteoarthritis	B.11.2	0.0000	-0.0113	-0.0113	0.0000	0.0113
Low back pain	B.11.3	0.0000	0.0997	0.0997	0.0000	-0.0997
Neck pain	B.11.4	0.0000	-0.0058	-0.0058	0.0000	0.0058
Gout	B.11.5	0.0000	-0.0038	-0.0038	0.0000	0.0038
Other musculoskeletal disorders	B.11.6	-0.0015	-0.0210	-0.0225	-0.0008	0.0218

Causes	Level	ΔHALE			ΔLE	ΔLED
		Mortality Effect	Disability Effect	Total Effect	Total Effect	
Other non-communicable diseases	B.12	0.0085	0.0319	0.0404	0.0064	-0.0339
Congenital birth defects	B.12.1	0.0000	0.0009	0.0010	0.0001	-0.0009
Urinary diseases and male infertility	B.12.2	0.0080	0.0019	0.0099	0.0054	-0.0045
Gynecological diseases	B.12.3	-0.0002	0.0066	0.0064	-0.0001	-0.0065
Hemoglobinopathies and hemolytic anemias	B.12.4	0.0024	0.0135	0.0159	0.0020	-0.0139
Endocrine, metabolic, blood, and immune disorders	B.12.5	-0.0018	0.0038	0.0021	-0.0009	-0.0030
Oral disorders	B.12.6	0.0000	0.0051	0.0051	0.0000	-0.0051
**Injuries**	C	0.0534	-0.0561	-0.0026	0.0550	0.0576
Transport injuries	C.1	-0.0019	-0.0285	-0.0304	-0.0001	0.0303
Road injuries	C.1.1	-0.0040	-0.0295	-0.0335	-0.0019	0.0316
Other transport injuries	C.1.2	0.0021	0.0010	0.0031	0.0018	-0.0013
Unintentional injuries	C.2	-0.0076	-0.0295	-0.0372	0.0042	0.0414
Falls	C.2.1	-0.0275	-0.0284	-0.0560	-0.0112	0.0448
Drowning	C.2.2	0.0056	-0.0003	0.0054	0.0048	-0.0006
Fire, heat, and hot substances	C.2.3	0.0058	-0.0001	0.0057	0.0039	-0.0017
Poisonings	C.2.4	-0.0023	0.0000	-0.0023	-0.0016	0.0007
Exposure to mechanical forces	C.2.5	-0.0002	-0.0044	-0.0046	0.0000	0.0046
Adverse effects of medical treatment	C.2.6	0.0039	0.0000	0.0039	0.0029	-0.0010
Animal contact	C.2.7	0.0021	0.0009	0.0030	0.0019	-0.0011
Foreign body	C.2.8	-0.0014	0.0023	0.0009	-0.0007	-0.0016
Environmental heat and cold exposure	C.2.9	0.0064	0.0005	0.0070	0.0041	-0.0028
Exposure to forces of nature	C.2.10	0.0003	-0.0004	-0.0001	0.0002	0.0003
Other unintentional injuries	C.2.11	-0.0004	0.0004	0.0000	-0.0003	-0.0003
Self-harm and interpersonal violence	C.3	0.0630	0.0020	0.0650	0.0509	-0.0141
Self-harm	C.3.1	0.0603	0.0015	0.0619	0.0483	-0.0135
Interpersonal violence	C.3.2	0.0026	0.0005	0.0031	0.0025	-0.0006
Conflict and terrorism	C.3.3	0.0000	0.0000	0.0000	0.0000	0.0000
Executions and police conflict	C.3.4	0.0001	0.0000	0.0001	0.0001	0.0000
